# Single-nucleus RNA-seq2 reveals functional crosstalk between liver zonation and ploidy

**DOI:** 10.1038/s41467-021-24543-5

**Published:** 2021-07-12

**Authors:** M. L. Richter, I. K. Deligiannis, K. Yin, A. Danese, E. Lleshi, P. Coupland, C. A. Vallejos, K. P. Matchett, N. C. Henderson, M. Colome-Tatche, C. P. Martinez-Jimenez

**Affiliations:** 1 Helmholtz Pioneer Campus (HPC), Helmholtz Zentrum München, Neuherberg, Germany; 2 University of Cambridge, Cancer Research UK Cambridge Institute, Robinson Way, Cambridge, United Kingdom; 3 Institute of Computational Biology, Helmholtz Zentrum München, Neuherberg, Germany; 4 MRC Human Genetics Unit, Institute of Genetics & Molecular Medicine, University of Edinburgh, Western General Hospital, Edinburgh, United Kingdom; 5 Centre for Inflammation Research, The Queen’s Medical Research Institute, University of Edinburgh, Little France Crescent, Edinburgh, United Kingdom; 6 TUM School of Life Sciences Weihenstephan, Technical University of Munich, Freising, Germany; 7 Biomedical Center (BMC), Physiological Chemistry, Faculty of Medicine, LMU Munich, Munich, Germany; 8 TUM School of Medicine, Technical University of Munich, Munich, Germany

**Keywords:** Fluorescence in situ hybridization, RNA sequencing, RNA sequencing, Gene regulation, Transcriptomics

## Abstract

Single-cell RNA-seq reveals the role of pathogenic cell populations in development and progression of chronic diseases. In order to expand our knowledge on cellular heterogeneity, we have developed a single-nucleus RNA-seq2 method tailored for the comprehensive analysis of the nuclear transcriptome from frozen tissues, allowing the dissection of all cell types present in the liver, regardless of cell size or cellular fragility. We use this approach to characterize the transcriptional profile of individual hepatocytes with different levels of ploidy, and have discovered that ploidy states are associated with different metabolic potential, and gene expression in tetraploid mononucleated hepatocytes is conditioned by their position within the hepatic lobule. Our work reveals a remarkable crosstalk between gene dosage and spatial distribution of hepatocytes.

## Abstract (web summary)

Single-cell RNA-seq reveals the cellular heterogeneity in development and disease. Here the authors present a single-nucleus RNA-seq2 that allows deep characterization of nuclei isolated from frozen archived tissues, apply it for transcriptional profiling of individual hepatocytes, and determine a functional crosstalk between liver zonation and ploidy.

## Introduction

The liver performs a wide variety of physiological functions, including the metabolism of xenobiotics^1–5^ and the regulation of energy homeostasis^6,7^, among others. These functions are mostly performed by parenchymal hepatocytes which constitute about 70% of the liver mass. Liver non-parenchymal cells (NPCs), namely, liver sinusoidal endothelial cells, biliary cells, Kupffer cells, hepatic stellate cells, and immune cell populations constitute the remaining 30% of cell types. All these cells are organized in repetitive structures known as liver lobules^8,9^. The analysis of individual cells by single-cell genomics is changing our understanding of liver homeostasis and pathogenic conditions by taking into account their spatial distribution^10–16^. However, single-cell isolations from the liver require harsh enzymatic or mechanical dissociation protocols that perturb mRNA levels^17^. In particular, the two-step collagenase perfusion generally used to isolate hepatocytes from human livers leads to the downregulation of liver-specific transcription factors such as *Hnf4a*, *Cebpa*, *Hnf1a*, and *Foxa3*^18^, as well as their downstream target genes such as *Cyp2c9*, *Cyp2e1*, *Cyp2B6*, *Cyp2D6*, *Cyp3A5,* and *Cyp3A4*^17,19^.

Single-nucleus RNA-seq (snRNA-seq) has emerged as a complementary approach to study complex tissues at single-cell level^20,21^, including brain^21–27^, lung^28^, kidney^29–32^, liver^33^, and heart^34,35^ in mouse and human frozen samples^36,37^. However, there are no snRNA-seq methods tailored for frozen liver tissues. The nuclear transcriptome of individual cells has shown a high correlation to cytoplasmic RNA^23,38^, indicating that snRNA-seq is a powerful tool to study tissues from which intact and fresh cells are difficult to obtain. Here, we have developed a robust single-nucleus RNA-seq2 (snRNA-seq2) approach that relies on efficient lysis of the nuclear membrane. Our approach permits the unbiased characterization of all major cell types present in the liver from frozen archived samples with high resolution.

With snRNA-seq2, we explore at single-cell resolution, a defining feature of hepatocytes ploidy^39–44^. At birth, all hepatocytes are diploid, with a single nucleus containing two copies of each chromosome. During development, polyploidization gradually increases, leading to hepatocytes with several levels of ploidy. Hepatocyte ploidy depends on the DNA content of each nucleus (e.g., diploid, tetraploid, etc.) and the number of nuclei per hepatocyte (e.g., mono- or bi-nucleated)^41^. Here we present a thorough analysis of diploid (2n) and tetraploid (4n) nuclei from the mouse liver and demonstrate that ploidy is an additional source of hepatocyte heterogeneity, linking gene dosage and liver zonation.

## Results

### snRNA-seq2 allows deep and robust characterization of single nuclei isolated from frozen livers

In order to explore archived samples associated with health and disease conditions, we have developed a robust methodology that combines transcriptomics and efficient low-volume reactions in single nuclei isolated from frozen livers.

**Fig. 1:**
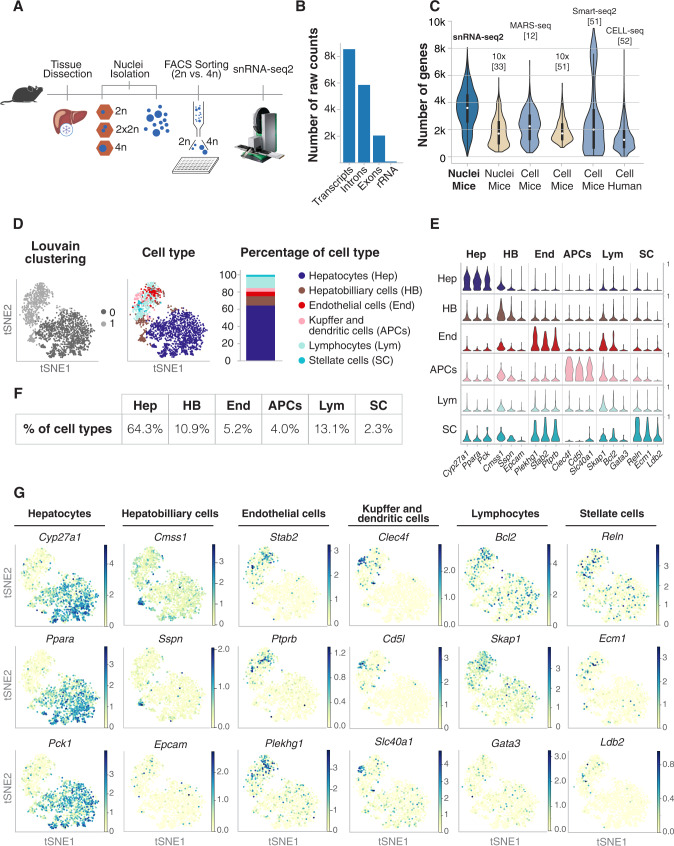
snRNA-seq2 on frozen tissue identifies and characterizes all major cell types present in the liver. **A** Schematic representation of the snRNA-seq2 pipeline. Nuclei are isolated from flash-frozen tissue, sorted by genome content in 384 well-plates, and subjected to the generation of full-length cDNA and sequencing. **B** Quality control of sequenced data shows the number of raw counts per genome feature in the nuclear transcriptome. **C** Violin plots comparing the high number of genes detected using snRNA-seq2 to publicly available single-cell RNA-seq datasets. Light brown: droplet-based methods (*n* = 10,395 nuclei [33]; *n* = 1,026 cells [51]), blue: plate-based approaches (*n* = 2,496 nuclei snRNA-seq2; *n* = 1,736 cells [12]; *n* = 981 cells [51]; *n* = 12,622 cells [52]). In the violin plot, boxplots indicate median number of genes per method (white dot). The lower and upper ends of the boxes correspond to first and third quartiles, respectively, while the whiskers extend to minimum (first quartile minus 1.5 × the inter-quartile range (IQR)), and maximum (third quartile plus 1.5 × IQR). Data points beyond the whiskers are only represented through their density distribution in the surrounding violin plots. For clearer visualization, the violins are cut at 0. **D** t-distributed stochastic neighborhood embedding (tSNE) of the nuclei by low-resolution *Louvain* clusters (left), with cell-type annotation for all major cell types (middle), and bar plot showing the percentage of all cell types (right) identified by snRNAseq2. **E** Stacked violin plot depicting selected illustrative marker genes per cell type (*y*-axis: scaled, log-transformed, normalized counts). **F** Percentage of cells types identified by snRNA-seq2. **G** tSNE colored by marker gene expression revealed cellular heterogeneity in all major cell population of the mouse liver.

Purified diploid (2n) and tetraploid (4n) nuclei were FACS sorted according to their genome content, using a gating strategy as previously described^39^ and followed by a modified version of Smart-seq chemistry using liquid handling robots for volume miniaturization (Fig. 1A, see “Methods”). This approach allows the detection of over 550 000 reads per nucleus, leading to an average detection of more than 11 000 transcripts (~4000 genes) per nucleus (Supp. Fig. 1). As expected for the nuclear transcriptome, the percentage of reads mapping to intronic regions was more than 68%, and the ribosomal reads were 1.2% (Fig. 1B). To show the robustness and reproducibility of this method, four different mice of three months old and C57BL/6J background were used (Animal ID). Different nuclei isolations were performed from the same liver (Plate ID). Two technical replicates were used to assess technical variability due to sequencing and were removed for downstream analysis (Technical replicate) (Supp. Fig. 1A–D, Supp Dataset 1 and “Methods”). Additionally, ERCC RNA spike-in mix was used to address technical noise and account for plate effects (including library preparation and sequencing)^45,46^. ERCC normalization was used to make counts comparable across cells and minimize results purely driven by ploidy^47–50^ (“Methods”; Supp. Fig. 1A). SnRNA-seq2 detected approximately 4000 genes per nucleus, which is a high number of genes detected per nucleus compared with other single-cell approaches in which intact cells are isolated from livers^12,51,52^ (Fig. 1C and Supp. Fig. 1E). Furthermore, our approach showed a *Pearson* correlation of 0.62 between gene expression in the nuclei using our snRNA-seq2 and scRNA-seq from intact cells isolated from fresh livers (Supp. Fig. 1F). This correlation shows that single-nucleus RNA-seq2 is a robust and reliable approach to study transcriptional profiles of individual cells from archived frozen tissues^20,23,25,37,53–55^.

The main improvement of our approach relies on the addition of a supplementary lysis buffer compatible with the generation of full-length cDNA and library preparation without the need for additional clean-up steps. This second lysis buffer (LB2) is compatible with a wide range of commercially available platforms and chemistries, including C1 Fluidigm (Fluidigm), well-plate approaches combined with liquid handling robots (Mosquito HV, TTP Labtech), as well as both SMARTer and NEBNext commercially available chemistries (Supp. Fig. 2 and Supp. Dataset 2). For instance, we detected 3.6-fold more genes per nucleus with our additional LB2 compared with classic approaches using SMARTer chemistry (Supp. Fig. 2C, D) and snRNA-seq2 performed better than other droplet-based approaches (Supp. Fig. 2E, F).

T-distributed stochastic neighbor embedding (t-SNE) was used to visualize nuclei FACS-sorted in an unbiased manner (Fig. 1D). Low-resolution *Louvain* clustering showed that the nuclei were clustering into two groups (0, 1), corresponding to hepatocytes and non-hepatocyte cells (Fig. 1D, left). After higher resolution clustering and identification of the top differential expressed genes between clusters, we identified hepatocytes (64.3%), hepatobiliary cells (10.9%), Kupffer and dendritic cells (APCs, 4%), endothelial cells (5.2%), stellate cells (2.3%), and lymphocytes (13.1%) as main cell clusters (Fig. 1D, right and Fig. 1E; Supp. Fig. 1F, G, Supp. Dataset 1 and Supp. Dataset 2)^11,12,52,56^. The expression distribution of key markers characteristic of those cell types showed that all liver cell populations can be identified with this methodology (Fig. 1F). Additionally, visualization of key representative markers such as *Cyp27a1*, *Ppara,* and *Pck1* for hepatocytes; *Sspn, Cmss1,* and *Epcam* for hepatobiliary cells; *Plekhg1, Stab2,* and *Ptprb* for endothelial cells; *Clec4f*, *Cd5l*, and *Slc40a1* for APCs; *Bcl2*, *Skap1,* and *Gata3* for lymphocytes; and *Reln, Ecm1,* and *Ldb2* for stellate cell showed that transcriptional heterogeneity can be captured from frozen liver tissues for each cell type (Fig. 1G). Higher resolution clustering on non-hepatocytes identified additional three cell types including *Epcam*-positive epithelial cells (0.73%), and different sub-groups of lymphocytes, including T—(4.00%), Plasma B—(4.61%), and B-cells (4.49%) (Fig. 1D, right; Supp. Fig. 3, Supp. Dataset 3 and Supp. Dataset 4).

**Fig. 2:**
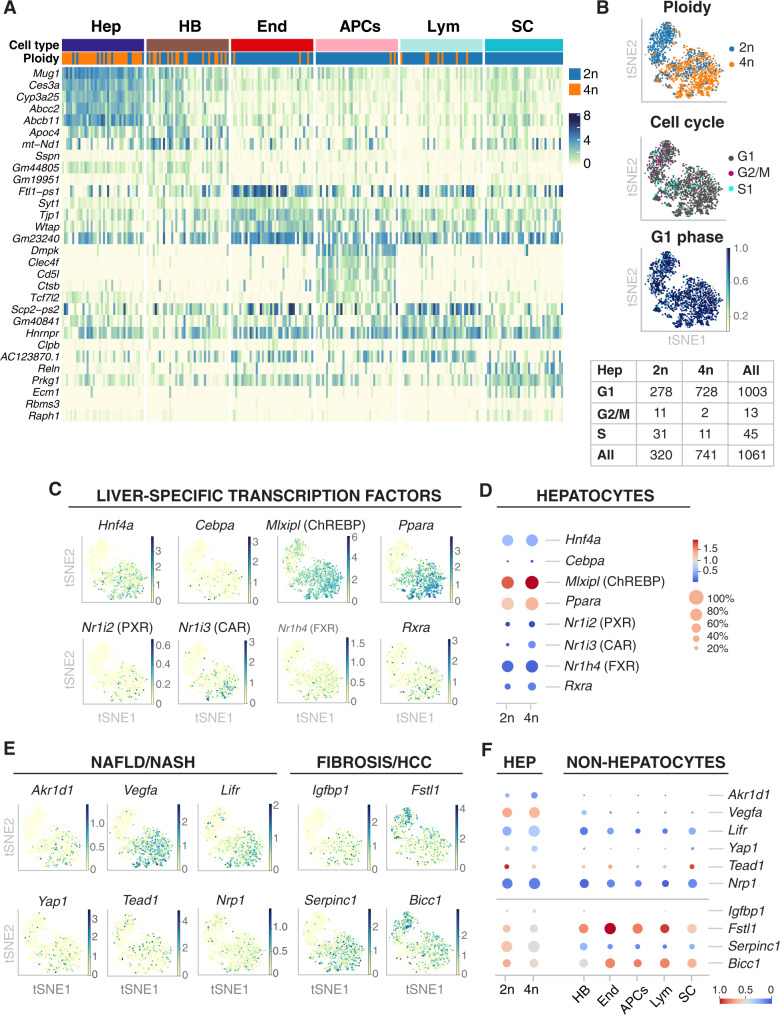
snRNA-seq2 allows deep profiling of single nuclei including key liver-specific transcription factors and downstream target genes involved in healthy homeostasis and chronic liver disease. **A** Heatmap showing the gene expression of the top five differentially expressed genes in forty randomly selected nuclei per cell type (colored by log-transformed, normalized counts). Ploidy analysis shows that 4n nuclei are enriched in the hepatocyte cluster. **B** Cell cycle analysis using *Cyclone* shows that the majority of nuclei are in G1 phase. tSNEs colored by ploidy (top), assigned cell cycle phase (middle), and G1 score (bottom). Table showing the number of nuclei that are in each assigned phase for diploid and tetraploid nuclei from the hepatocyte cluster. **C** tSNE colored by the expression of key liver-specific transcription factors involved in liver homeostasis and hepatic function. **D** Dot plot shows that the expression levels (color scale) and the percentage (dot size) of cells expressing key transcription factors can be dissected between 2n and 4n nuclei. **E** tSNEs colored by the expression of disease-related marker genes, separated into two main categories of liver disease: non-alcoholic fatty liver disease/non-alcoholic steatohepatitis (NAFLD/NASH) and fibrosis/hepatocellular carcinoma (HCC), showing cellular heterogeneity in different cell populations. **F** Dot plot showing the expression of the disease-related marker genes across cell types in young wild-type mice livers.

Further in-depth characterization of liver cell populations revealed additional features related to their genome content and respective ploidy levels. Heat map of the top five differentially expressed genes showed that hepatocytes and hepatobiliary cells were mainly composed of 2n and 4n nuclei while other cell types were primarily associated to 2n nuclei (Fig. 2A and Supp. Fig. 4A).

Consistent with the polyploid nature of hepatocytes^39,57^, we found as expected, that the hepatocyte cluster was enriched with a mixture of 2n and 4n nuclei (Fig. 2A). Nonetheless, few 4n nuclei were found in the non-hepatocyte clusters. Thus, we investigated whether cell cycle state could explain the presence of 4n nuclei in non-hepatocyte clusters (Fig. 2B and Supp. Fig. 4B–D)^45,58,59^. Cyclone was used to identify nuclei associated with G1, G2/M, and S phase of cell cycle^59^. As expected in the liver tissue, the majority of nuclei were in G1 phase (Fig. 2B Supp. Fig. 4B–D). This resulted in 94.5% of the hepatocyte cluster assigned to G1 phase, while 85.5% of nuclei in the non-hepatocyte cluster were in G1 phase. The number of 4n nuclei in the non-hepatocyte cluster was below 7%, which could be explained either by cell cycle stage (11 nuclei were in division) or technical bias during FACS sorting (e.g., nuclei tend to clump together). Therefore, FACS sorting of nuclei according to their DNA content is a robust and accurate strategy to analyze hepatocytes with different levels of ploidy^39^.

These results demonstrate that our method is highly sensitive with regards to the identification of the main cell types and simultaneous examination of the cellular heterogeneity present in intact frozen liver tissues.

### snRNA-seq2 uncovers transcription factors and rate-limiting enzymes involved in chronic liver disease

In order to investigate whether the nuclear transcriptome can be used to address functional responses in the context of health and disease, we studied the expression levels of key liver-specific transcription factors (e.g., *Hnf4a*, *Ppar*a, *Mlxpl*/ChREBP, and *Cebpa*), nuclear receptors (e.g., *Rxa*, *Nrfl2*/PXR, *Nr1i3*/CAR, and *Nrfh4*/FXR) and coactivators (e.g., *Ppargc1a*/PGC1A, Crebbp/CBP/p300, and Ncoa1/SRC-1)^1–3,6,60^ (Fig. 2C and Supp. Fig. 5A). Our approach allowed deep characterization of the expression levels of upstream metabolic regulators in hepatocytes bearing two (2n) and four (4n) complete genomes. We inspected the gene expression levels of key transcription factors and downstream effectors between 2n and 4n nuclei isolated from hepatocytes (Fig. 2D and Supp. Fig. 5)). For instance, peroxisome proliferator-activated receptor α (*Ppara*) is the most abundant isoform expressed in hepatocytes with key roles in lipid metabolism and displays protective roles against non-alcoholic fatty liver disease (NAFLD)^61–63^. *Ppara* expression was upregulated in 4n nuclei in comparison to 2n nuclei (Fig. 2D; Supp. Fig. 5, Supp. Dataset 4 and Supp. Dataset 5). Similarly, Carbohydrate responsive element binding protein (ChREBP), encoded by the *Mlxipl* gene, is a carbohydrate-signaling transcription factor highly expressed in liver and adipose tissues and regulates the synthesis de novo of fatty acids^64–66^. *Mlxipl* is upregulated in 4n hepatocytes (Fig. 2D, Supp. Fig. 5, Supp. Dataset 4 and Supp. Dataset 5). The precise interplay between upstream transcription factors and rate-limiting enzymes during hepatic lipid and glucose metabolism is likely to determine the final outcome in NAFLD-induced chronic lipid overload. Therefore, investigating the functional differences between 2n and 4n hepatocytes is crucial to understand the development and progression of complex liver disease.

Recently, changes in the expression patterns of transcriptional regulators and downstream target genes have been associated with the development and progression of chronic liver diseases such as NAFLD^67,68^, non-alcoholic steatohepatitis (NASH)^69^, fibrosis^15^, and human hepatocellular carcinoma (HCC)^52^. From those studies, well-known markers associated with chronic liver disease, were selected to illustrate that our methodology is highly sensitive to detect those genes from the nuclear transcriptome (Fig. 2E, F and Supp. Fig. 5). In particular, dysregulation of *Akr1d1* has been associated with human NAFLD^68^, and upregulation of *Yap1* (paralogue of *Wwtr1*) in human and murine NASH liver^70^. Furthermore, *Tead1* has been proposed as a marker of NASH in murine mouse models^71^, whereas *Vegfa*, *Lifr*, and *Nrp1* have been associated with the intrahepatic ligand-receptor signaling network involved in the pathogenesis of NASH^69^ (Fig. 2E, F and Supp. Fig. 5B). Similarly, downregulation of *Bicc1* and *Fstl1* has been associated with advance fibrosis^72^. More recently, Aizarani et al.^52^ have shown that the perturbation of gene signatures in individual cells is associated with HCC, for instance, changes in *Serpin1c* and *Igfbp1* (Fig. 2E, F; Supp. Fig. 5B).

In summary, we are able to detect marker genes associated with chronic liver disease identified from bulk and single-cell transcriptomic analysis. This methodology has the potential to investigate previously archived frozen murine and human samples, and interrogate how changes in gene expression correlate with the development and progression of liver diseases, taking into account cellular crosstalk and signaling pathways.

### snRNA-seq2 reveals differential transcriptional variability between 2n and 4n nuclei

Although recent single-cell transcriptomic studies have addressed cellular heterogeneity in the liver with respect to hepatocyte zonation^11,12,14,15,52^, and the variability among non-parenchymal cells during chronic liver disease^69,73–76^, little is known about the cellular heterogeneity in hepatocytes with different levels of ploidy^57,77–79^.

Polyploidy occurs in the liver during normal development^80^, and is also associated with pathological conditions such as cancer and chronic liver disease^81,82^. Moreover, ploidy increases with age^83–85^, as well as in chronic liver conditions^86^. More specifically, the presence of tetraploid mononucleated hepatocytes has been associated with poor prognosis in human hepatocellular carcinoma (HCC)^87,88^. Accordingly, we further characterized the transcriptional profile of individual 2n and 4n nuclei from the hepatocyte cluster and investigated whether their transcriptomic profile differs among those two populations.

**Fig. 3:**
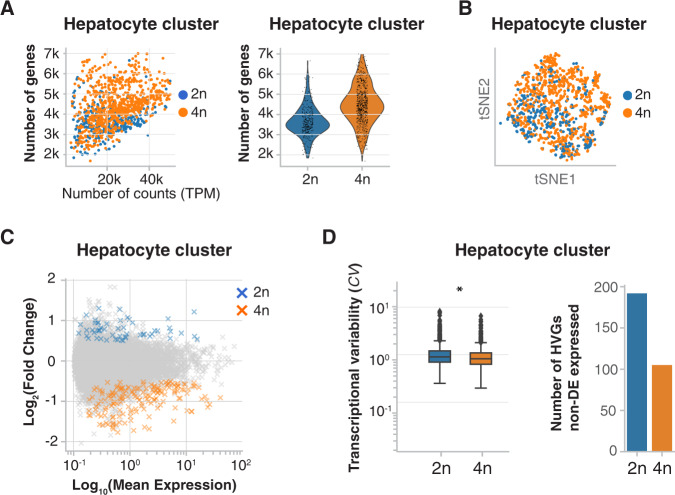
snRNA-seq2 reveals differences in gene expression between diploid and tetraploid hepatocytes. **A** Scatterplot showing the higher number of genes per gene-length normalized counts (TPM) detected in 4n hepatocytes compared with 2n hepatocytes (left). The violin plot showing that the number of genes in 4n nuclei is 1.25-times higher than in 2n hepatocytes (right). **B** tSNE embedding of the hepatocyte cluster showing that 2n and 4n nuclei are clustered together. **C** MA plot showing average logarithmically transformed mean expression (*x*-axis) *versus* the log_2_ fold change (*y*-axis) for pairwise comparison between 2n and 4n hepatocytes; 312 differentially expressed genes (DEGs) are depicted with crosses: 64 genes are upregulated in 2n nuclei (blue) and 248 genes are upregulated in 4n (orange). **D** Box plot of the coefficient of variation showing the transcriptional variability between 2n (*n* = 320) and 4n (*n* = 741) (left; two-sided Mann−Whitney U-test, *: *p*-value= 2.09e−14.). Box plots show the median coefficient of variation per ploidy status. The lower and upper ends of the boxes correspond to first and third quartiles, respectively, while the whiskers extend to minimum (first quartile minus 1.5 × the inter-quartile range (IQR)), and maximum (third quartile plus 1.5 × IQR). Data points beyond the whiskers are shown as individual points. Bar plot showing the higher number of highly variable genes (HVGs) that are not differentially expressed (non-DE) in 2n vs 4n (right).

Across all cell types, the median number of genes detected in 4n nuclei was 1.36-fold higher than in 2n nuclei (Supp. Fig. 6A and “Methods”). In the hepatocyte cluster, a 1.25-fold increase was detected in 4n compared to 2n nuclei (Fig. 3A), most likely due to the increase in cellular volume in 4n which has been associated with an increase in global transcription in larger cells^89^. We saw that 2n and 4n nuclei from the hepatocyte cluster do not separate in tSNE embedding based on their global gene expression profile (Fig. 3B and Supp. Fig. 6B); thus, they would be indistinguishable without a prior knowledge of their ploidy status. Herein, 2n and 4n nuclei from the hepatocyte cluster will be named 2n and 4n hepatocytes respectively.

Differential expression analysis showed that 2n and 4n hepatocytes are globally very similar. We detected 248 genes upregulated and 64 genes downregulated in 4n (Fig. 3C, Supp. Fig. 6C, D and Supp. Dataset 5). Only two of the 248 genes upregulated in 4n had zero counts in the 2n population (“Methods” and Supp. Fig. 6D, Supp. Dataset 5).

Subsequently, the transcriptional variability was estimated as the *coefficient of variation* using log-transformed data^90–92^ (Supp. Information). We confirmed that 4n hepatocytes showed a significantly lower *coefficient of variation* compared to 2n hepatocytes (2n are 1.09-times more variable than 4n, Mann–Whitney U-test, *p-*value 2.097e−14) (Fig. 3D and Supp. Dataset 6). Accordingly, the number of highly variable genes (HVGs) was higher in the 2n hepatocytes compared to the 4n hepatocytes (Fig. 3D and Supp. Dataset 6).

In summary, 2n and 4n hepatocytes showed a similar transcriptional profile in young mice; however, 312 DEGs were detected between these two cellular states. These results revealed the concealed cellular heterogeneity present in hepatocytes with different levels of ploidy.

### Tetraploid nuclei show extensive co-expression of liver stem cell markers

**Fig. 4:**
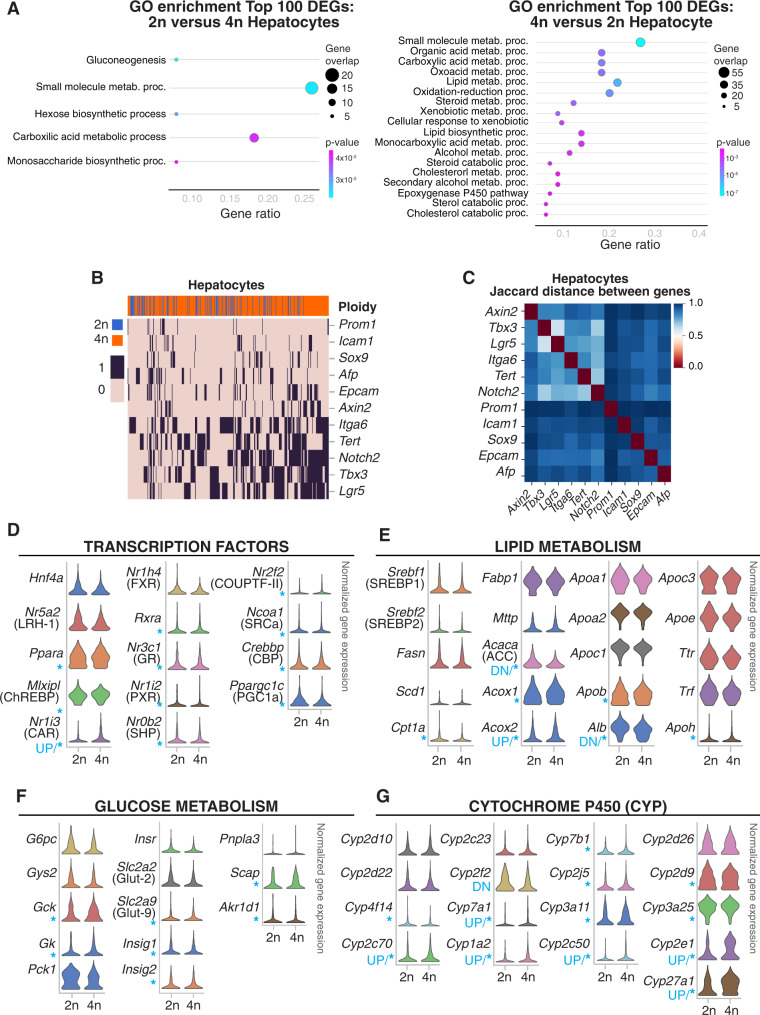
Functional characterization of 2n and 4n hepatocytes at the single-cell level. **A** Gene ontology (GO) analysis of the top 100 genes upregulated in 2n hepatocytes in comparison to 4n hepatocytes (left), and vice versa (right). **B** Heatmap showing co-expression of stem cell markers in hepatocytes (binary expression: 1-detected, 0-non-detected). Ordering of the cells and genes is based on hierarchical clustering between nuclei (columns), and stem cell markers (rows), revealing the stem cell markers are co-expressed in 2n and 4n hepatocytes. **C** Heatmap showing pairwise Jaccard distances between stem cell markers, reveals one main module of co-expression in hepatocytes. **D** Violin plots showing changes in gene expression level and distribution in key liver-specific transcription factors; **E** regulators of the hepatic lipid metabolism; **F** regulators of hepatic glucose metabolism and; **G** representative genes from the cytochrome P450 family involved in the xenobiotic metabolism of 2n and 4n hepatocytes. (*y*-axis: scaled, log-transformed, normalized counts; *: significant changes in gene expression distribution, UP: significant upregulation in 4n, DN: significant downregulation in 4n; Bonferroni-adjusted *p*-value<0.05; specific *p*-values can be found in Supplementary Dataset 4 and 7).

Polyploid hepatocytes have been associated with terminal differentiation and senescence^41^. However, a growing body of evidence indicates that polyploid hepatocytes retain their proliferative potential^79,93–96^. To further analyze the functional characteristics of 2n and 4n hepatocytes, Gene Ontology (GO) analysis was first performed on significant DEGs (Supp. Fig. 7A and Supp. Dataset 5). DEG in 2n hepatocytes were enriched in one category (i.e., small molecule metabolic process), while DEG in 4n hepatocytes were enriched in twenty different processes (Supp. Fig. 7A and Supp. Dataset 5). Secondly, we extended our GO analysis to the top hundred most differentially expressed genes (Fig. 4A and Supp. Dataset 5). Comparisons between 2n and 4n hepatocytes showed that 4n hepatocytes were further enriched in pathways involved in lipid, cholesterol, and xenobiotic metabolism (Fig. 4A and Supp. Dataset 5).

In order to further characterize the basal differences between 2n and 4n hepatocytes, we studied the regenerative and proliferative properties of these two cellular states. It has been suggested that diploid hepatocytes, located in proximity to the central vein of the liver lobule, act as stem cells during homeostasis and in response to injury^97^. However, it has recently been shown using a multicolor reporter allele system that polyploid hepatocytes proliferate in chronically injured livers^79,93,98^. Independent studies by Su *et al*. using AXIN2 lineage tracing have shown that hepatocytes upregulate *Axin2* and *Lgr5* after injury throughout the liver lobe^79,93,98^. To investigate if there are specific subpopulations of cells with stem cell properties enriched in 2n or 4n hepatocytes, several stem/progenitor cell-like marker genes were selected, including *Icam1*, *Afp*, *Sox9*, *Epcam*, *Axin2*, *Tbx3*, *Itga6*, *Tert*, *Lgr5,* and *Notch2*^78^ (Fig. 4B and Supp. Fig. 7B). Five of these markers, *Notch2*, *Tbx3*, *Itga6*, *Lgr5,* and *Tert* were expressed in more than 40% of the nuclei analyzed (Supp. Fig. 7C). While we observed that both 2n and 4n hepatocytes expressed several stem/progenitor markers, we did not find an enrichment of those genes in diploid hepatocytes (Fig. 4B and Supp. Fig. 7C). In order to investigate whether these markers were co-expressed in the same nuclei, the Jaccard index was used to measure the probability of those genes being co-expressed in the same nucleus^99,100^. In particular, the Jaccard distance measures dissimilarity between genes, and lower distance indicates a higher probability of co-expression (Fig. 4C). With the Jaccard distance one module was identified for *Axin2*, *Tbx3*, *Lgr5*, *Itga6*, *Tert*, and *Notch2* (Fig. 4C and “Methods”). This module showed higher pair-wise similarity independently of gene expression levels. Additional genes showed higher distance and lower probability of co-expression*: Afp*, *Epcam*, *Sox9*, and *Icam1* (Fig. 4C and “Methods”). Furthermore, the percentage of nuclei expressing those gene markers was similar in both 2n and 4n hepatocytes (Supp. Fig. 4C) indicating that 2n and 4n hepatocytes have similar stem cell marker gene expression profiles. More than two stem/progenitor markers were co-expressed in both 2n and 4n hepatocytes (Fig. 4B, and Supp. Fig. 4D; “Methods”), strongly supporting the notion that polyploid hepatocytes have regenerative potential. Immunofluorescence (IF)/RNA-FISH co-detection analysis was used to simultaneously detect beta-catenin and *Lgr5* mRNA signals in the liver tissue (Supp. Fig. 7E). A higher percentage of *Lgr5* positive hepatocytes surrounding the central vein (CV) was detected compared to the periportal hepatocytes. These findings are in agreement with previous observations by Sun *et al*.^93^. Quantification of *Lgr5* mRNA copies per nucleus showed no significant differences in expression levels between 2n and 4n hepatocytes (Supp. Fig. 7E, right), thus validating our results generated from our snRNA-seq2 analyses.

In summary, no subpopulation of diploid hepatocytes enriched in stem/progenitor markers was detected in young healthy livers. Furthermore, polyploid hepatocytes co-express genes associated with proliferative and regenerative functions, and therefore have the potential to contribute to organ regeneration and liver homeostasis.

### Changes in expression pattern distribution in 4n nuclei indicate a high capacity for adaptation and regeneration

A focused analysis on energy homeostasis also revealed changes in the distribution pattern of key metabolic genes involved in lipid and glucose metabolism (Fig. 4D–G, Supp. Fig. 8 and Supp. Dataset 7). Some liver-specific transcription factors showed no change in mean expression or distribution pattern between 2n and 4n hepatocytes, for instance, *Hnf4a, Nr5a2* (LRH-1), *Nr1h4* (FXR), and *Rxa* (Fig. 4D). However, *Mlxipl* (ChREBP), *Nr3c1* (GR), *Nr1i2* (PXR), *Nr0b2* (SHP), and *Nr2f2* (COUPTF-II), and the coactivators *Ncoa1* (SRCa), *Crebbp* (CBP), and *Ppargc1c* (PGC1a) showed a different distribution pattern between 2n and 4n but no significant changes in mean expression. In some cases, such as *Nr1i3* (CAR), changes in distribution were associated with upregulation of its expression levels in 4n hepatocytes (Fig. 4D, Supp. Dataset 5 and Supp. Dataset 7). We further studied critical regulators of lipid metabolism and found that *Cpt1*, *Acox1*, *Apob,* and *Apoh* showed changes in distribution pattern but no significant changes in mean expression. Albeit, in *Acaca* (ACC) and *Alb*, we found that their distribution pattern changed and its expression was downregulated in 4n hepatocytes, while *Acox2* was upregulated in 4n hepatocytes.

For genes involved in glucose metabolism, statistically significant changes in the distribution pattern of *Gck*, *Gk*, *Slc2a9* (Glut9), *Insig1*, *Insig2*, *Scap*, and *Akr1d1* were found (Fig. 4F, Supp. Fig. 8 and Dataset 7). We observed considerable changes in the metabolism of xenobiotics and cytochrome P450 superfamily (Fig. 4G, Supp. Fig. 8 and Supp. Dataset 5). For instance, *Cyp4f14*, *Cyp2j5*, *Cyp2d9*, and *Cyp3a25* showed changes in gene expression distribution, while *Cyp2c70, Cyp7a1*, *Cyp1a2*, *Cyp2c50*, *Cyp2e1*, and *Cyp27a1* were additionally upregulated in 4n hepatocytes (Fig. 4G).

We further extended our analysis to hepatocytes with high levels of ploidy (>4n), and octaploid (8n), and decahexaploid (16n) nuclei were FACS sorted followed by snRNA-seq2 (Supp. Fig. 9 and Supp. Information). As expected, young mice had a low percentage of 8n (3%) and 16n (0.008%) hepatocytes in comparison to 2n (56%) and 4n (36%) hepatocytes (Supp. Fig. 9A, B). Similarly, we observed genes significantly upregulated or downregulated with high levels of ploidy, as well as statistically significant changes in gene expression distribution (Supp. Fig. 9C, D) again indicating high levels of cellular heterogeneity in the hepatocyte compartment.

In conclusion, rate-limiting enzymes involved in energy homeostasis and metabolism of drugs showed a statistically significant change in expression distribution between 2n and 4n hepatocytes. Changes in gene expression distribution have been associated with genetic plasticity and higher adaptation^101–104^, which could be important when the liver is faced with a chronic or overwhelming insult.

### Hepatic metabolic zonation determines gene expression levels independently of the ploidy status

**Fig. 5:**
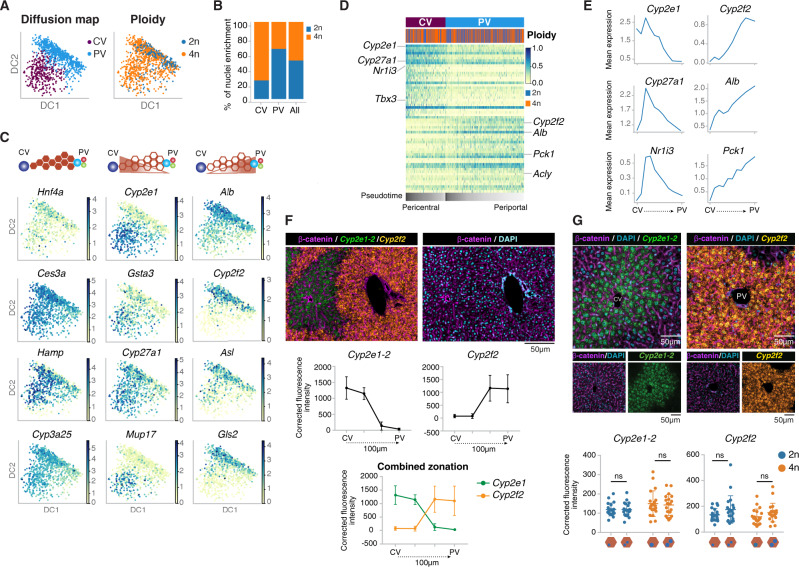
Pseudospatial ordering of hepatocytes along the liver lobe shows functional crosstalk between ploidy and zonation. **A** Diffusion map based on zonation markers, colored by annotated zones (left) and ploidy (right). **B** Bar plot showing a higher percentage of nuclei in the pericentral cluster in comparison to the periportal cluster, colored by ploidy. **C** Diffusion maps colored by the expression of zonation marker genes, for representative non-zonated genes (*Hnf4a*, *Ces3a*, *Hamp*, and *Cyp3a25*), pericentral genes (*Cyp2e1*, *Gsta3*, *Cyp27a1*, and *Mup17*), and periportal genes (*Alb*, *Cyp2f2*, *Asl*, and *Gls2*). **D** PAGA path heatmap showing the top 30 differentially expressed genes per zone between the periportal (PV) and pericentral (CV) clusters of nuclei, where 4n hepatocytes are enriched in the CV zone (colored by log-transformed, normalized counts). **E** Line plots depicting the mean expression of representative zonation markers along the diffusion pseudospace vector ordered from CV to PV within a liver lobule. **F** Representative image of IF/RNA-FISH co-detection analysis, showing expression levels of *Cyp2e1-*2 (green) and *Cyp2f2* (yellow) mRNA in a liver lobule (*n* = 3). The fluorescence intensity of each gene was calculated and normalized against background intensity. The corrected fluorescence intensity is plotted against a linear distance (100 µm) from CV to PV. **G** Corrected fluorescence intensity of *Cyp2e1-2* and *Cyp2f2* signals were categorized according to 2n or 4n mononucleated or binucleated hepatocytes, determined by b-catenin (magenta) and 4′,6-diamidin-2-phenylindol (DAPI; cyan) staining. No significant changes were detected between ploidy levels or the number of nuclei from an unpaired *t*-test analysis (ns not significant). **F**, **G** Data points are expressed as mean ± s.d.

Zonation of hepatocytes and endothelial cells along the portal-central axis of the liver lobule has previously been investigated^11,12^. Recently, 2n and 4n hepatocytes have been localized to specific metabolic zones (periportal or pericentral) or randomly interspersed throughout the hepatic lobe^97,105,106^. We used diffusion pseudotime (dpt), visualized in diffusion maps, to infer the pseudospatial ordering of nuclei according to defined markers associated with liver zonation and their metabolic specialization^12,107,108^. *Louvain* clustering on these zonation markers established three clusters (Fig. Supp. 10A and Supp. Information). After visual investigation of pericentral and periportal marker genes on the diffusion map, two clusters (0 and 2) were assigned to a periportal cluster, and cluster 1 to a pericentral cluster (Fig. 5A, Supp. Fig. 10A, and Supp. Information). Then, the percentage of 2n and 4n hepatocytes in the pericentral (CV) and periportal (PV) clusters were calculated respectively (Fig. 5B and Supp. Information)^12^. We observed a 1.3-fold relative enrichment of 4n hepatocytes in the pericentral cluster, indicating that they reside close to the central vein. This is in agreement with recent studies in which polyploid hepatocytes have been shown by histological analysis to be enriched in the pericentral zone, and diploid hepatocytes in the periportal zone using cell-lineage tracing^79,109^.

We further studied the expression levels of classic markers of liver zonation such as *Cyp2e1*, *Gsta3*, *Cyp27a1,* and *Mup17* for the central vein, and *Alb*, *Cyp2f2*, *Asl*, and *Gls2* for the portal vein (Fig. 5C; Supp. Fig. 10). Non-zonated markers were also selected, for instance, *Hnf4a* whose mRNA expression is non-zonated as opposed to its protein levels^10^. Additional non-zonated genes including *Ces3a*, *Hamp*, and *Cyp3a25* were also studied (Fig. 5C, Supp. Fig. 10,). In order to verify the spatial distribution of 4n hepatocytes, we generated a partition-based graph abstraction (PAGA) to visualize coordinated changes in expression across the pseudospace in the hepatic lobule^110^ (Supp. Information). The thirty most differentially expressed genes in each of the two zonation clusters were selected along the diffusion pseudotime, again showing an enrichment of 4n hepatocytes in the pericentral cluster and the expected changes of pericentral and periportal markers, respectively (Fig. 5D, E, Supp. Fig. 11 and Supp. Dataset 8). Out of 224 zonation markers that were upregulated in the pericentral cluster, 55 were upregulated only in 4n hepatocytes. Meanwhile, out of 68 zonation markers that were upregulated in the periportal cluster 12 were upregulated in 2n hepatocytes. We binned the vector of diffusion pseudotime into 10 bins and visualized the mean expression of specific zonation markers along these bins (Fig. 5E, Supp. Fig. 11). This approach is suitable to visualize simultaneously the decrease and increase in mean expression of periportal and pericentral genes along these bins. These results showed that liver zonation can be investigated by diffusion pseudotime using our snRNA-seq2 approach. In particular, hepatic metabolic zonation was found in both 2n and 4n hepatocytes indicating that spatial zonation determines gene expression levels independently of the ploidy status.

In order to validate the spatial relationship shown by changes in the nuclear transcriptome, a thorough analysis of cell size and ploidy was done based on nuclear content using ImageStream Mk II (Supp. Fig. 9E–H). Additional immunofluorescence staining for Lyve1, a marker of endothelial cells indicated that their nuclei are smaller in size (3–6 µm diameter) and represent the smaller peak within the 2n population (Supp. Fig. 9E–H). After analysis of the nuclei size (µm^2^) and diameter (µm) of 2n, 4n, and 8n hepatocytes, DNA-FISH with a custom-designed probe against *Hnf4a* gene in chromosome 2 was used to confidently identify nuclei with 2, 4, and 8 copies of *Hnf4a* gene locus (Supp. Fig. 12A–E). Additional immunostainings with DAPI and beta-catenin, to annotate nuclei and the cytoplasmic membrane respectively, allowed tissue mapping and quantification of diploid (2n), tetraploid mononucleated (4n) and binucleated (2×2n), and octaploid binucleated (2×4n) cells (Fig. 12F, G). This approach allowed us to confidently identify diploid and tetraploid cells (mono- and binucleated), verify the predictions of the diffusion maps, and validate our findings using snRNA-seq2 in which 4n nuclei are enriched in the pericentral vein area (Fig. 5B).

Importantly, IF/RNA-FISH co-detection was used to quantify the number of *Cyp2e1* and *Cyp2f2* mRNA molecules in four zones within the liver lobule (from central to portal vein) (Fig. 5F, G). We observed that the mRNA level of *Cyp2e1* decreases towards the periportal area, whereas *Cyp2f2* mRNA levels increase (Fig. 5F). These results in tissue sections validated our findings using snRNA-seq2 and computational approaches (Fig. 5E). The combination of RNAscope staining for *Cyp2e1* and *Cyp2f2* mRNAs with DAPI and beta-catenin allowed accurate assessment of mRNA levels in polyploid hepatocytes. We found no significant differences in the mRNA levels between 2n nuclei in diploid and tetraploid binucleated cells using RNAscope (Fig. 5G), and those populations cannot be distinguished by snRNA-seq2 (Fig. 3 and Supp. Fig. 6B).

In summary, our results indicate a tight coordination between gene expression in polyploid hepatocytes and their spatial location within the liver lobule. Therefore, hepatocytes adjust their gene expression according to their spatial distribution, demonstrating a functional crosstalk between liver zonation and ploidy. We then further demonstrated that zonation is the major determinant of gene expression levels in polyploid hepatocytes by using a mouse model of centrilobular injury-induced liver fibrosis (CCl_4_-induced liver fibrosis)^14,111^. We found that the relative percentages of 2n and 4n hepatocytes changed following chronic liver injury, with an increased number of 4n hepatocytes in the pericentral area following pseudotime ordering, an observation that was supported by FACS analysis (Supp. Fig. 13A–F). Furthermore, the diffusion maps, PAGA representation, and histological analysis clearly showed an alteration in the zonation pattern in the CCl_4_ treated mice, with profound changes observed in the pericentral hepatocyte compartment (Supp. Fig. 13A, B, Supp. Fig. 14A, B, and Supp. Dataset 9). Despite the fact that zonation was markedly affected by CCl_4_ treatment, 2n and 4n hepatocytes coordinate their gene expression levels according to their spatial distribution in the liver lobule. More precisely, 4n hepatocytes are enriched in the peri-central vein area, suggesting that polyploid hepatocytes undergo genetic plasticity and higher adaptation to deal with iterative liver injury^39,43,112^.

Taken together, our data demonstrate that hepatocyte ploidy and zonation are two coordinated but independent processes in which polyploid hepatocytes adjust their gene expression level according to their position within the liver lobule.

## Discussion

Single-cell genomics allows the unbiased exploration of cell states and cell types at single-cell resolution, leading to a revolutionary change in our understanding of liver biology and disease pathogenesis^16^. However, single-cell RNA-seq (scRNA-seq) in the liver is associated with some caveats. First, dissociation protocols including enzymatic or mechanical dissociation lead to changes in the cellular transcriptome^17–19^. Second, dissociation may lead to the underrepresentation of certain cell types due to cell fragility or large cell-size, such as hepatocytes^113^. Third, it relies on the isolation of intact cells from fresh tissues, which hinders its clinical application on human samples. In this context, single-nucleus RNA-seq has emerged as a complementary approach that relies on the unbiased assessment of nuclei from all cells present in a tissue^20,21,37^. The analysis of the nuclear transcriptome has proven to be very powerful in the study of cell-type diversity in the mouse and human tissues, including brain^21,23–27,114^, spinal cord^53^; breast cancer^54^, kidney^29–32^, lung^28^, heart^34,35^, and a variety of human tumor samples^37^.

We have developed a single-nucleus RNA-seq2 method that improves the lysis of the nuclear membrane and significantly increases the number of transcripts detected per nuclei, comparing favorably with transcript data derived from whole-cell scRNAseq (Fig. 1 and Supp. Fig. 1). Our method is highly sensitive, reproducible and it has been specifically designed for frozen, archived liver samples (Fig. 1). The unbiased sorting of single-nuclei allows the study of all the main cell types within the liver, and this approach can be used to interrogate cellular crosstalk and transcription factor networks (Figs. 1 and 2). Indeed, snRNA-seq2 has the potential for comprehensive analysis of fresh and archived flash-frozen liver samples from both mice and humans.

Notably, scRNA-seq has revealed the high degree of functional specialization of multiple cell types within the liver lobule, for instance for hepatocytes, endothelial cells^9,11,12,52^, and macrophages^14,15,73^. Additionally, it has been shown how changes in mRNA correlate closely with changes in protein levels in the liver^10^. However, little is known about the functional role of polyploid hepatocytes at the single-cell level. Recently, Katsuda *et al*. have shown in rats how genes associated with hepatic zones have differential expression patterns in polyploid hepatocytes^78^. Here, we present a genome-wide analysis of the nuclear transcriptome of individual 2n and 4n nuclei in mice (Figs. 3–5). Our study is focused on the analysis of ploidy based on the DNA content of each nucleus (2n and 4n), and we cannot resolve whether 2n nuclei belong to either diploid cells or binucleated tetraploid cells (2nx2)^41,82^. Moreover, it has been shown that hepatocyte volume does not depend on the number of nuclei but rather on their ploidy status, whereas mono-nucleated hepatocytes (2n) and bi-nucleated hepatocytes (2nx2) have the same cellular volume^8,115^. In young wild-type mice, an incomplete cytokinesis leads to tetraploid cells (2n×2) that are generally associated with the fidelity of chromosome transmission^41,82^. For these reasons, we have focused our analysis on the 2n and 4n nuclei, for which we do not expect chromosomal abnormalities^116,117^.

Additionally, cell size is the major determinant in biochemical reaction rates^118,119^ and cell size increases with ploidy. It has been reported that transcript abundance correlates with cellular volume at the single-cell level due to an increase in global transcription in larger cells^89^. During partial hepatectomy, hepatocytes increase cell size before proliferation^120^. In particular, in the liver-specific *Cdk1* knockout mice, larger hepatocytes displayed alterations in lipid and mitochondrial metabolism^121^, suggesting that polyploid hepatocytes could have a more prominent role in energy homeostasis compared with diploid hepatocytes.

Our transcriptomic analyses revealed that 2n and 4n hepatocytes are globally very similar, but 312 genes are found to be differentially expressed between these two groups (Fig. 3). This suggests that if polyploid hepatocytes increase during pathological processes, they could lead to gene expression imbalances of functionally relevant genes (Figs. 3 and 4). Additionally, the transcriptional variability in young mice is also lower in 4n hepatocytes, which has previously been shown using single-molecule fluorescence in situ hybridization (smFISH) for the beta-actin (*Actb*) gene^122^. Whether transcriptional variability changes during ageing^47^ or in chronic liver disease still remains to be investigated. Interestingly, the presence of mononucleated tetraploid hepatocytes has been associated with human hepatocellular carcinoma (HCC)^40,86,87,123^. Likewise, the number of polyploid hepatocytes also increases in models of NAFLD, including ob/ob mice and wild-type mice fed with methionine-choline-deficient diet (MCD) or high-fat diet (HFD)^86,124^. Therefore, alterations in the number of 2n and 4n hepatocytes are observed during NAFLD in both animal models and patients. The transcriptomic analysis of 2n and 4n hepatocytes in archived, frozen samples could open an entirely new strategy to understand disease pathogenesis and search for new therapies to treat liver disease.

The liver is characterized by its regenerative potential. However, the cellular origin that triggers liver homeostasis and repair is still under debate. Recently, complementary studies using different cell lineage-tracing models have shown that all hepatocytes have comparable self-renewal potential^79,93,98^. Our transcriptomic analysis also supports the notion that the vast majority of hepatocytes, regardless of their ploidy level and location within the hepatic lobule, co-expresses stem/progenitor gene markers and have the potential to contribute to liver homeostasis after hepatic injury (Fig. 4B, C).

Furthermore, the broad spatial heterogeneity in the liver shows that key liver functions are zonated^9^, and recently it has been shown that the zonation is perturbed during liver fibrosis^14,15^. In this context, the spatial distribution of polyploid hepatocytes and their functional consequences are still debated^8,41,79,87,109^. We used diffusion pseudotime to infer the pseudospatial ordering of 2n and 4n hepatocytes according to defined markers of liver zonation, and we found that 4n nuclei are distributed throughout the hepatic lobe with an enrichment in the pericentral zone (Fig. 5). These results support previous studies in which polyploid hepatocytes were quantified by lineage-tracing^79^ or smFISH for the glutamine synthetase (*Glu1*) gene^109^. Additionally, we have shown that 2n and 4n hepatocytes adjust their gene expression levels in a coordinated manner depending on their spatial location within the liver lobule. In summary, we have found that 4n hepatocytes are enriched 1.3-fold in the pericentral zone, and that hepatocyte ploidy and liver zonation are tightly regulated. Altogether, we have discovered that the division of labor in hepatocytes is linked to both their spatial distribution and ploidy levels, and this crosstalk could be affected during ageing and chronic liver disease.

In summary, our methodology has the potential to investigate previously archived frozen murine and human samples, and interrogate how changes in gene expression correlate with the development and progression of liver disease, taking into account cellular crosstalk and signaling pathways. Our analysis shows that hepatocytes in young livers, without injury or selective pressure, express stem cell markers across the portal-central axis independently of their ploidy status. These findings support recent reports showing that polyploid hepatocytes are proliferative and contribute to liver regeneration. Moreover, hepatocytes with different levels of ploidy adjust their gene dosage according to their position in the liver, showing a functional crosstalk between liver zonation and ploidy. We anticipate that changes in the number of 2n and 4n hepatocytes in the overall liver cell composition will lead either too protective or deleterious outcomes during ageing and chronic liver diseases.

## Methods

### Ethics statement

This investigation was approved by the Animal Welfare and Ethics Review Board and followed the Cambridge Institute guidelines for the use of animals in experimental studies under Home Office licences PPL 70/7535 until February 2018 and PPL P9855D13B from March 2018. All animal experimentation was carried out in accordance with the Animals (Scientific Procedures) Act 1986 (United Kingdom) and conformed to the Animal Research: Reporting of In Vivo Experiments (ARRIVE) guidelines developed by the National Center for the Replacement, Refinement, and Reduction of Animals in research (NC3Rs).

### Mice and liver tissue collection

All wild-type C57BL/6 mice were purchased from Charles River UK Ltd (Margate, United Kingdom) and were maintained under specific pathogen-free conditions at the University of Cambridge, CRUK—Cambridge Institute under the auspices of a UK Home Office license. These animal facilities are approved by and registered with the UK Home Office. All mice were maintained in a specific pathogen-free environment in Individually Ventilated Caging units. Mice were kept in a positive pressure system, maintaining a temperature between 19 and 23°, 55% humidity (± 10%), 20 total air changes per hour, and under a 12 h light/dark cycle. Mice had free access to a standard laboratory diet (PicoLab Mouse Diet 20, 5R58) and water. All male C57BL/6 animals were sacrificed by an approved scientist in accordance with Schedule 1 of the Animals (Scientific Procedures) Act 1986. Young 3-month-old male mice were sacrificed and individual pieces of the harvested liver were immediately flash-frozen. A small piece of liver tissue was collected in 4% paraformaldehyde. All young animals for this study were macroscopically inspected for any signs of pathologies or abnormalities.

### Liver fibrosis model

Carbon tetrachloride (CCl_4_)-induced liver fibrosis was induced as described previously^111^. Mice were injected i.p. with 1 mL/g body weight CCl_4_ in a 1:3 ratio with olive oil (0.25 µL/g CCl_4_) twice weekly for 6 weeks; livers were harvested 48 h after the final injection^14^.

### Single nuclei isolation/homogenization

For the single nuclei isolation, the protocol described by Krishnaswami et al.^21^ was followed with several modifications. In summary, fresh frozen liver tissues (~3 mm^3^) were homogenized using a 2 mL Dounce homogenizer (Lab Logistics, #9651632) in 1 mL of ice-cold homogenization buffer, HB (250 mM sucrose, 25 mM KCl, 5 mM MgCl_2_, 10 mM Tris buffer, 1 μM DTT), 1 x protease inhibitor tablet (Sigma Aldrich Chemie), 0.4 U/μL RNaseIn (Thermo Fisher Scientific), 0.2 U/μL Superasin (Thermo Fisher Scientific), 0.1% Triton X-100 (v/v) and 10 µg/mL Hoechst 33342 (Thermo Fisher Scientific) in RNase-free water.

Before the dounce homogenization, the frozen tissue was placed in a pre-chilled petri dish on ice containing 1 mL of HB and finely cut with a pre-chilled scalpel blade until all the pieces can be transferred to the homogenizer using a wide orifice 1 mL tip. Throughout the procedure, all pipetting steps of transferring the samples were made using wide orifice tips (Rainin, #17014297) to minimize shear force.

Always on ice, we performed five very slow strokes with the loose inner tolerance pestle and ten more strokes using the tight inner tolerance pestle. Collecting 10 µL of the solution, the nuclei suspension was inspected under a microscope on a Neubauer chamber with 10 µL of Trypan blue (0,4%). If aggregates were predominant, up to five more strokes were performed using the tight pestle. The suspension was passed through a 50 μm sterilized filter (CellTrics, Symtex, #04-004-2327) into a 5 mL Eppendorf pre-chilled tube washing the Dounce homogenizer with additional 500 μL of cold homogenization buffer. Subsequently, the suspension was centrifuged at 1000 × *g* for 8 min in an Eppendorf centrifuge at 4 °C (5430R). The pellet was then resuspended in 250 μL of pre-chilled homogenization buffer. To ensure high quality of single-nuclei suspensions, we performed a density gradient centrifugation clean-up for 20 min, using Iodixanol gradient (Optiprep, D1556, Sigma Aldrich Chemie). The final pellet was resuspended gently in 200 µL of nuclei storage buffer (NSB) (166.5 mM sucrose, 5 mM MgCl_2_, 10 mM Tris buffer pH 8.0) containing additional RNase inhibitors 0.2 U/μL Superasin (Thermo Fisher Scientific, #AM2696) and 0.4 U/μL Recombinant RNase Inhibitor (Takara Clontech #2313A). A final visual inspection and counting were performed under a microscope and the single nuclei suspension was filtered through a 35 μm cell strainer cap into a pre-chilled FACS tube prior FACS sorting.

### Flow cytometry

Hoechst dye (Life technologies, #H3570) was used to stain all nuclei during nuclei isolation, allowing to distinguish between diploid and tetraploid nuclei using a FACS sorter (BD FACSAria Fusion 1 and/or BD FACSAria 3) with a 100 μm nozzle. Before sorting, 384-well thin-walled PCR plates (BioRad, #HSP3901) were freshly prepared with 940nL of reaction buffer (following the manufacturer’s instructions, only 1 μL of 10X reaction buffer is diluted in 2,75 μl of water). Herein, this reaction buffer will be termed Lysis Buffer 1—LB1) (Takara kit SMART-Seq v4 Ultra Low input RNA) using a liquid miniaturization robot (Mosquito HV, STP Labtech) and kept on ice. Reaction buffer was prepared following the manufacturer’s instruction adding 1 μL of RNAse Inhibitor in 19 μL of 10X Lysis Buffer. The FACS droplet delay and cut-off point were optimized prior to every sorting, the plate holder was cooled at 4 °C and all settings and calibrations were done by the FACS operator while samples were processed to avoid additional delays that could lead to RNA degradation of the samples.

In brief, the gating strategy used FSC-A/SSC-A to select intact nuclei stained by Hoechst, in which events above 10^4 are considered Hoechst positive. Subsequently, selection of singlets was performed by FSC-A/FSC-H followed by exclusion of doublets by FSC-A/FSC-W. Identification of different levels of ploidy was performed by FSC-A/Hoescht and FSC-A/Modal as previously described^39^ (Supp. Fig. 13).

The sorting accuracy in the 384 well-plates was assessed using a colorimetric method with tetramethyl benzidine substrate (TMB, BioLegend, #421501) and 50 μg/mL of Horseradish Peroxidase (HRP, Life Technologies, #31490)^125^. Only when the plate alignment testing results to single events to more than 95% of the wells, we proceed with the fresh isolated nuclei. The nuclei suspended in NSB was diluted at ~10 × 10^4^ nuclei per mL and kept on average 200−1000 events per second as flow rate. In the sorting layout, we sorted diploid (2n) cells in half of the 384 well plate and tetraploid (4n) in the remaining half for each individual. After sorting, every plate was sealed (MicroAmp Thermo Seal lid, #AB0558), shortly vortexed (10 s), centrifuged (prechilled at 4 °C, 2000 x *g* for 1 min), frozen on dry ice, and stored at −80 °C, until cDNA synthesis.

### snRNAseq-2

For the generation of double-stranded full-length cDNA, we used the Smart-Seq2 chemistry from a commercial kit (SMART-Seq v4 Ultra Low input RNA, Takara) and we optimized the lysis of the nuclear membrane by adding a supplementary lysis buffer (Lysis Buffer 2, LB2). Firstly, using a low volume liquid handling robot, we miniaturized the volumes reducing the amount four-times and keeping the same ratios for all the reagents, significantly lowering the experimental cost. Liquid handling robots allowed to increase pipetting accuracy, maintained a sterile and temperature-controlled environment, and reduced the user variability and potential cross-contamination. Our additional Lysis Buffer 2 (LB2) consist in a mixture of 0.4 % NP-40 (v/v) final concentration (Life Tech, #85124) and 0.1% Triton-X100 (v/v) final concentration (Fisher, #10671652). Using the Mosquito HV (Labtech STP), we added 2190nL of our additional snRNAseq-2 lysis mix in each well together with the first strand synthesis primers and the spike-in controls (ERCC). Ratios to the final volume (3.125 μL) of the total Lysis Buffer (1 and 2) are shown in parentheses (NP40 2% (2.5/12.5, Triton-X100 1% (1.25/12.5), ERCC spike-–in at 1/300.000 dilution (1/12.5) and 3′ SMART-seq CDS Primer II A (2/12.5) and additional water (2/12.5). Every plate was thawed directly on a −20 °C chilled holder while LB2 was added by the Mosquito HV robot. Then, the plate was sealed, vortexed vigorously (20 s in a Mixmate (Eppendorf) 2000 rpm), centrifuged (30 s in a pre-chilled Eppendorf 5430R at 4 °C, 2000 × *g*), and placed in a Thermal cycler (BioRad C1000) for 6 min at 72 °C). We suggest to use the same lot (e.g., #00769049) of ERCCs (Life Technologies, #4456740) per project. ERCC spike-ins were diluted 1 in 10, in water with 0.4 U/μL Recombinant RNase Inhibitor (Takara Clontech #2313A), aliquoted, and stored at −80 °C. Fresh dilution of 1 in 300 000 and 1 in 100 000 were prepared immediately before the first strand synthesis.

Next, reverse transcription and Pre-PCR amplification steps were followed as described by the manufacturer. We maintained our four-times reduced volumes for all steps. As a final modification on the Takara kit protocol, we optimized the PCR cycles program for the cDNA amplification. We used 21 cycles and a PCR programs consisting of: 1 min at 95 °C, [20 s at 95 °C, 4 min at 58 °C, 6 min at 68 °C] × 5, [20 s at 95 °C, 30 s at 64 °C, 6 min at 68 °C] × 9, [30 s at 95 °C, 30 s at 64 °C, 7 min at 68 °C] × 7, 10 min at 72 °C.

Internal ERCC spike-ins were used as positive controls and the cDNA yield was assessed in an Agilent Bioanalyzer with a High Sensitivity DNA kit. In our protocol, we did not perform a bead clean-up before final library preparation. The miniaturization details and Mosquito programs used can be found in the supplementary information. RNA-seq library preparation and sequencing are described in the supplementary information.

### RNA-seq library preparation and sequencing

Sequencing libraries were prepared using the standard Illumina Nextera XT. DNA Sample Preparation kit (Illumina, #FC-131-1096) and the combination of 384 Combinatorial Dual Indexes (Illumina- Set A to D, #FC-131-2001 to FC-131-2004). Using the Mosquito liquid handling robot, the Nextera XT chemistry was miniaturized^126,127^ (Supp. Dataset 10–14 and Supp. Information). All the final 384-pooled libraries were sequenced using Illumina HiSeq4000 NGS sequencer in a paired-end—150 bases length.

### Fluorescence in situ hybridization

Detection of mouse *Hnf4α* was performed on FFPE sections. Briefly, sections were cut at 3 µm thick, baked for 1 h at 60 °C before deparaffinization in xylene and rehydration through graded ethanol. Pretreatments were carried out using Kreatech Tissue Digestion Kit II reagents (Cat # KBI-60004, Leica Biosystems) according to manufacturer’s instructions: using 0.2 M HCl for 30 min at room temperature, followed by 3 min wash in milliQ water, 30 min incubation in Pretreatment solution B at 80 °C, 3 min wash in 2X SSC, 30 min in pepsin solution at room temperature, 1 min in milliQ water and 5 min wash in 2X SSC. Slides were dehydrated through graded ethanol. Custom design HNF4 probe (Cat # HNF4A-20-OR, Empire Genomics) was applied to each slide and coverslips were sealed with Fixogum rubber cement (Cat # ICNA11FIXO0125, VWR). Slides were denatured for 5 min at 80 °C before hybridization overnight in a humid chamber at 37 °C. Coverslips were carefully removed and slides were washed in Buffer II (Kreatech Tissue Digestion Kit II, KBI6004) at room temperature for 2 min, followed by Buffer I at 72 °C for 2 min and then Buffer II again at room temperature for 2 min. The slides were dehydrated through graded ethanol prior to incubation with Sudan Black B (Cat # 199664, Sigma Aldrich, 0.5% in 70% ethanol, stirred for 2 h in the dark, filtered with 0.22 um filter) for 10 min. The slides were washed for 5 min each in three changes of PBS prior to mounting with Prolong Gold Antifade Mountant with DAPI (ThermoFisher Scientific, Cat No. P36931, 1:1000). Additional immunofluorescence and image analysis are described in the supplementary information.

### Multiplexed RNAscope and immunofluorescence co-detection

Simultaneous detection of mRNA for mouse *Cyp2e1-2* and *Cyp2f2*, or *Lgr5* and mouse beta-catenin protein was performed on FFPE sections using Advanced Cell Diagnostics (ACD) RNAscope® 2.5 LS Multiplex Reagent Kit (Cat No. 322800), RNAscope® 2.5 LS Probe Mm-Cyp2f2 (Cat No. 451858), RNAscope® 2.5 LS Probe Mm-Cyp2e1-C2 (Cat No. 402788 -C2), or RNAscope® 2.5 LS Probe Mm-Lgr5 (Cat No. 312178) in combination with ACD Co-Detection Antibody Diluent (Cat No. 323160) (ACD, Hayward, CA, USA), and a mouse monoclonal beta-catenin antibody from BD Biosciences (Cat No. 610154). Briefly, sections were cut at 3 µm thickness, baked for 1 h at 60 °C before loading onto a Bond RX instrument (Leica Biosystems). Slides were deparaffinized and rehydrated on-board prior to heat pre-treatment with Epitope Retrieval Solution 2 (Cat No. AR9640, Leica Biosystems) at 95 °C for 30 min. The antibody was diluted to 1:500 using the ACD Co-Detection Antibody diluent and incubated for 15 min at ambient temperature, before post fixation with 10% neutral buffered formalin (NBF) for 30 min and protein digestion using ACD Enzyme from the Multiplex Reagent kit at 40 °C for 30 min. Probe hybridization and signal amplification were performed according to the manufacturer’s instructions. TSA plus-Cy5 (Akoya Biosciences Cat No. NEL745001KT) detection at 1:500 dilution of beta-catenin protein, TSA plus-Cy3 (Akoya Biosciences Cat No. NEL744001KT) detection at 1:500 dilution of *Cyp2e1-2* or *Lgr5*, and TSA plus—Fluorescein detection at 1:500 dilution of *Cyp2f2* (Akoya Biosciences Cat No. NEL741001KT) were performed on the Bond Rx according to the ACD protocol. Slides were then removed from the Bond Rx and mounted using Prolong Diamond (ThermoFisher Cat No P36965). The slides were imaged on the AxioScan (Zeiss) to create whole slide images. Images were captured at 40× magnification, with a resolution of 0.25 microns per pixel. Image analyses were performed using the HALO (Indicalabs) software.

### Read alignment and pre-processing

Raw sequencing reads were mapped against a customized genome containing both mm10 (GRCm38, assembly version 93), and the ERCC92 sequences^128^. Mapping was done using STAR-2.7.1a with the following parameters --outFilterMultimapNmax 1 --outSAMtype BAM SortedByCoordinate. Potential PCR-duplicates were identified and removed using MarkDuplicates from picard tools version 2.20.2 with the option REMOVE_DUPLICATES=true. For every single nucleus, reads mapping to individual transcripts were counted and summed per gene using htseq-count version 0.11.3 with the following parameters: -m intersection-nonempty -f bam -r pos -s no --nonunique all -t transcript -i gene_id --additional-attr=gene_name.

The resulting raw count matrix was loaded into python and stored as an AnnData object, anndata version 0.7.1. The downstream analysis described below was adapted from Luecken et al.^5,5^. Unless described otherwise, functions implemented in scanpy (version 1.4.5.2.dev6+gfa408dc7) were used in downstream analysis^110^. Additionally, to the count matrix based on full transcripts, count matrices for exonic and intronic reads, respectively were built using featureCounts^129^.

### Quality control and filtering

Starting with a matrix of 2496 single nuclei and 54 329 genes, nuclei were kept if the percentage of mapped reads corresponding to ERCC spike-in transcripts was higher than 5% but lower than 90%, and if nuclei had >1000 and <7000 genes detected. Then, genes sequenced in fewer than 25 cells (about 1% of the population) and with read count below 250 reads were filtered out. Finally, only the nuclei having less than 7000 genes detected, and a library size of 10 000 to 300 000 reads were kept. This processing yielded a matrix of 2016 single nuclei times 19 340 genes.

### ERCC size factor calculation and normalization

Two different ERCC dilutions were used (1:100 000 and 1:300 000 respectively). When sequencing to saturation, the proportional amounts of sequenced endogenous transcripts and synthetic spike-ins depend on the input number of endogenous transcripts. The first step in this normalization approach is to calculate ERCC size factors as the sum of ERCC reads per nucleus divided by the mean ERCC reads across all nuclei within one dilution. Subsequently, to normalize the expression matrix, the read counts were first divided by the corresponding gene length in kilobases. Then, the cell coverage was corrected by the following approach: endogenous counts were summed per nucleus, and the sum was divided by 10 000 times the ERCC size factor, yielding the normalization factor per nucleus. Finally, the endogenous reads per nucleus were divided by this factor. Thereby, reads stemming from nuclei with few endogenous reads and many ERCC reads are divided by a smaller factor than reads stemming from nuclei with many endogenous reads and proportionally few ERCC reads.

1 $${x}_{{ij}}^{{\prime} }=\frac{\frac{{x}_{{ij}}}{{L}_{j}}}{\frac{{\sum }_{j}^{N}\frac{{x}_{{ij}}}{{L}_{j}}}{10000\cdot {\mathrm{s}}{\mathrm{f}}_{i}}}$$

where *x*′_*ij*_ is the normalized count of gene *j* in cell *i*, *L*_*j*_ is the length of gene *j* and sf_*i*_ is the ERCC size factor of cell *i*.

After normalization, cells with more than 50 000 gene length-normalized total counts were removed. Technical replicates SNI-234(R2) and SNI-235(R2) that contain the same cells as SNI-160 and SNI-116, respectively, were not further considered (Supp. Dataset 1). This resulted in a final matrix of 1649 nuclei times 19 258 genes. The normalized counts were log-transformed by applying a natural logarithm to one plus the values in the count matrix. Batch effects were corrected for using *combat* with plates as a covariate^130^. Description of clustering and visualization are further described in the supplementary information.

### Differential expression analysis

In order to find differential expressed genes between groups of interest, Welch’s t-tests were done as implemented in the scanpy function rank_genes_groups with the parameters method=”t-test” and n_genes=19258. First, this was done between cell types, considering 2n and 4n hepatocytes together as one group, and then, only hepatocytes were taken in order to compare 2n to 4n. Genes that had a log2 fold change of greater than 0.5 and a Bonferroni-adjusted *p*-value below 0.05 were considered significantly upregulated; genes with a log2 fold change smaller than –0.5 and a Bonferroni-adjusted *p*-value below 0.05 were considered significantly downregulated.

To further investigate changes in the distribution of a given gene between 2n and 4n hepatocytes, a Kolmogorov−Smirnov test was performed. Genes were called as significantly changing their gene expression distribution between 2n and 4n if they had a *p*-value below 0.05 and a test statistic of >0.15.

For visualization purposes, 40 nuclei were randomly selected per cell type (for cell types with <40 nuclei, all nuclei were taken) and their respective top five differential expressed genes (based on their score calculated by rank_genes_groups) were taken to create a heatmap (Fig. 2A) using the ComplexHeatmap package in R. For visualization purposes, genes with a mean expression between 0.1 and 100 were selected in the MA plot (Fig. 3C).

### Co-expression analysis of liver stem cell markers

For this analysis, only hepatocytes were used. A heatmap of known hepatic stem cell markers showed expression of these markers not only in diploid but also in tetraploid nuclei. To investigate whether these markers are expressed in the same nuclei, the matrix was binarized after log-transformation and stored as an additional layer in the AnnData object. This binary matrix was a subset to only contain the stem cell markers. Nuclei not expressing any of the stem cell markers were removed, resulting in a matrix of 364 nuclei times 11 stem cell markers. Based on this, pairwise Jaccard distance between the stem cell markers was calculated.

2 $${\rm{J}}\left({\rm{X}},{\rm{Y}}\right)=\frac{\left|{\rm{X}}\cap {\rm{Y}}\right|}{\left|{\rm{X}}\cup {\rm{Y}}\right|}$$

where X is the binary expression vector of gene *j*_1_ across the nuclei, and Y is the binary expression vector of gene *j*_2_ across the nuclei.

Furthermore, pairwise Jaccard distances between nuclei and genes, respectively, were used to calculate linkage for hierarchical clustering. By this approach, the gene’s expression level per nucleus is neglected and only its presence or absence is counted. Additional downstream analyses are described in the supplementary information.

### Reporting summary

Further information on research design is available in the Nature Research Reporting Summary linked to this article.

## Supplementary information

Supplementary Information

Description of Additional Supplementary Files

Dataset 1

Dataset 2

Dataset 3

Dataset 4

Dataset 5

Dataset 6

Dataset 7

Dataset 8

Dataset 9

Dataset 10

Dataset 11

Dataset 12

Dataset 13

Dataset 14

Reporting Summary

## Data Availability

All raw sequencing data is deposited and publicly available in ArrayExpress under accession numbers “E-MTAB-9333”^131^ and “E-MTAB-10223”^132^. Additional publicly available data used for method comparison was obtained from either GEO (Accession numbers “GSE84498”: GSE84498_umitab.txt.gz^12^, “GSE124395”: GSE124395_Normalhumanlivercellatlasdata.txt.gz^52^, “GSE148339”: GSE148339_liver.integrated.final.RData.gz^33^ or from 10.6084/m9.figshare.5829687.v7 and 10.6084/m9.figshare.5968960.v2^51^). All other relevant data supporting the key findings of this study are available within the article and its Supplementary Information files or from the corresponding author upon reasonable request. Source data are provided with this paper. A reporting summary for this Article is available as a Supplementary Information file. Source data are provided with this paper.

## Source data

Source Data

## Code availability

The scripts (jupyter notebook and R) containing code to reproduce the analysis are publicly available at http://github.com/celiamtnez/snRNA-seq2_young^133^. Packages used for the analysis include ComplexHeatmap (v2.6.2)^131,134^, matplotlib (v3.2.1)^132,135^, pandas (v1.0.3)^133,136^, numpy (v1.18.1)^14^, seaborn (v0.10.0).^137^, and scipy (v1.4.1)^138^. The version of the code at the time of publication is stored in Zenodo: 10.5281/zenodo.4694749^133^.
