# Supplementary material for: Single-nucleus RNA-seq2 reveals functional crosstalk between liver zonation and ploidy
Source: Nat Commun. 2021 Jul 12;12:4264. doi: 10.1038/s41467-021-24543-5 (PMC8275628; doi:10.1038/s41467-021-24543-5)
Supplement: Supplementary file 1 — Supplementary Information [file 41467_2021_24543_MOESM1_ESM.pdf]

SUPPLEMENTARY INFORMATION

Single-nucleus RNA-seq2 reveals functional crosstalk between liver zonation and ploidy

M. L. Richter[1†], I.K. Deligiannis[1†], K. Yin[1,2], A. Danese[3], E. Lleshi[2], P. Coupland[2], C.A. Vallejos[4], K.P. Matchett[5], N.C. Henderson[4,5], M. Colome-Tatche[3,6,7*] and C.P. Martinez-Jimenez[1,8*]

[1] Helmholtz Pioneer Campus (HPC), Helmholtz Zentrum München, 85764 Neuherberg, Germany
[2] University of Cambridge, Cancer Research UK Cambridge Institute, Robinson Way, Cambridge, CB2 0RE, United Kingdom
[3] Institute of Computational Biology, Helmholtz Zentrum München, Neuherberg, Germany.
[4] MRC Human Genetics Unit, Institute of Genetics & Molecular Medicine, University of Edinburgh, Western General Hospital, Crewe Road, Edinburgh EH4 2XU
[5] Centre for Inflammation Research, The Queen's Medical Research Institute, University of Edinburgh, Little France Crescent, Edinburgh EH16 4TJ, United Kingdom
[6] TUM School of Life Sciences Weihenstephan, Technical University of Munich, Freising, Germany.
[7] Biomedical Center (BMC), Physiological Chemistry, Faculty of Medicine, LMU Munich
[8] TUM School of Medicine, Technical University of Munich, Munich, Germany.

[†] These authors contributed equally to this work
[*] Corresponding authors

Table of Contents

Supplementary Methods *3*

Experimental methods 3

Computational methods 4

Supplementary Figures *7*

Supplementary Fig. 1 7

Supplementary Fig. 2 9

Supplementary Fig. 3 11

Supplementary Fig. 4 13

Supplementary Fig. 5 15

Supplementary Fig. 6 16

Supplementary Fig. 7 18

Supplementary Fig. 8 20

Supplementary Fig. 9 21

Supplementary Fig. 10 23

Supplementary Fig. 11 24

Supplementary Fig. 12 25

Supplementary Fig. 13 27

Supplementary Fig. 14 29

References Supplementary Information *31*

Supplementary Methods

Experimental methods

RNA-seq library preparation and sequencing

Sequencing libraries were prepared using the standard Illumina Nextera XT. DNA Sample Preparation kit (Illumina, #FC-131-1096) and the combination of 384 Combinatorial Dual Indexes (Illumina- Set A to D, #FC-131-2001 to FC-131-2004). Using the Mosquito liquid handling robot, the Nextera XT chemistry was miniaturized reducing 10-times the manufacturers volume as previously described [1, 2] (Supp. Dataset 10-14). For the library preparation 500nL of the undiluted cDNA was transferred in a new 384 well-plate containing 1500nL of Tagmentation Mix (TD and ATM reagents). Accordingly, all Nextera XT reagents (NT, NPM and i5/i7 indexes) were added stepwise to a final library volume of 5µL per well. We prepared a 384 well-plate with the combination of the i5/i7 index adapters, which was used in our miniaturization protocols. The final PCR amplification was 12 cycles. After library preparation, 500nL from each well was pooled together to a final volume of ~192µl to perform a final AMPure XP bead (Beckman Coulter, #A63882) clean-up. For our single nuclei libraries, two consecutive clean-ups with a ratio of sample to bead 0,9X led to library sizes between 200 and 1000bp, with no trace of adaptors. The final libraries were assessed using a HS DNA kit in the Agilent Bioanalyzer.

Sequencing

All the final 384-pooled libraries were sequenced using Illumina HiSeq4000 NGS sequencer in a paired-end 150 bases length. Each 384-pooled library was sequenced in one lane leading on average to ~1 million reads per nuclei.

Liquid handling robots and miniaturization

As described before in the snRNAseq2 section, the use of automated liquid handler as the Mosquito HV, allowed the fast and accurate processing of 384 well-plates. For each of the steps in the cDNA synthesis and the library preparation a 'source' plate (384-well Low Volume Serial Dilution -LVSD, STP Labtech) was used for reagents. Importantly, the over-aspirating option must be switched off to minimize the 'dead' reagent volume. Mosquito HV was placed in a flow cabinet maintained in pre-PCR and RNase and DNA free room. The detailed protocols for the Mosquito HV can be found in supplementary information.

Imagestream

Single nuclei isolation was conducted as described in previous sections. Briefly, nuclei were stained with Hoechst 33342 (Thermo Fisher Scientific, Cat No. 62249, 10µg/mL) and suspended in NSB. Bright field (Channel 01) and fluorescent (Channel 7) images were acquired on Amnis® ImageStream®XMark II Imaging Flow Cytometer (Luminex) at 40x magnification. Image and flow cytometry analyses were conducted using the IDEAS® Software (Amnis Corporation).

Immunofluorescence and analyses

Individual liver tissues were fixed in 4% paraformaldehyde for 24 hr, then transferred into 70% ethanol for a further 24 hr before embedding into paraffin blocks. Paraffin sections were cut in 3µm thickness, deparaffinised, rehydrated as described in previous sections. Antigen retrieval was performed by incubating slides either in citrate (pH6) or Tris-EDTA (pH9) solutions and heat-induced using a standard steamer for 30 min at a near-boiling temperature. Slides were washed and antibodies were prepared in 0.1% (v/v) Tween in TBS buffer (TBST). Sections were blocked with 3% donkey serum. For immunofluorescence, slides were stained by the following antibodies: anti-beta-catenin (Novus Biologicals, Cat No. AF1329-SP, 1:50); anti-Lyve1 (Abcam, Cat No. EPR21771, 1:1000). Sections were incubated with primary antibodies overnight at 4°C. Slides were thoroughly washed with TBST before incubated in secondary antibodies: Donkey-anti-goat AF647 IgG (H+L) (Thermofisher, Cat No. A-31573, 1:1000) or Donkey-anti-rabbit AF555 IgG (H+L) (ThermoFisher, Cat No. A-21432, 1:1000) for 1 hr at 4°C. For analysis of fibrosis model, mice liver sections were stained with anti-a-SMA-Cy3

conjugated antibody (Sigma, Cat No. C6198, 1:2000). All incubations were performed in dark and humidified chambers. Sections were stained with DAPI (ThermoFisher, Cat No. 62248, 1:1000) before mounted in VectaShield mounting media (Vectorlabs).

Confocal Microscopy and image analyses

Fluorescence images were obtained using the Zeiss 880 upright laser scanning confocal microscope with either 20x/1.0NA W Plan-Apochromat DIC or 63x/1.2NA W Korr C-Apochromat lens. Z stack of approximately 10µm thickness was imaged. Z stack with maximum projection was later generated by using the Fiji/Image J software. To measure areas (μm^2) of each visibly distinct nuclei with circularity of at least 0.7, DAPI images were processed using a 'Otsu' filter, followed by 'Smooth Edge' and 'Watershed' algorithms in Fiji software. Measurements of nuclei area were then converted to diameter in order to extrapolate ploidy levels, which was previously determined using RNA-FISH technique as described above. beta-catenin staining was then superimposed on DAPI to provide further information on cellular ploidy. Mononucleate 2n (light blue), binucleate 2n (dark blue), mononucleate 4n (light orange), and binucleate 4n (dark orange) were scored manually and colored using Illustrator software. Liver sinusoidal endothelial cells (LSECs) were identified by positive Lyve1 staining. LSEC diameter size was determined by Fiji/Image J software.

Computational methods

Data visualisation and clustering

The high dimensionality of the normalised expression matrix was reduced by calculating the first 50 principal components. Based on these, a neighborhood graph was constructed based on 15 nearest neighbors, where the connectivities between data points are calculated by Uniform Manifold Approximation and Projection for Dimension Reduction (UMAP) [3]. Data points were embedded using t-distributed stochastic neighborhood embedding (tSNE) based on the first 15 principal components and with a perplexity of 30. Additionally, data points were also visualized in UMAP and PCA embedding (Supp. Fig. 1F-G). *Louvain* clustering on the neighbourhood graph with a resolution of 0.2 was used to separate the cluster of non-parenchymal cells from the cluster of hepatocytes [4]. To further dissect the cell types in the non-parenchymal cluster, the hepatocytes were removed from the matrix. *Louvain* clustering with a resolution of 1 on the non-parenchymal cluster revealed 9 subclusters. Known marker genes were adapted from Aizarini *et at.* [5]. Epcam-positive epithelial cells were labelled as such if they had an expression level of >50 for *Epcam*.

To deeply investigate whether subgroups within the hepatocyte cluster can be found, *Louvain* clustering was done with resolution of 1 after re-calculating PCA and the neighborhood graph with 300 nearest neighbors. UMAP and tSNE were calculated as embeddings. No substructure was identified in neither the 2n or 4n hepatocyte population.

Cell cycle analysis and inter-cell type correlation

Cell cycle states were established using *cyclone* [6], as implemented in the scran R package [7]. Cells were classified into G1, S and G2M phase, based on their normalized gene counts (Fig. 2B and Supp. Fig. 2).

High ploidy analysis

Hoechst dye was used to stain all nuclei during nuclei isolation, allowing to distinguish between nuclei with 4n, 8n, and 16n ploidies using a FACS sorter (BD FACSAria 3) with a 100µm nozzle. Single nuclei were sorted in 384-well thin-walled PCR plates (BioRad, #HSP3901) followed by snRNA-seq2 using the new SMART-Seq Single Cell Kit (Takara, #634472) and the supplementary lysis buffer (Lysis Buffer 2, LB2). The original volumes were miniaturized 6 times using the Mosquito HV (SPTLabtech) according with the information provided in Supp. Dataset 10, 13 and 14. An additional step of bead clean-up was performed and the final cDNA product was quantified using a High Sensitivity DNA chip in a Bioanalyzer (Agilent). Subsequently, libraries were prepared using the Nextera XT kit and the pooled libraries from each plate were sequenced in one lane of an SP flowcell on an Illumina

NovaSeq6000 sequencer, at the same length as before (PE150). This chemistry provided higher sensitivity and numbers of reads and genes per nucleus compared to the 2n and 4n plates described above, therefore filtering was adapted in the following manner: nuclei were kept if they had at least 1% ERCC reads, between 1,000 and 12,000 genes, and coverage of 5,000 to 700,000 reads. Changes in distribution and mean expression between pairwise conditions were calculated as described above.

Gene ontology analysis

To find and visualise genes enriched in functionally relevant groups, the python package *gprofiler* (https://pypi.org/project/gprofiler-official/) was used, focusing on the ontology *biological process (BP).*

Transcriptional variability

According to the relationship between mean and coefficient of variation, lowly expressed genes have higher transcriptional variability. Hence, before calculating transcriptional variability between groups of interest, genes with mean normalized log-transformed expression smaller than 0.25 were removed, resulting in 2,102 remaining genes. The coefficient of variation (CV) was calculated on the normalised, log-transformed and batch-corrected matrix by making use of the formula described in Canchola *et al.* [8].

$$CV = \sqrt{e^{\sigma^2} - 1}$$

where $sigma^2$ is the variation of gene j in the group of interest.

A MannWhitneyU test was performed to see whether the CV differs significantly between 2n and 4n hepatocytes. Additionally, pairwise MannWhitneyU tests were performed between hepatocytes and any non-parenchymal cell type.

Furthermore, highly variable genes per group of interest were identified as genes that have a CV of greater than 1.7. This threshold was chosen after normalizing the ERCC-specific reads using the same formula as for the endogenous transcript (with the only adaptation of using 1,000 instead of 10,000 for scaling), log-transforming them, and calculating CV per ERCC, which resulted in 1.66 as the median plus one standard deviation CV for ERCC reads.

Pseudotemporal ordering based on markers of liver zonation

Non-parenchymal nuclei were removed from the matrix and zonation markers were obtained from [5, 9]. The hereby obtained normalized expression matrix was subset to only contain the zonation marker genes, resulting in a matrix of 1,061 nuclei times 1,742 zonation marker genes. Principle components, k-nearest neighbors and t-SNE as well as UMAP embedding were re-calculated for this matrix as described above. Furthermore, dimensionality reduction was done using diffusion map [10], thus ordering the nuclei along the zonation gradients. Based on marker gene expression and *Louvain* clustering (resolution=0.7, with cluster 1 on one side and combining clusters 0, 2 on the other side), the nuclei were separated into two groups, containing rather pericentral or rather periportal positioned nuclei, respectively. Per group, the percentage of diploid and tetraploid nuclei was calculated. Hereby, a 1.3-time relative enrichment of tetraploid nuclei was observed in the pericentral cluster. For the purpose of better visualisation in the bar plot, the 4n population was subsampled to have the same number of nuclei as the 2n population (Fig 5B).

To visualize changes in expression from pericentral to periportal, the top 30 most differential genes per group were taken and used to construct a PAGA path between these two groups[11]. Furthermore, to visualise the gene expression gradient for individual genes along pseudospace, the vector of diffusion pseudospace was binned into 10 bins between 0 and 1 and mean expression per bin was plotted.

Analysis of fibrosis model data

After single nuclei isolation and Hoechst staining, 2n and 4n nuclei from the CCl_4 treated mice and their matched wild type controls were sorted in 384 well-plates using a FACS sorter (BD FACSAria 3). The percentages of the nuclei ploidy states were recorded and analysed (Supp. Figure 13C and D). Both 2n

and 4n single nuclei from the liver of treated with CCl_4 and their WT control mice, were sorted per well in the same plates and processed for snRNA-seq2, using the new SMART-Seq Single Cell kit (Takara) exactly as above (High ploidy analysis section). This chemistry provided higher numbers of reads and genes per nucleus compared to the 2n and 4n plates described above using SMARTer v4 (Takara), therefore filtering was adapted in the following manner: nuclei were kept if they had between 30,000 and 1,000,000 reads. After normalisation and the removal of ERCCs, nuclei with >300,000 gene-length normalized counts were removed. Downstream processing was performed as described above. Non-parenchymal nuclei were again distinguished from hepatocytes through an initial *Louvain* clustering with a resolution of 0.2. Differential expression analysis, as well as pseudotemporal ordering based on zonation markers, was done separately for nuclei from wild-type and from CCl_4-treated animals.

Comparing improved protocol to the old lysis buffer and 10X experiments

The plate without additional lysis buffer (LB2) (SNI-668) was pre-processed as described above. The number of genes per nucleus was compared between the plate without LB2 and the plates with LB2 (Supp. Fig. 2C). Furthermore, the overall number of protein-coding genes was compared between the plate without LB2 and snRNA-seq2 (Supp. Fig. 2 D). For potential downstream analysis from the plate without additional LB2, nuclei were kept here if they had at least 500 genes and 5000 read counts detected.

For comparative purposes, additional 10X experiments on sorted 2n and 4n nuclei were performed and analysed. Reads were aligned to the mm10 reference genome using *10x Genomics Cell Ranger v4.0.0* [12]. Furthermore, another 10X experiment on sorted 4n nuclei with additional LB4 was performed. Nuclei with 0 genes detected were removed as well as genes with 0 expression. The number of genes detected per nucleus as well as the number of unique genes per method were compared to the results from the plate-based approaches with and without LB2 (Suppl Fig 2 E-F)

Comparison to publicly available single cell RNA-seq data sets

Data were obtained from either GEO (Accession numbers GSE84498, GSE124395, GSE148339 [5,13,14] or from https://doi.org/10.6084/m9.figshare.5829687.v7 and https://doi.org/10.6084/m9.figshare.5968960.v2 [15]). From unfiltered data, genes with zero expression across cells were removed as well as cells that did not express any genes. Numbers of genes expressed per cell and data set were visualized in violin plots (Fig. 1C). To compare the overall number of genes detected in each method, only protein coding genes were taken and their overlaps between methods were depicted in Venn diagrams (https://github.com/LankyCyril/pyvenn/blob/master/pyvenn-demo.ipynb; Supp. Fig. 2D).

In order to correlate gene expression between snRNA-seq2 data and *Tabula muris* smart-seq2 data [15], the raw count matrix based on exonic reads was taken. Furthermore, in the pairwise comparison, both data sets were subset to only contain hepatocytes. Correlation between log_2 of the mean gene expression in both datasets was calculated by Pearson correlation.

Supplementary Figures

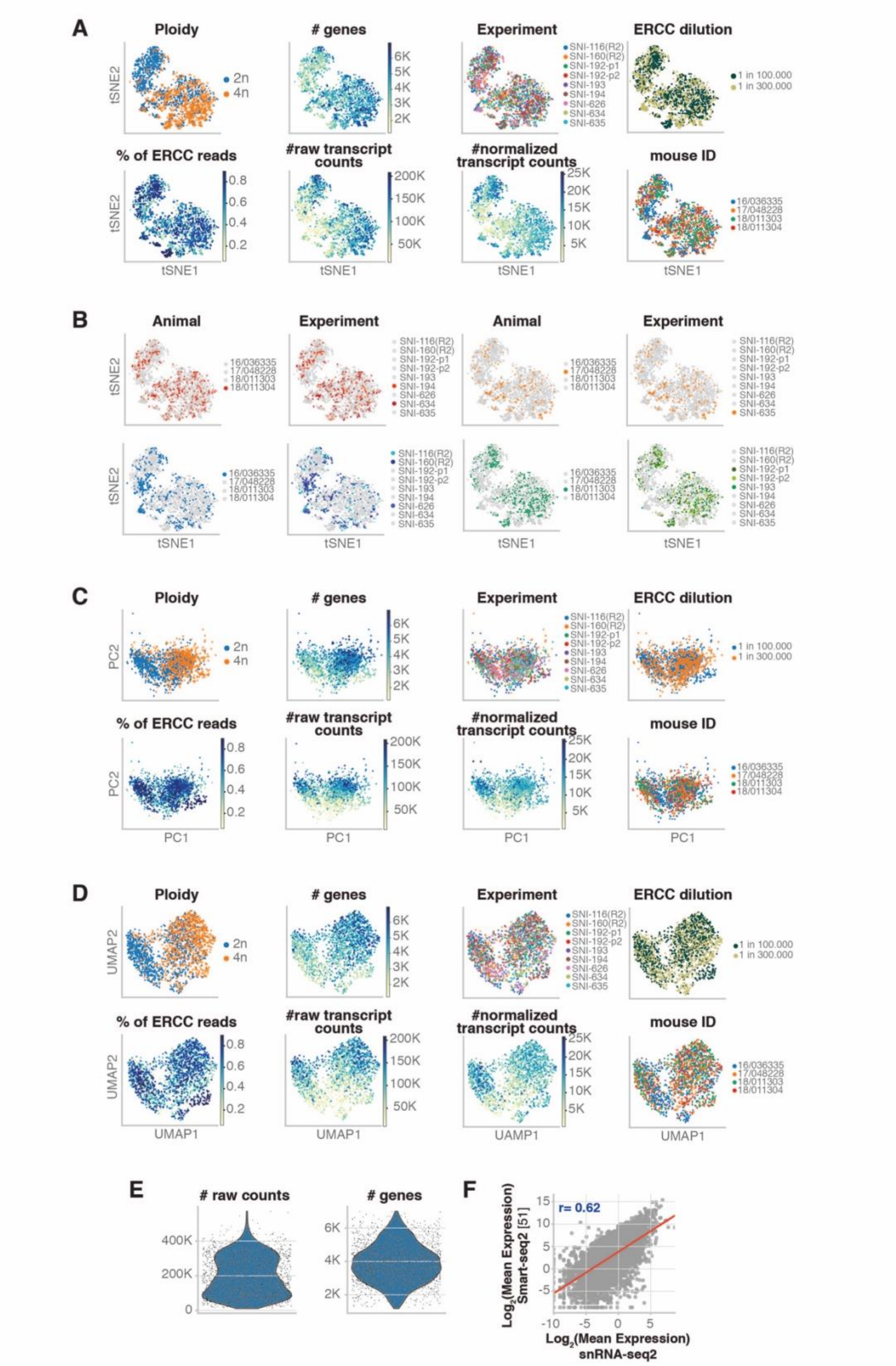

Supplementary Fig. 1

A) t-distributed stochastic neighborhood embedding (tSNE) colored by (first row, left to right) ploidy status, number of genes detected per nucleus, experimental batches, External RNA Control Consortium

(ERCC) spike-in dilutions, (second row, left to right) percentage of reads mapping to ERCCs per nucleus, number of raw transcripts reads per nucleus, number of normalized transcript counts per nucleus, and respective ID of mice used in the experiment. **B)** tSNE of mouse ID and corresponding experimental batch. Plots from A) were replicated and visualized as **C)** principal component analysis (PCA) plots and **D)** Uniform Manifold Approximation and Projection **(**UMAP) plots. **E)** Violin plot showing the distributions of raw counts (left) and number of genes (right) per nucleus. **F)** Scatter plot depicting the mean expression of genes for Smart-seq2 single cell data (y-axis) against snRNA-seq2 (x-axis), including regression line (rho=0.62).

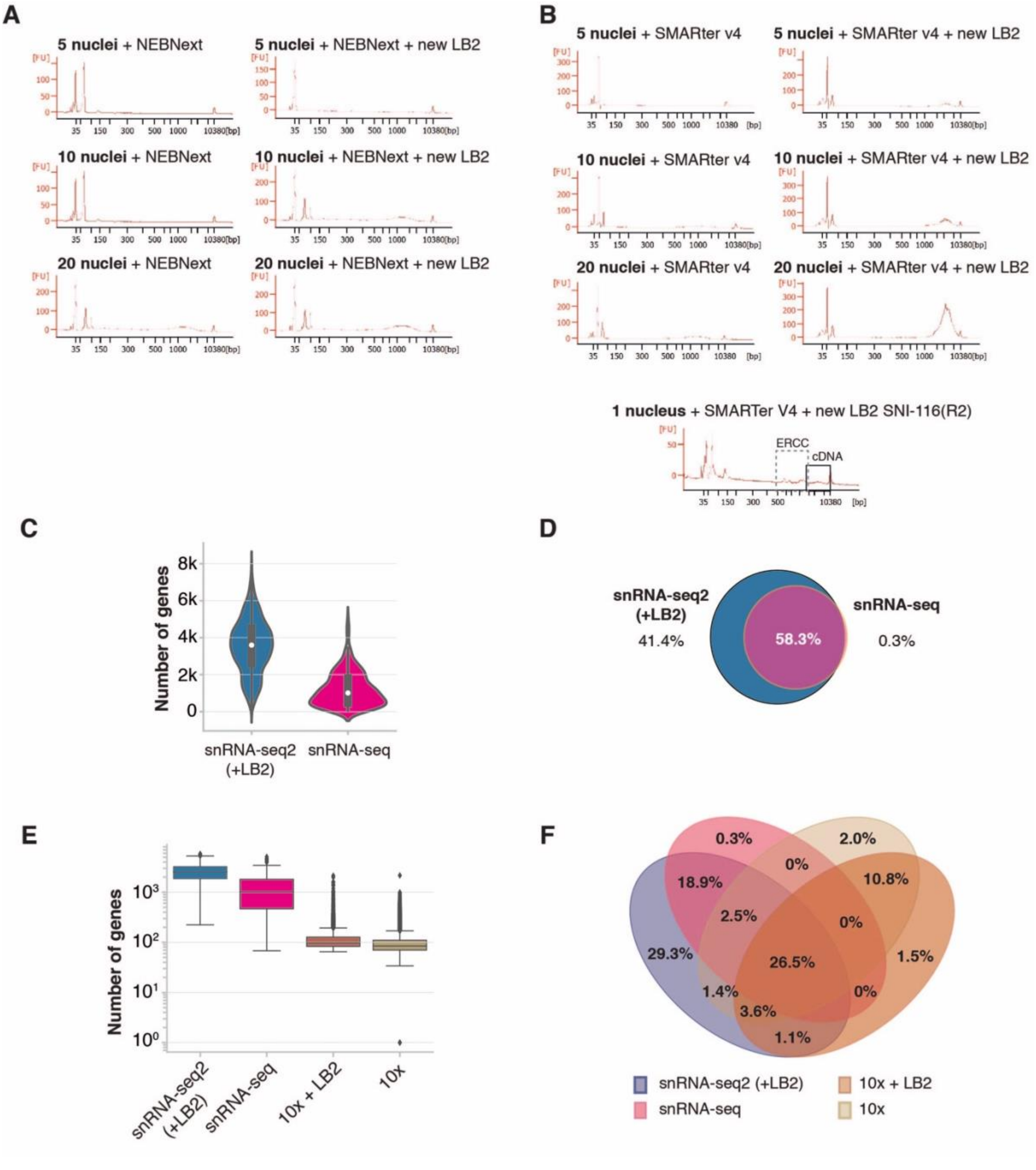

Supplementary Fig. 2

A) Bioanalyzer plots showing the increased cDNA concentration with the new snRNAseq2 lysis buffer with NEBnext and **B)** with SMARTer v4 (Takara). **C)** Comparison of the number of genes detected per nucleus using snRNA-seq2 with the new lysis buffer (+LB2) and the unmodified represented by violin plots comparing the number of genes per nucleus snRNA-seq2 (+LB2) and snRNA-seq. In violin plot, boxplots indicate median number of genes per method (white dot). The lower and upper ends of the boxes correspond to first and third quartiles, respectively, while the whiskers extend to minimum (first quartile minus 1.5 x the inter-quartile range (IQR)), and maximum (third quartile plus 1.5 x IQR). Any data points beyond the whiskers are only represented through their density distribution in the surrounding violin plots. **D)** Venn diagram comparing the overall number of genes detected using snRNA-seq2 (+LB2) and snRNA-seq. **E)** Box plots comparing the number of genes per nucleus between snRNA-seq2 (+LB2), snRNA-seq, 10x+LB2, and 10x. Box plots show the median number of genes per method. The lower and upper ends of the boxes correspond to first and third quartiles,

respectively, while the whiskers extend to minimum (first quartile minus 1.5 x the inter-quartile range (IQR)), and maximum (third quartile plus 1.5 x IQR). Data points beyond the whiskers are shown as individual points. **F)** Venn diagram comparing the overall number of genes detected using snRNA-seq2 (+LB2) and snRNA-seq, 10x+LB2, and 10x.

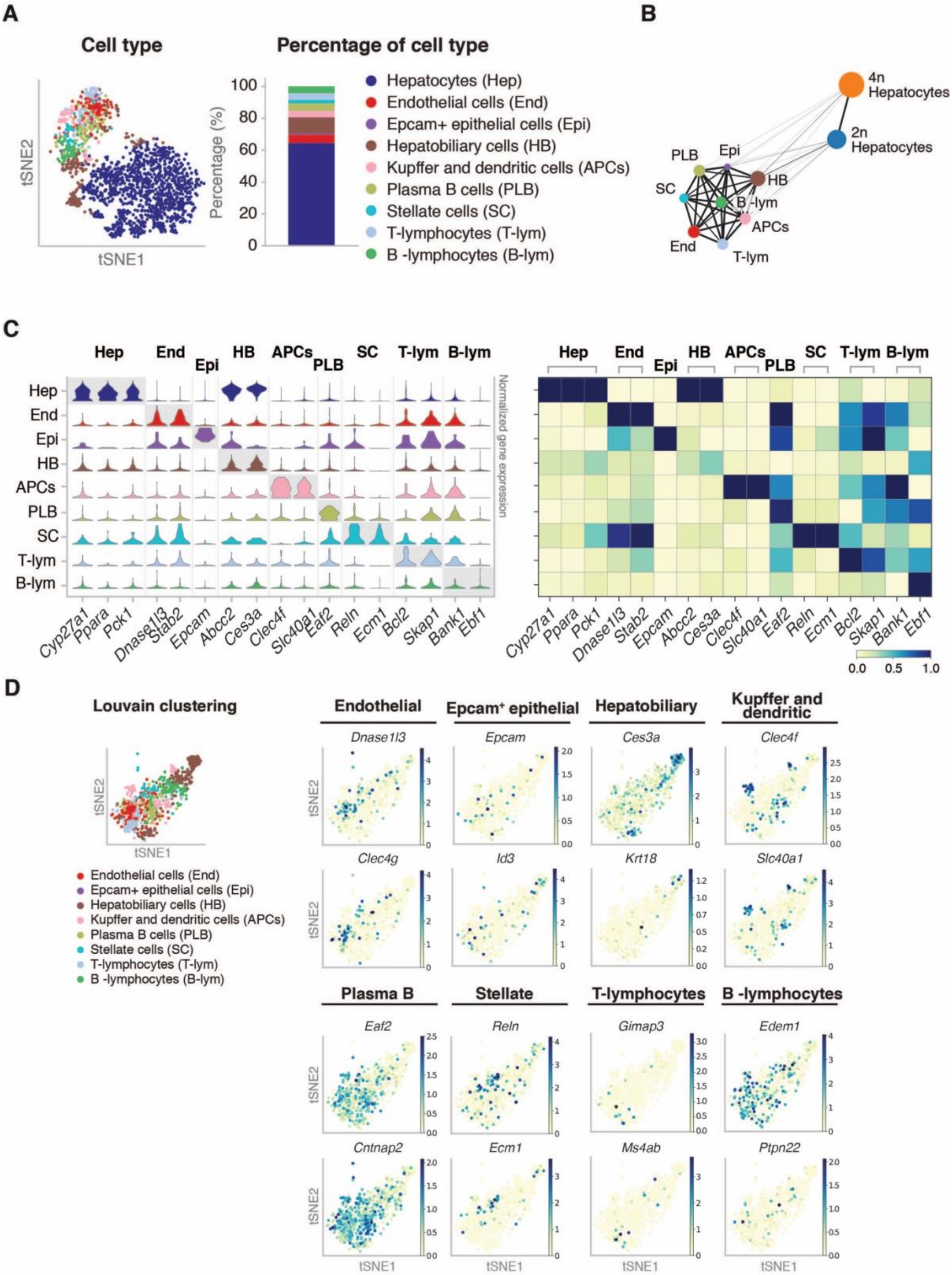

Supplementary Fig. 3

A) tSNE embedding colored by all cell types identified using snRNA-seq2 (left): bar plot depicting the percentages of the detected cell types (right). **B)** Partition-based graph abstraction **(**PAGA) showing the connection between all different cell types detected in snRNA-seq2. **C)** Violin plot (left) and matrix plot (right) showing the relative gene expression levels of marker genes in all cell types identified (y-axis: scaled, log-transformed, normalized counts). **D)** tSNE embedding of all non-parenchymal nuclei

colored by *Louvain* clusters with respective inferred cell type annotation (left). tSNE colored by representative marker gene expression for each major cell type population of the mouse liver.

A

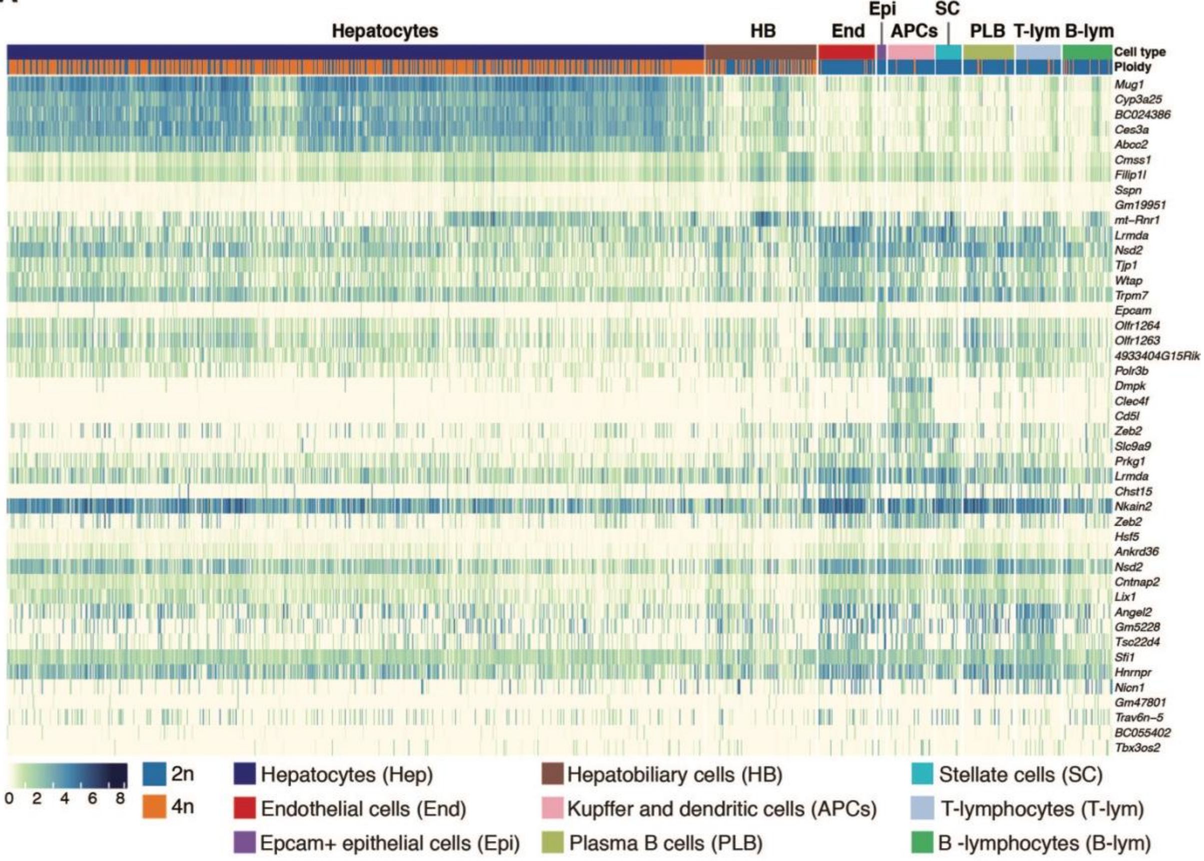

B

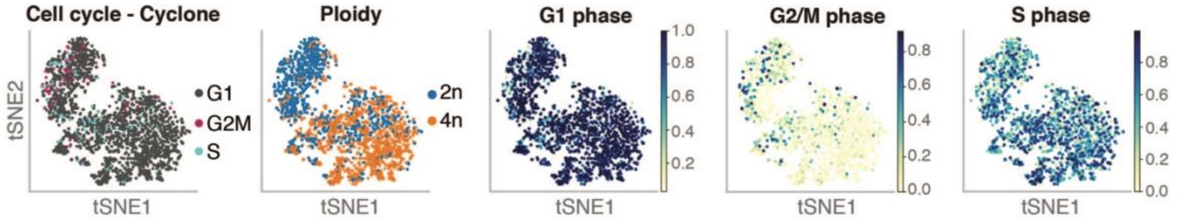

C

	Hep	End	Epi	HB	APC	PLB	SC	T-lym	B-lym	All
G1	1006	72	10	148	61	68	30	54	59	1508
G2/M	13	9	1	8	3	3	5	10	12	64
S	42	5	1	11	5	5	3	2	3	77
All	1061	89	12	167	69	76	38	66	74	1649

	2n Hep	2n other clusters	4n Hep	4n other clusters	All
G1	278	404	728	98	1506
G2/M	11	45	2	6	64
S	31	30	11	5	77
All	320	479	741	109	1649

D

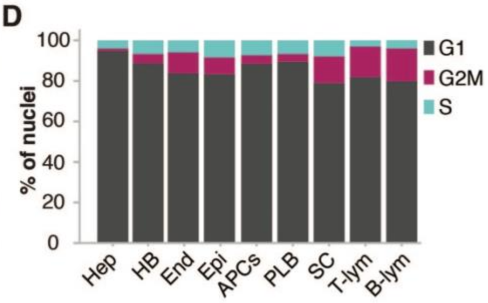

Supplementary Fig. 4

A) Heatmap showing the gene expression of top 5 differentially expressed genes between all different cell types (colored by log-transformed, normalized counts). **B)** t-SNE colored by cell cycle phases assigned by *Cyclone*, ploidy status, G1-scores, G2M-scores, and S-scores. **C)** Tables with the number of nuclei in the respective cell cycle phase per cell type (upper), and per ploidy level according to low

resolution *Louvain* clusters (lower). **D)** Bar graph showing the percentage of nuclei in respective cell cycle phase per cell type.

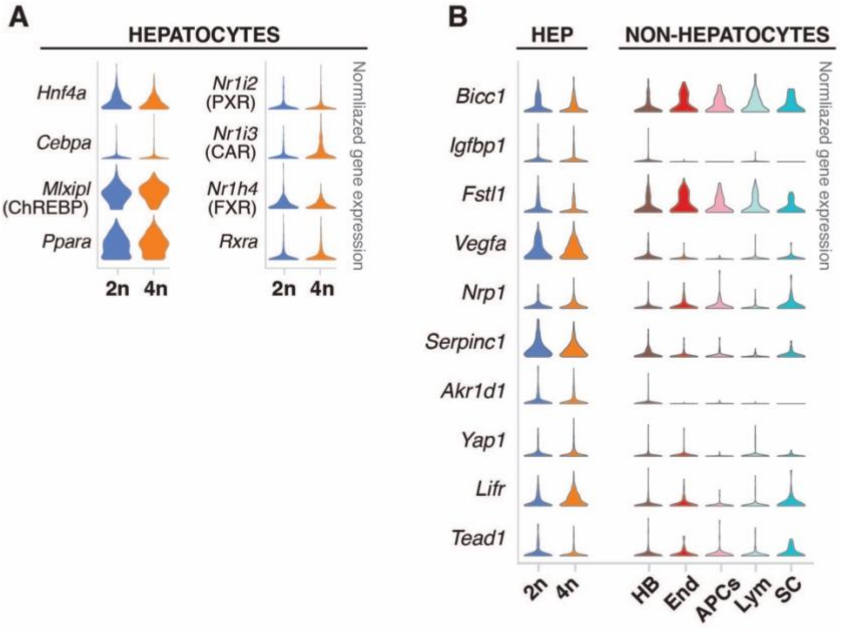

Supplementary Fig. 5

A) Violin plots showing the gene expression distribution in key liver-specific transcription factors in 2n and 4n hepatocytes. **B)** Violin plots showing the expression of disease-related marker genes across cell types in young wildtype mice livers (y-axis: scaled, log-transformed, normalized counts)**.**

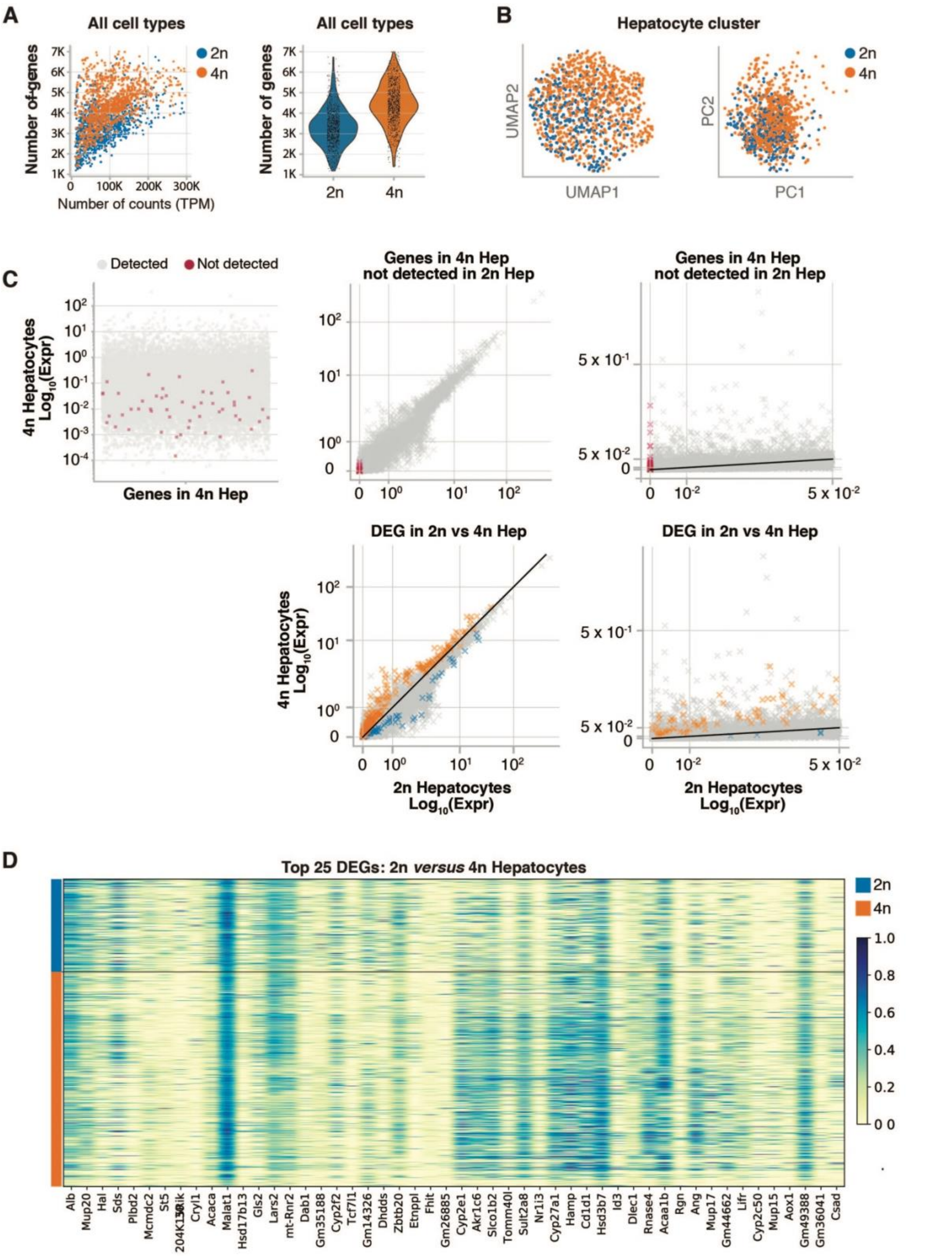

Supplementary Fig. 6

A) Scatterplot showing the number of genes per normalized counts for all cells colored by their ploidy level (left); violin plot showing a 1.35-fold increase in number of genes detected from 4n nuclei in comparison to 2n nuclei (right). **B)** UMAP (left) or PC (right) embedding of the hepatocyte cluster. **C)** Strip plot showing mean expression of all genes in 4n with genes not detected in 2n marked in red (left); Scatterplot showing mean expression of genes in 4n *vs* 2n with genes not expressed in 2n marked in red

(middle, top) and DEGs marked in orange (up in 4n) and blue (up in 2n) (middle, bottom); Zoom into lowly expressed genes showing mean expression of genes in 4n vs 2n with genes not expressed in 2n marked in red (right, top) and DEGs marked in orange (up in 4n) and blue (up in 2n) (right, bottom). **D)** Heatmap showing gene expression of the top 25 differentially expressed genes between 2n and 4n hepatocytes (colored by log-transformed, normalized counts).

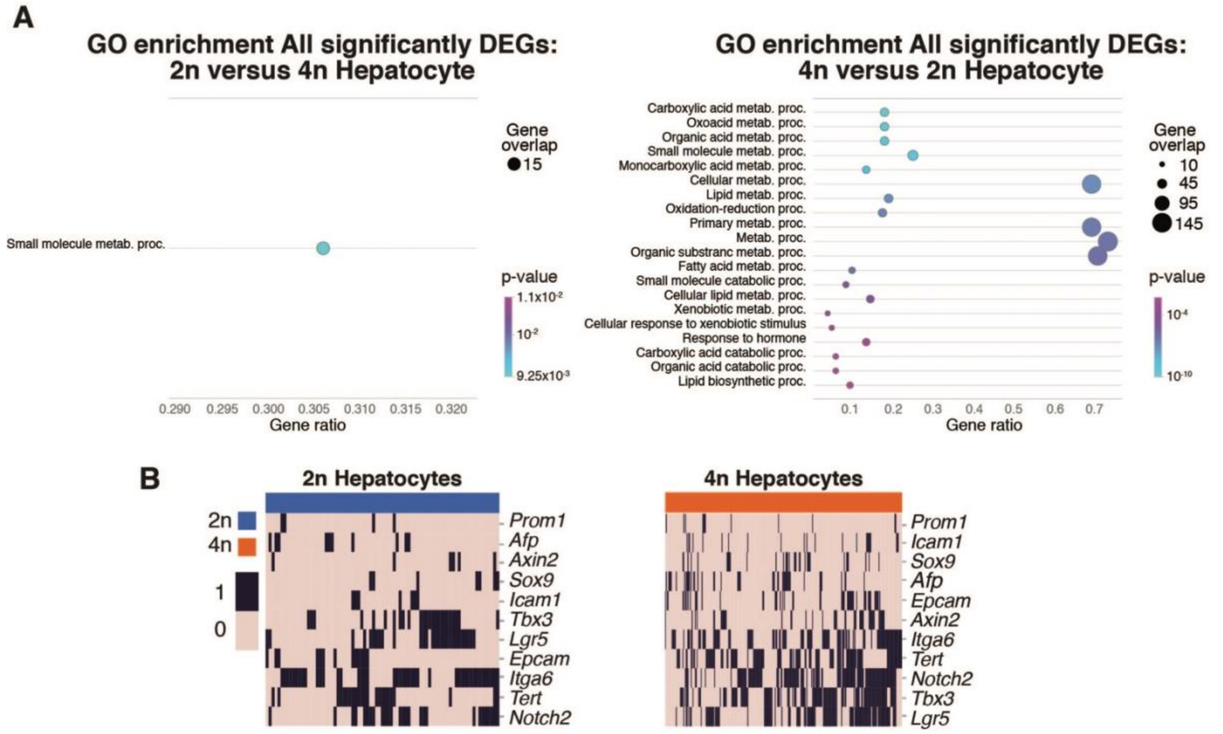

C

Gene	% All	% 2n	% 4n
Notch2	47	34	50
Tbx3	45	32	49
Itga6	44	54	41
Lgr5	42	35	44
Tert	40	29	44
Epcam	18	16	18
Afp	16	13	16
Axin2	15	8	16
Sox9	11	9	12
Icam1	8	9	8
Prom1	4	5	4

D

No. stem cell markers	% All	% 2n	% 4n
2	35.7	43.0	33.7
3	26.1	19.0	28.1
4	17.3	10.1	19.3
1	9.3	20.3	6.3
5	7.7	5.1	8.4
6	3.6	2.5	3.9
7	0.3	0	0.4

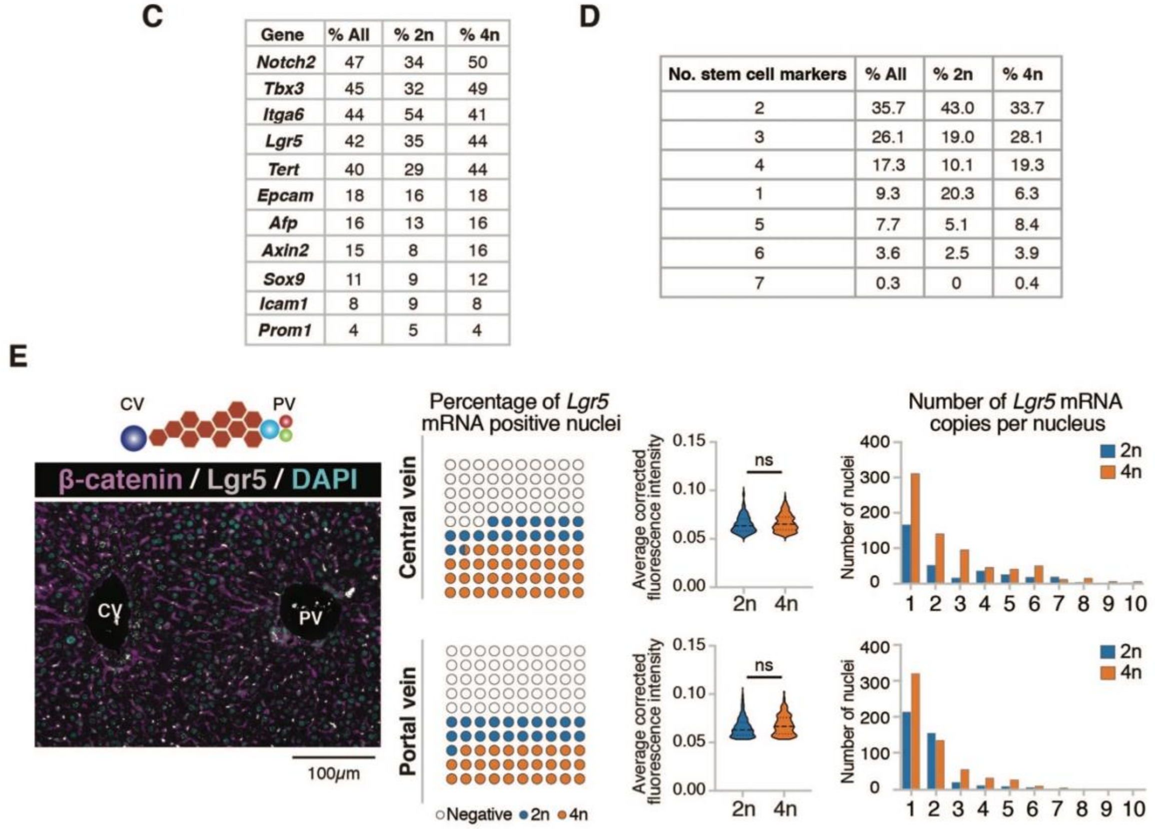

Supplementary Fig. 7

A) Gene set enrichment plots of all significantly differential expressed genes upregulated in 2n hepatocytes (left), and 4n hepatocytes (right). **B)** Heatmap showing binary expression of stem cell markers in 2n (left) and 4n hepatocytes (right); linkage is calculated based on Jaccard distances. **C)** Percentage of nuclei (all, 2n hepatocytes, and 4n hepatocytes), in which respective stem/progenitor markers are detected. **D)** Percentage of nuclei (all, 2n hepatocytes, and 4n hepatocytes), in which a number of stem cell markers (2-7) are co-expressed. **E)** Representative image of IF/RNA-FISH co-

detection analysis, showing expression levels of *Lgr5* (gray) mRNA, b-catenin (magenta) and DAPI (cyan) in a liver lobule (left) (n=3). Nuclei within 50µm distance of pericentral or periportal veins were categorized as CV or PV respectively. The percentage of *Lgr5* positive nuclei were quantified and scored according to their ploidy status, which was determined by the nuclei diameter size (µm). The average of fluorescence intensity corrected against background intensity was illustrated in violin plots, in which an unpaired t-test was performed (ns: not significant). The number of *Lgr5* mRNA copies per nucleus in 2n and 4n hepatocytes is expressed in a bar graph (right).

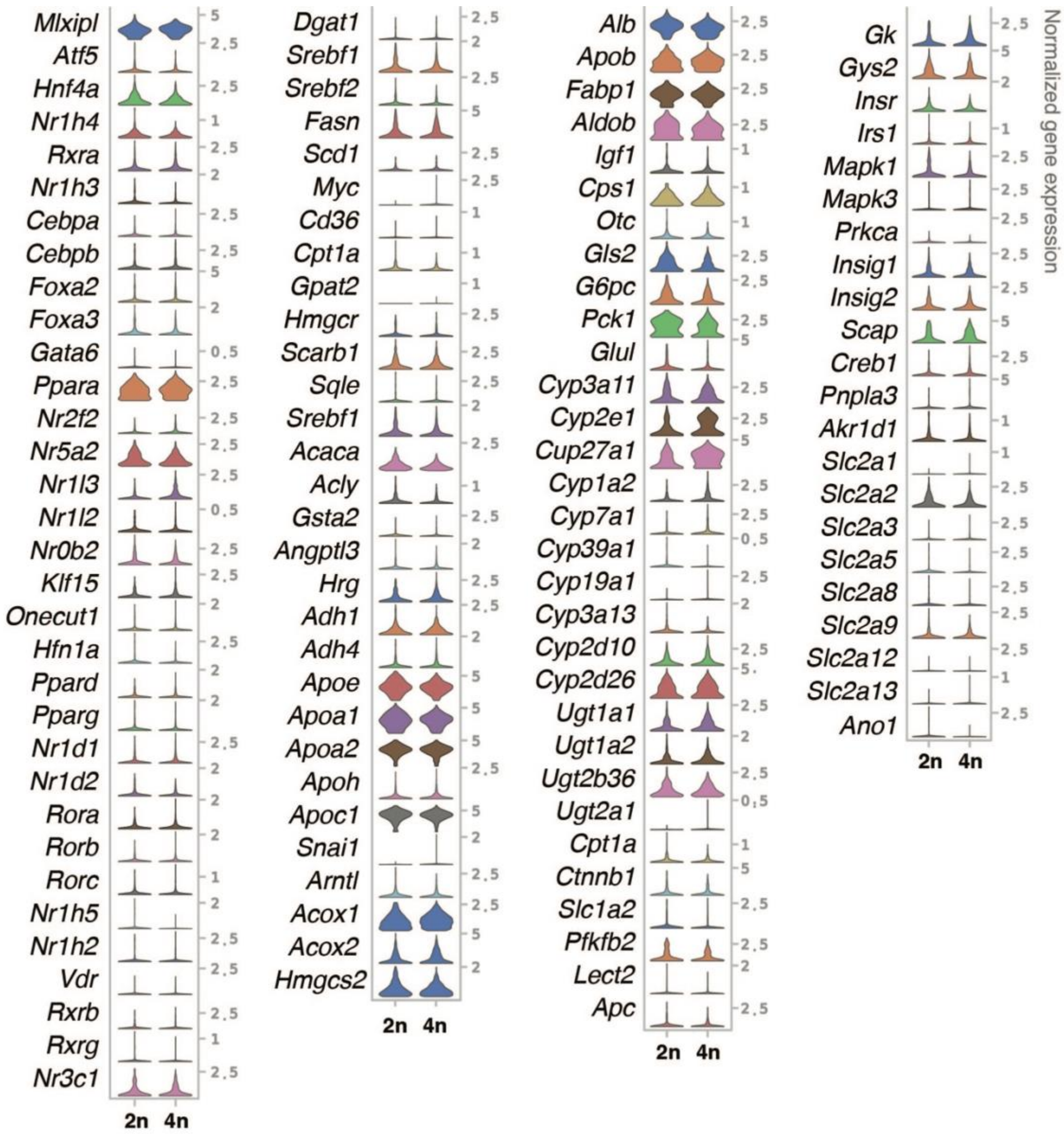

Supplementary Fig. 8
Violin plots showing the expression profiles of relevant genes in hepatic energy homeostasis and metabolism of xenobiotics (y-axis: scaled, log-transformed, normalized counts).

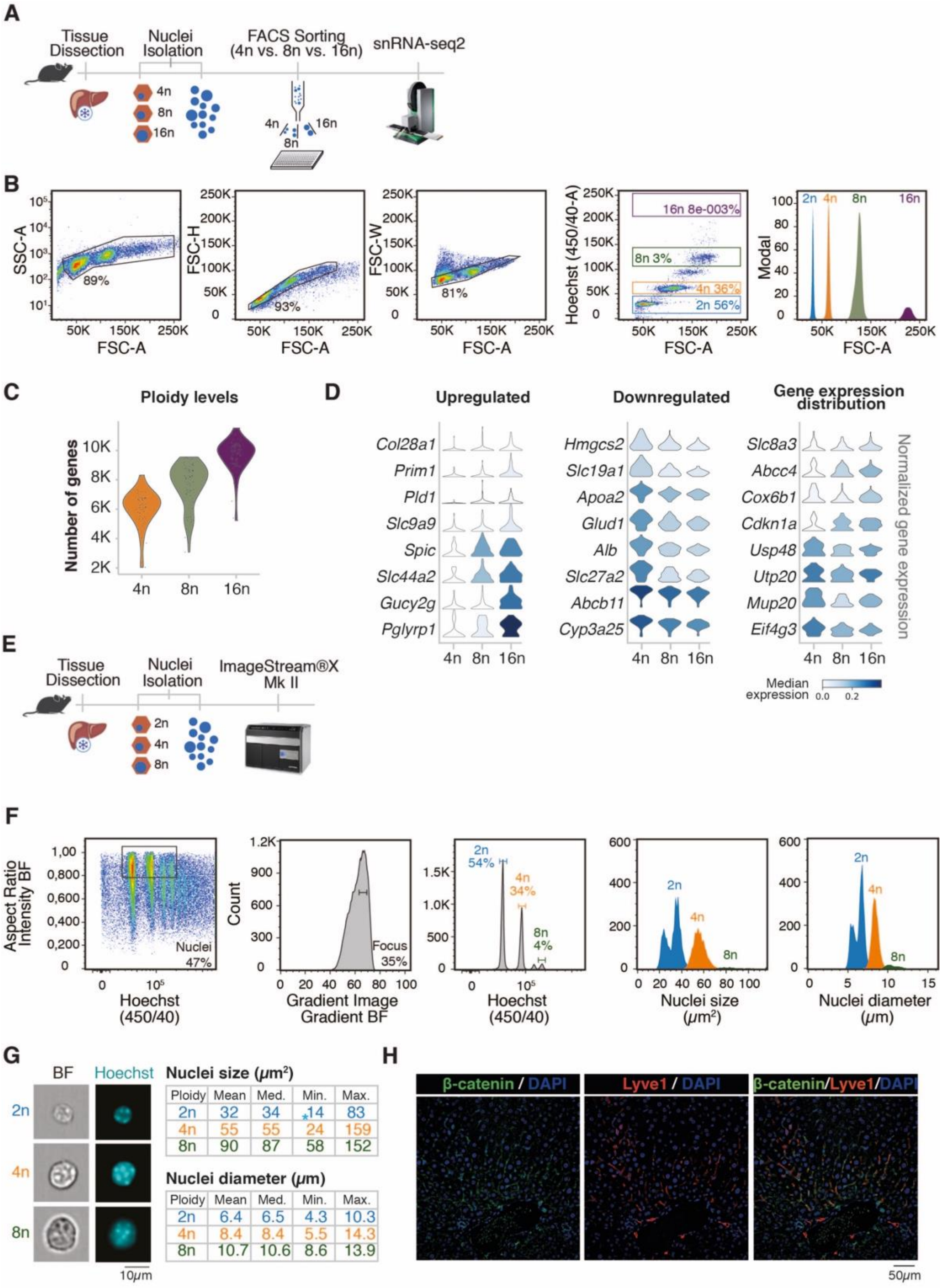

Supplementary Fig. 9

A) Schematic representation of the snRNA-seq2 pipeline for the analysis of 4n, 8n or 16n hepatocytes. **B)** Representative gating strategy on FACSAria III for sorting 2n, 4n, 8n or 16n nuclei. **C)** Violin plot showing the number of genes detected in hepatocytes of different ploidy. **D)** Violin plots showing genes that are significantly upregulated (left), downregulated (middle) or significantly change in their

expression distribution (right) with an increase in ploidy status (y-axis: scaled, log-transformed, normalized counts). **E)** Schematic representation of the pipeline used to visualize hepatocytes on ImageStream X MK II. **F)** Representative gating strategy and **G)** images of hepatocytes with ploidy status indicated on ImageStream®X MK II (n=2). **H)** Representative immunofluorescence images detecting beta-catenin (green), lyve-1 (red) and DAPI (blue) in PFA fixed mouse liver sections (n=1).

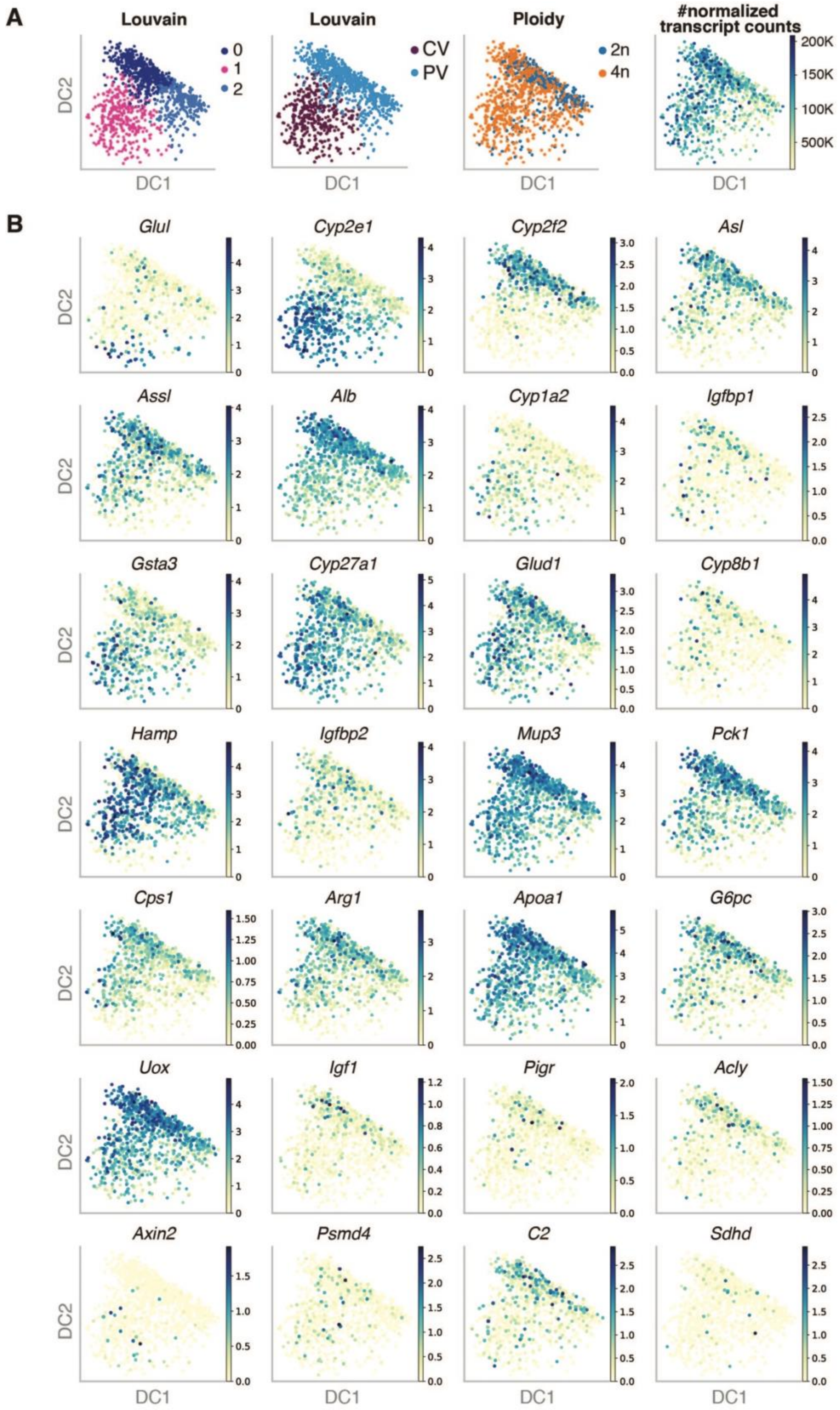

Supplementary Fig. 10
A) Diffusion map based on zonation markers, colored by *Louvain* clusters (left), aggregated by zone (middle left), ploidy (middle right), and normalized transcript counts (right). **B)** Diffusion maps colored by the expression of selected marker genes.

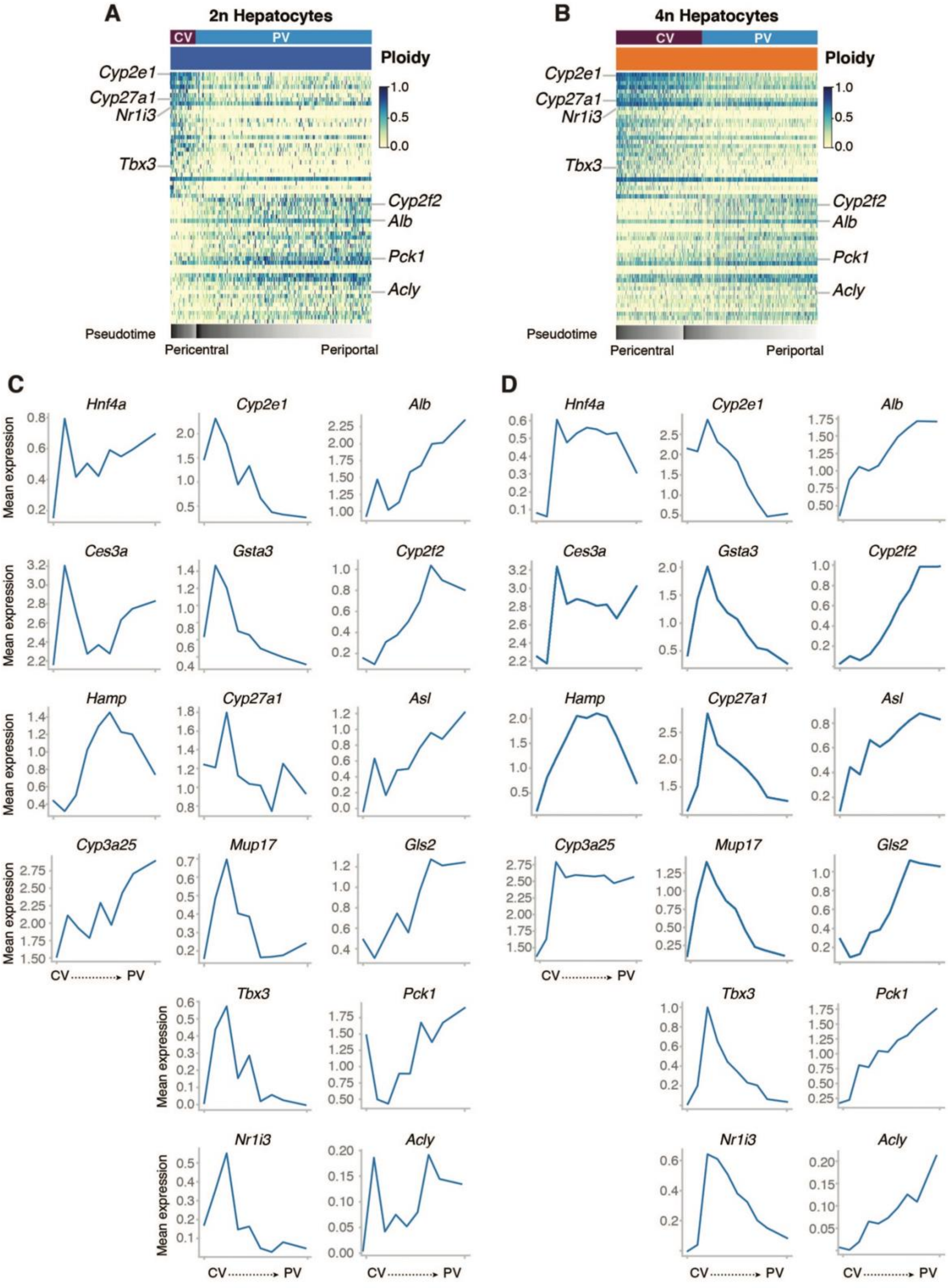

Supplementary Fig. 11

Heatmap showing gene expression of top differentially expressed genes in **A)** 2n and **B)** 4n nuclei. Line plots depicting the mean expression of representative zonation markers along the diffusion pseudospace vector, ordered from CV to PV within a liver lobule in **C)** 2n and **D)** 4n nuclei (heat maps colored by log-transformed, normalized counts)

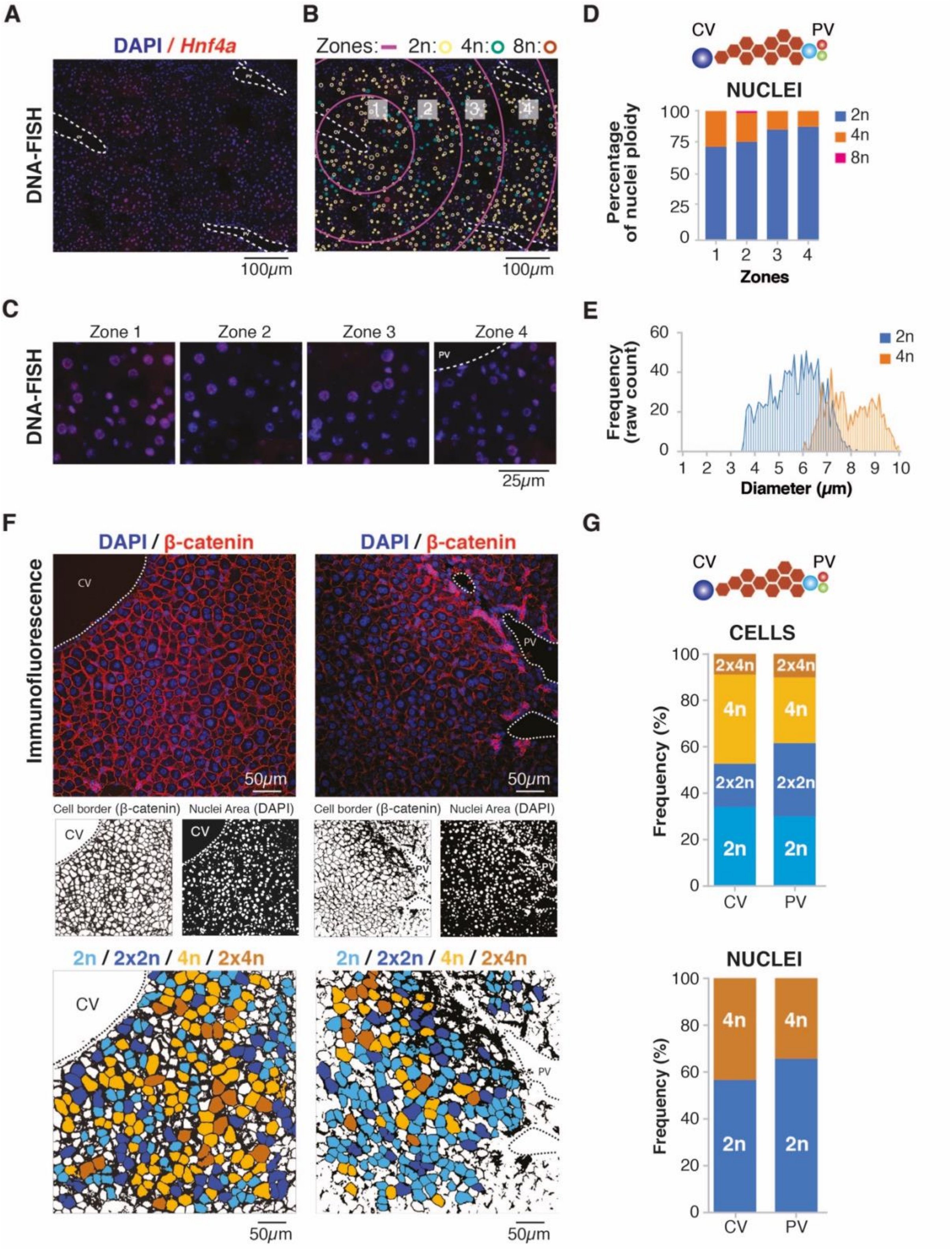

Supplementary Fig. 12

A) Representative image of DNA-FISH performed on paraformaldehyde (PFA) fixed mouse liver section (n=1). Tissue was probed for *Hnf4a* gene locus (red) and counterstained with DAPI (blue). **B-C)** liver section was divided into 4 zones from CV to PV, at 100µm intervals. Nuclei of each zone were scored as 2n, 4n or 8n as indicated. **D)** Quantification of nuclei ploidy expressed in percentage of total number of nuclei quantified in each zone. **E)** Graphical distribution of 2n or 4n nuclei in diameter (µm). **F)** Representative immunofluorescence images, detecting beta-catenin (red) and DAPI (blue) (n=2).

Nuclei were scored as either mononucleate 2n (light blue) or 4n (light orange), as well as binucleate 2n (dark blue) or 4n (dark orange) **G)** Quantification of ploidy in individual cells (upper) or as individual nuclei (lower) in CV or PV zones.

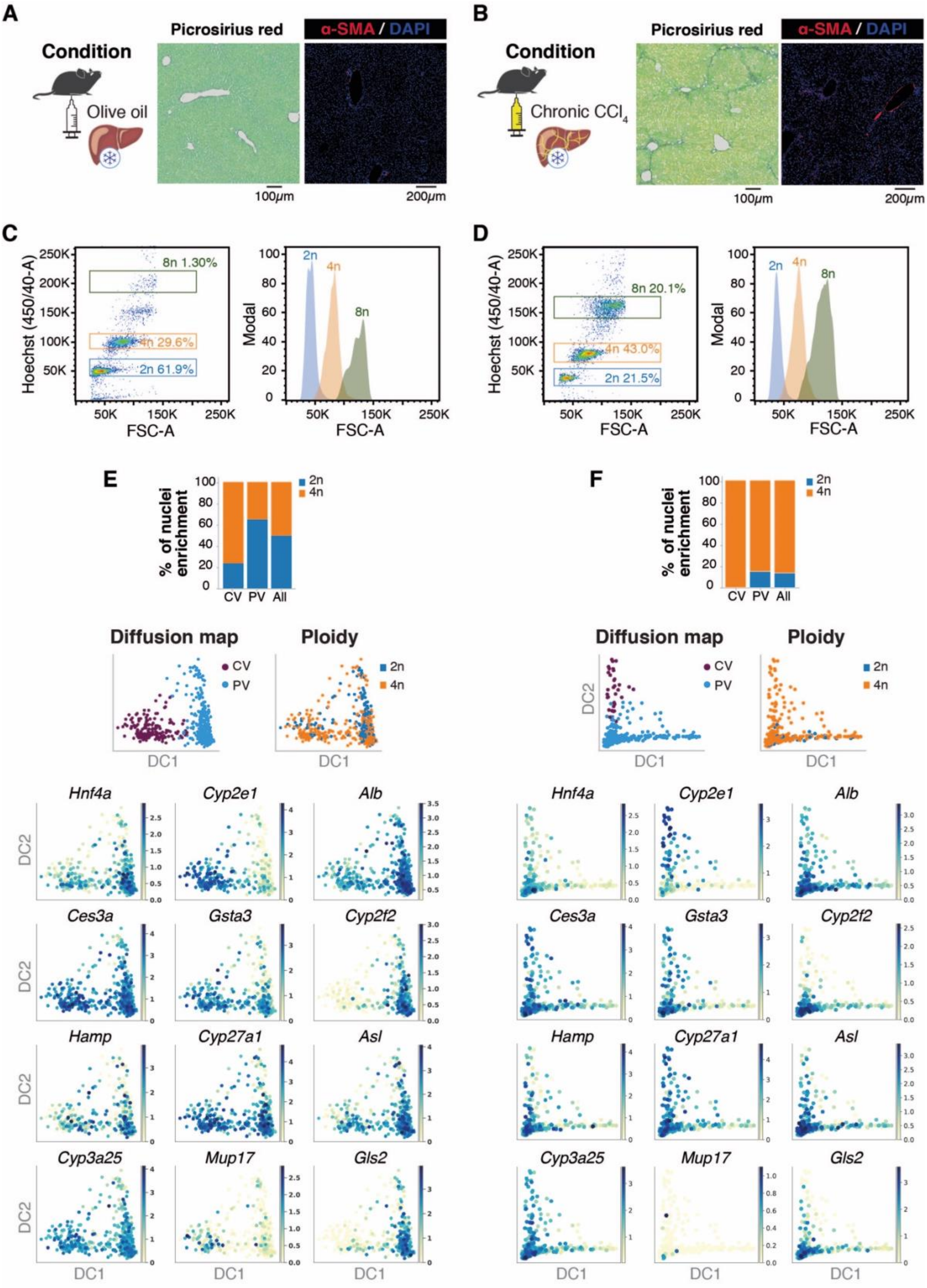

Supplementary Fig. 13

A-B) Schematic figure (left) of wild-type mouse receiving either olive oil (n=1) or CCl_4 (n=1) injection twice per week for 4 weeks, immunohistochemical staining of picrosirius red (middle) and immunofluorescence staining of alpha-SMA (red) and DAPI (blue) (right) of paraffin embedded mouse

livers, respectively. **C-D)** Representative gating strategy for sorting of 2n and 4n hepatocytes in control or chronic CCl_4 injured mice, respectively. Quantification of hepatocyte ploidy expressed in percentage per parental gate is shown for each gate. Bar plots showing the percentage of enrichment of nuclei ploidy in pericentral (CV) and periportal (PV) cluster in **E)** wild-type uninjured mice or **F)** chronic CCl_4 injured mice. Diffusion map based on zonation markers, colored by *Louvain* clusters and ploidy, as well as diffusion maps colored by the expression of zonation markers genes are also included.

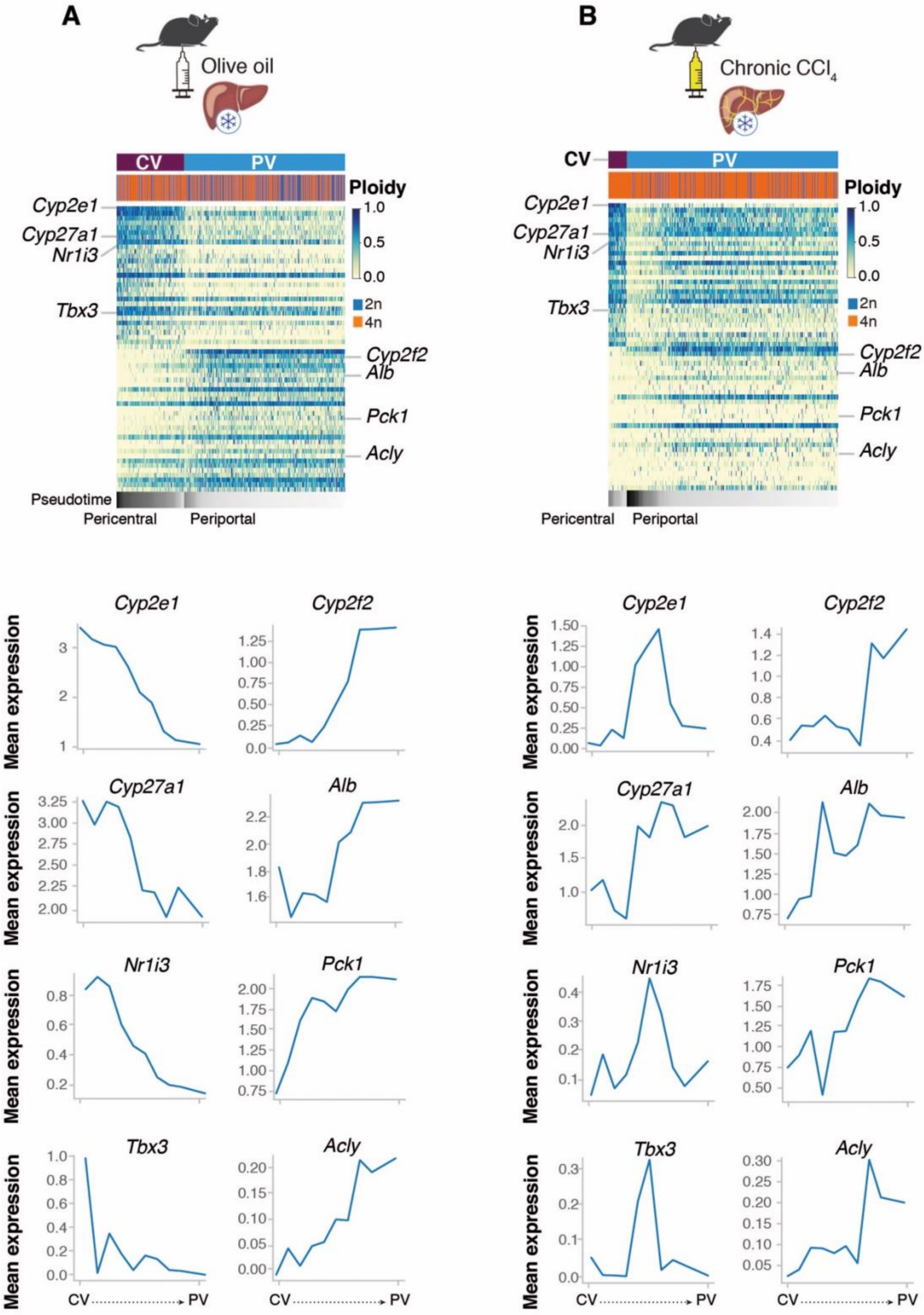

Supplementary Fig. 14

A) Schematic figure of wild-type mouse receiving olive oil (n=1) twice per week for 4 weeks (top). PAGA path heatmap showing the top differentially expressed genes within the pericentral (CV) and periportal (PV) cluster (middle) (colored by log-transformed, normalized counts). Line plots depicting the mean expression of representative zonation markers along the diffusion pseudospace vector, ordered from CV to PV (bottom). **B)** Schematic figure of wild-type mouse subjected to chronic liver injury via CCl_4 injection (n=1) twice per week for 4 weeks (top). PAGA path heatmap showing a disrupted

pseudospace ordering and an enrichment of 4n hepatocytes throughout the liver lobule (middle) (colored by log-transformed, normalized counts). Line plots depicting the mean expression of representative zonation markers along the diffusion pseudospace vector, showing that CCl_4-mediated liver injury severely disrupted how the diffusion pseudospace vector relates to zonation.

References Supplementary Information

1. Gasch AP, *et al.* Single-cell RNA sequencing reveals intrinsic and extrinsic regulatory heterogeneity in yeast responding to stress. *PLoS Biol* **15**, e2004050 (2017).

2. Mora-Castilla S, *et al.* Miniaturization Technologies for Efficient Single-Cell Library Preparation for Next-Generation Sequencing. *J Lab Autom* **21**, 557-567 (2016).

3. McInnes L., J. H, N. S, L. G. UMAP: Uniform Manifold Approximation and Projection. *Journal of Open Source Software* **3**, (2018).

4. Blondel VD, Guillaume J-L, Lambiotte R, Lefebvre E. Fast unfolding of communities in large networks. *Journal of Statistical Mechanics: Theory and Experiment* **2008**, P10008 (2008).

5. Aizarani N, *et al.* A human liver cell atlas reveals heterogeneity and epithelial progenitors. *Nature* **572**, 199-204 (2019).

6. Scialdone A, *et al.* Computational assignment of cell-cycle stage from single-cell transcriptome data. *Methods* **85**, 54-61 (2015).

7. Lun AT, McCarthy DJ, Marioni JC. A step-by-step workflow for low-level analysis of single-cell RNA-seq data with Bioconductor. *F1000Res* **5**, 2122 (2016).

8. Canchola J.A., Tang S., Hemyari P., Paxinos E., E. M. Correct use of percent coefficient of variation (%CV) formula for log-transformed data. *MOJ Proteomics Bioinform* **6**, 316-317 (2017).

9. Ben-Moshe S, *et al.* Spatial sorting enables comprehensive characterization of liver zonation. *Nat Metab* **1**, 899-911 (2019).

10. Haghverdi L, Buettner F, Theis FJ. Diffusion maps for high-dimensional single-cell analysis of differentiation data. *Bioinformatics* **31**, 2989-2998 (2015).

11. Wolf FA, *et al.* PAGA: graph abstraction reconciles clustering with trajectory inference through a topology preserving map of single cells. *Genome Biol* **20**, 59 (2019).

12. Zheng GX, *et al.* Massively parallel digital transcriptional profiling of single cells. *Nat Commun* **8**, 14049 (2017).

13. Halpern KB, *et al.* Single-cell spatial reconstruction reveals global division of labour in the mammalian liver. *Nature* **542**, 352-356 (2017).

14. Nault R, Fader KA, Bhattacharya S, Zacharewski TR. Single-Nuclei RNA Sequencing Assessment of the Hepatic Effects of 2,3,7,8-Tetrachlorodibenzo-p-dioxin. *Cell Mol Gastroenterol Hepatol* **11**, 147-159 (2021).

15. Tabula Muris C, *et al.* Single-cell transcriptomics of 20 mouse organs creates a Tabula Muris. *Nature* **562**, 367-372 (2018).
