# Supplementary material for: Single-nucleus RNA-seq2 reveals functional crosstalk between liver zonation and ploidy
Source: Nat Commun. 2021 Jul 12;12:4264. doi: 10.1038/s41467-021-24543-5 (PMC8275628; doi:10.1038/s41467-021-24543-5)
Supplement: Supplementary file 2 — Description of Additional Supplementary Files [file 41467_2021_24543_MOESM2_ESM.docx]

Description of Additional Supplementary Files

Title: Supplementary Dataset 1

Description: Biological and technical replicates

Title: Supplementary Dataset 2

Description: SMARterV4_Genes detected with and without LB2

Title: Supplementary Dataset 3

Description: Cell type markers

Title: Supplementary Dataset 4

Description: Gene expression list by cell type

Title: Supplementary Dataset 5

Description: Gene expression analysis 2n vs 4n

Title: Supplementary Dataset 6

Description: HVG and Coefficient of Variation in 2n and 4n

Title: Supplementary Dataset 7

Description: Changes in expression distribution in 2n and 4n and higher ploidy

Title: Supplementary Dataset 8

Description: Zonation_DE_genes

Title: Supplementary Dataset 9

Description: Zonation in CCl4 model

Title: Supplementary Dataset 10

Description: snRNAseq2_CalculationSheet
