# Supplementary material for: Single-nucleus RNA-seq2 reveals functional crosstalk between liver zonation and ploidy
Source: Nat Commun. 2021 Jul 12;12:4264. doi: 10.1038/s41467-021-24543-5 (PMC8275628; doi:10.1038/s41467-021-24543-5)
Supplement: Supplementary file 13 — Dataset 11 [file 41467_2021_24543_MOESM13_ESM.pdf]

```
C:\Users\Public\TTPLabTech\Mosquito\Protocols\CM Lab\snRNAseq2 Mosquito HV full plate. Lysis to cDNA.protocol

Tape pitch 4.5mm

5 position deck
Position:
1: [no plate]
2: [no plate]
3: TTP LVSD optimised ID
4: Bio-Rad PCR 384 HSP-3xxx in holder ID optimised
5: [no plate]

Aliquot 940nL from (P3, C1, R1, S1) to (P4, C1-12, R1, S1)
 Source (row, column) spacing: (1, 1)
 Destination (row, column) spacing: (1, 1)
 Contact first
 Multi-dispense
 Disable over aspiration

Pause  indefinitely at position 1 and display message "Freeze 384-well sorted pl
ate or Continue to add lysis buffer 2" - then home deck

Aliquot 2190nL from (P3, C3, R1, S1) to (P4, C1-12, R1, S1)
 Source (row, column) spacing: (1, 1)
 Destination (row, column) spacing: (1, 1)
 Contact first
 Change tips always
 Disable over aspiration

Pause  indefinitely at position 1 and display message "Rigorous vortexing, Spin
down and place plate in PCR for Lysis step - 6 min at 72oC, 10min on ice" - then
 home deck

Aliquot 1880nL from (P3, C5, R1, S1) to (P4, C1-12, R1, S1)
 Source (row, column) spacing: (1, 1)
 Destination (row, column) spacing: (1, 1)
 Contact first
 Change tips always
 Disable over aspiration

Pause  indefinitely at position 1 and display message "Vortex shortly, Spin down
 and place plate in PCR for RT step  -90 min at 42oC, 10min at 70oC" - then home
 deck

Aliquot 3750nL from (P3, C7, R1, S1) to (P4, C1-12, R1, S1)
 Source (row, column) spacing: (1, 1)
 Destination (row, column) spacing: (1, 1)
 Contact first
 Change tips always
 Disable over aspiration

Aliquot 3750nL from (P3, C8, R1, S1) to (P4, C13-24, R1, S1)
 Source (row, column) spacing: (1, 1)
 Destination (row, column) spacing: (1, 1)
 Contact first
 Change tips always
 Disable over aspiration

Aliquot 3750nL from (P3, C9, R1, S1) to (P4, C1-12, R1, S1)
 Source (row, column) spacing: (1, 1)
 Destination (row, column) spacing: (1, 1)
 Contact first
 Change tips always
 Disable over aspiration

Aliquot 3750nL from (P3, C10, R1, S1) to (P4, C13-24, R1, S1)
 Source (row, column) spacing: (1, 1)
 Destination (row, column) spacing: (1, 1)
 Contact first
 Change tips always
 Disable over aspiration

Pause  indefinitely at position 1 and display message " Vortex lightly, Spin dow
n and place plate in PCR for cDNA amplification step" - then home deck
```
