# Supplementary material for: Single-nucleus RNA-seq2 reveals functional crosstalk between liver zonation and ploidy
Source: Nat Commun. 2021 Jul 12;12:4264. doi: 10.1038/s41467-021-24543-5 (PMC8275628; doi:10.1038/s41467-021-24543-5)
Supplement: Supplementary file 14 — Dataset 12 [file 41467_2021_24543_MOESM14_ESM.pdf]

C:\Users\Public\TTPLabTech\Mosquito\Protocols\CM Lab\Nextera XT Mosquito HV full plate. cDNA to libraries (1i

Tape pitch 4.5mm

5 position deck
Position:
1: Bio-Rad PCR 384 HSP-3xxx in holder ID optimised; Plate Id: INDEXES
2: TTP LVSD; Plate Id: Source
3: Bio-Rad PCR 384 HSP-3xxx in holder ID optimised; Plate Id: Library Prep
4: Bio-Rad PCR 384 HSP-3xxx in holder ID optimised; Plate Id: dil cDNA
5: [no plate]

Aliquot 1500nL from (P2, C12, R1, S1) to (P3, C1-24, R1, S1)
Source (row, column) spacing: (1, 1)
Destination (row, column) spacing: (1, 1)
Contact first
Multi-dispense
Disable over aspiration

Copy 500nL from (P4, C1-24, R1, S1) to (P3, C1-24, R1, S1)
Source (row, column) spacing: (1, 1)
Destination (row, column) spacing: (1, 1)
Contact first
Change tips always
Disable over aspiration

Pause indefinitely at position 1 and display message "Spin down and place plate
in PCR for Tagmentation step, 10 min at 55 C plus 10 sec at 4C" - then home de
ck

Aliquot 500nL from (P2, C14, R1, S1) to (P3, C1-24, R1, S1)
Source (row, column) spacing: (1, 1)
Destination (row, column) spacing: (1, 1)
Contact first
Change tips always
Disable over aspiration

Pause indefinitely at position 1 and display message "Vortex vigorously, Spin d
own and keep in Room Temperature for 5 min" - then home deck

Aliquot 1500nL from (P2, C16, R1, S1) to (P3, C1-12, R1, S1)
Source (row, column) spacing: (1, 1)
Destination (row, column) spacing: (1, 1)
Contact first
Change tips always
Disable over aspiration

Aliquot 1500nL from (P2, C17, R1, S1) to (P3, C13-24, R1, S1)
Source (row, column) spacing: (1, 1)
Destination (row, column) spacing: (1, 1)
Contact first
Change tips always
Disable over aspiration

Copy 1000nL from (P1, C1-24, R1, S1) to (P3, C1-24, R1, S1)
Source (row, column) spacing: (1, 1)
Destination (row, column) spacing: (1, 1)
Contact first
Change tips always
Disable over aspiration

Pause indefinitely at position 1 and display message "Vortex briefly, Spin down
and place plate in PCR for Library Amplification step" - then home deck
