# Supplementary material for: Single-nucleus RNA-seq2 reveals functional crosstalk between liver zonation and ploidy
Source: Nat Commun. 2021 Jul 12;12:4264. doi: 10.1038/s41467-021-24543-5 (PMC8275628; doi:10.1038/s41467-021-24543-5)
Supplement: Supplementary file 15 — Dataset 13 [file 41467_2021_24543_MOESM15_ESM.pdf]

```
5 position deck
Position:
1: TTP LVSD; Plate Id: reagents
2: PCI clamp- Eppendorf twin.tecÆ 384; Plate Id: reaction
3: [no plate]
4: [no plate]
5: [no plate]

Home tape
Change pipettes at (P1, C1, R1, S1)
Aspirate 4800 nL from (P1, C1, R1, S1), well volume 0 µL, manual
height: 0.5 mm
Dispense 1600 nL to (P2, C1, R1, S1), well volume 0 µL
Dispense 1600 nL to (P2, C2, R1, S1), well volume 0 µL
Dispense 1600 nL to (P2, C3, R1, S1), well volume 0 µL
Aspirate 4800 nL from (P1, C1, R1, S1), well volume 0 µL, manual
height: 0.5 mm
Dispense 1600 nL to (P2, C4, R1, S1), well volume 0 µL
Dispense 1600 nL to (P2, C5, R1, S1), well volume 0 µL
Dispense 1600 nL to (P2, C6, R1, S1), well volume 0 µL
Aspirate 4800 nL from (P1, C1, R1, S1), well volume 0 µL, manual
height: 0.5 mm
Dispense 1600 nL to (P2, C7, R1, S1), well volume 0 µL
Dispense 1600 nL to (P2, C8, R1, S1), well volume 0 µL
Dispense 1600 nL to (P2, C9, R1, S1), well volume 0 µL
Aspirate 4800 nL from (P1, C1, R1, S1), well volume 0 µL, manual
height: 0.5 mm
Dispense 1600 nL to (P2, C10, R1, S1), well volume 0 µL
Dispense 1600 nL to (P2, C11, R1, S1), well volume 0 µL
Dispense 1600 nL to (P2, C12, R1, S1), well volume 0 µL
Aspirate 4800 nL from (P1, C1, R1, S1), well volume 0 µL, manual
height: 0.5 mm
Dispense 1600 nL to (P2, C13, R1, S1), well volume 0 µL
Dispense 1600 nL to (P2, C14, R1, S1), well volume 0 µL
Dispense 1600 nL to (P2, C15, R1, S1), well volume 0 µL
Aspirate 4800 nL from (P1, C1, R1, S1), well volume 0 µL, manual
height: 0.5 mm
Dispense 1600 nL to (P2, C16, R1, S1), well volume 0 µL
Dispense 1600 nL to (P2, C17, R1, S1), well volume 0 µL
Dispense 1600 nL to (P2, C18, R1, S1), well volume 0 µL
Aspirate 4800 nL from (P1, C1, R1, S1), well volume 0 µL, manual
height: 0.5 mm
Dispense 1600 nL to (P2, C19, R1, S1), well volume 0 µL
Dispense 1600 nL to (P2, C20, R1, S1), well volume 0 µL
Dispense 1600 nL to (P2, C21, R1, S1), well volume 0 µL
Aspirate 4800 nL from (P1, C1, R1, S1), well volume 0 µL, manual
height: 0.5 mm
Dispense 1600 nL to (P2, C22, R1, S1), well volume 0 µL
Dispense 1600 nL to (P2, C23, R1, S1), well volume 0 µL
Dispense 1600 nL to (P2, C24, R1, S1), well volume 0 µL
Change pipettes
Pause  indefinitely at position 3 and display message "seal the
plate, cfg, sort single cells, cfg, incubate at 72deg for 3 min,
ice, cfg" - then home deck
```

Aspirate 940 nL from (P1, C3, R1, S1), well volume 0 µL, manual height: 0.5 mm
Dispense 940 nL to (P2, C1, R1, S1), well volume 0 µL
Change pipettes
Aspirate 940 nL from (P1, C3, R1, S1), well volume 0 µL, manual height: 0.5 mm
Dispense 940 nL to (P2, C2, R1, S1), well volume 0 µL
Change pipettes
Aspirate 940 nL from (P1, C3, R1, S1), well volume 0 µL, manual height: 0.5 mm
Dispense 940 nL to (P2, C3, R1, S1), well volume 0 µL
Change pipettes
Aspirate 940 nL from (P1, C3, R1, S1), well volume 0 µL, manual height: 0.5 mm
Dispense 940 nL to (P2, C4, R1, S1), well volume 0 µL
Change pipettes
Aspirate 940 nL from (P1, C3, R1, S1), well volume 0 µL, manual height: 0.5 mm
Dispense 940 nL to (P2, C5, R1, S1), well volume 0 µL
Change pipettes
Aspirate 940 nL from (P1, C3, R1, S1), well volume 0 µL, manual height: 0.5 mm
Dispense 940 nL to (P2, C6, R1, S1), well volume 0 µL
Change pipettes
Aspirate 940 nL from (P1, C3, R1, S1), well volume 0 µL, manual height: 0.5 mm
Dispense 940 nL to (P2, C7, R1, S1), well volume 0 µL
Change pipettes
Aspirate 940 nL from (P1, C3, R1, S1), well volume 0 µL, manual height: 0.5 mm
Dispense 940 nL to (P2, C8, R1, S1), well volume 0 µL
Change pipettes
Aspirate 940 nL from (P1, C3, R1, S1), well volume 0 µL, manual height: 0.5 mm
Dispense 940 nL to (P2, C9, R1, S1), well volume 0 µL
Change pipettes
Aspirate 940 nL from (P1, C3, R1, S1), well volume 0 µL, manual height: 0.5 mm
Dispense 940 nL to (P2, C10, R1, S1), well volume 0 µL
Change pipettes
Aspirate 940 nL from (P1, C3, R1, S1), well volume 0 µL, manual height: 0.5 mm
Dispense 940 nL to (P2, C11, R1, S1), well volume 0 µL
Change pipettes
Aspirate 940 nL from (P1, C3, R1, S1), well volume 0 µL, manual height: 0.5 mm
Dispense 940 nL to (P2, C12, R1, S1), well volume 0 µL
Change pipettes
Aspirate 940 nL from (P1, C3, R1, S1), well volume 0 µL, manual height: 0.5 mm
Dispense 940 nL to (P2, C13, R1, S1), well volume 0 µL
Change pipettes
Aspirate 940 nL from (P1, C3, R1, S1), well volume 0 µL, manual height: 0.5 mm

Dispense 940 nL to (P2, C14, R1, S1), well volume 0 µL
Change pipettes
Aspirate 940 nL from (P1, C3, R1, S1), well volume 0 µL, manual height: 0.5 mm
Dispense 940 nL to (P2, C15, R1, S1), well volume 0 µL
Change pipettes
Aspirate 940 nL from (P1, C3, R1, S1), well volume 0 µL, manual height: 0.5 mm
Dispense 940 nL to (P2, C16, R1, S1), well volume 0 µL
Change pipettes
Aspirate 940 nL from (P1, C3, R1, S1), well volume 0 µL, manual height: 0.5 mm
Dispense 940 nL to (P2, C17, R1, S1), well volume 0 µL
Change pipettes
Aspirate 940 nL from (P1, C3, R1, S1), well volume 0 µL, manual height: 0.5 mm
Dispense 940 nL to (P2, C18, R1, S1), well volume 0 µL
Change pipettes
Aspirate 940 nL from (P1, C3, R1, S1), well volume 0 µL, manual height: 0.5 mm
Dispense 940 nL to (P2, C19, R1, S1), well volume 0 µL
Change pipettes
Aspirate 940 nL from (P1, C3, R1, S1), well volume 0 µL, manual height: 0.5 mm
Dispense 940 nL to (P2, C20, R1, S1), well volume 0 µL
Change pipettes
Aspirate 940 nL from (P1, C3, R1, S1), well volume 0 µL, manual height: 0.5 mm
Dispense 940 nL to (P2, C21, R1, S1), well volume 0 µL
Change pipettes
Aspirate 940 nL from (P1, C3, R1, S1), well volume 0 µL, manual height: 0.5 mm
Dispense 940 nL to (P2, C22, R1, S1), well volume 0 µL
Change pipettes
Aspirate 940 nL from (P1, C3, R1, S1), well volume 0 µL, manual height: 0.5 mm
Dispense 940 nL to (P2, C23, R1, S1), well volume 0 µL
Change pipettes
Aspirate 940 nL from (P1, C3, R1, S1), well volume 0 µL, manual height: 0.5 mm
Dispense 940 nL to (P2, C24, R1, S1), well volume 0 µL
Change pipettes
Pause indefinitely at position 3 and display message "seal the plate, cfg for 1min, incubate at 42deg for 3 hrs, 70deg for 10min, cfg, ice" - then home deck
Aspirate 4500 nL from (P1, C5, R1, S1), well volume 0 µL, manual height: 0.5 mm
Dispense 4500 nL to (P2, C1, R1, S1), well volume 0 µL
Change pipettes
Aspirate 4500 nL from (P1, C5, R1, S1), well volume 0 µL, manual height: 0.5 mm
Dispense 4500 nL to (P2, C2, R1, S1), well volume 0 µL
Change pipettes
Aspirate 4500 nL from (P1, C5, R1, S1), well volume 0 µL, manual

height: 0.5 mm
Dispense 4500 nL to (P2, C3, R1, S1), well volume 0 µL
Change pipettes
Aspirate 4500 nL from (P1, C5, R1, S1), well volume 0 µL, manual height: 0.5 mm
Dispense 4500 nL to (P2, C4, R1, S1), well volume 0 µL
Change pipettes
Aspirate 4500 nL from (P1, C5, R1, S1), well volume 0 µL, manual height: 0.5 mm
Dispense 4500 nL to (P2, C5, R1, S1), well volume 0 µL
Change pipettes
Aspirate 4500 nL from (P1, C5, R1, S1), well volume 0 µL, manual height: 0.5 mm
Dispense 4500 nL to (P2, C6, R1, S1), well volume 0 µL
Change pipettes
Aspirate 4500 nL from (P1, C5, R1, S1), well volume 0 µL, manual height: 0.5 mm
Dispense 4500 nL to (P2, C7, R1, S1), well volume 0 µL
Change pipettes
Aspirate 4500 nL from (P1, C5, R1, S1), well volume 0 µL, manual height: 0.5 mm
Dispense 4500 nL to (P2, C8, R1, S1), well volume 0 µL
Change pipettes
Aspirate 4500 nL from (P1, C5, R1, S1), well volume 0 µL, manual height: 0.5 mm
Dispense 4500 nL to (P2, C9, R1, S1), well volume 0 µL
Change pipettes
Aspirate 4500 nL from (P1, C5, R1, S1), well volume 0 µL, manual height: 0.5 mm
Dispense 4500 nL to (P2, C10, R1, S1), well volume 0 µL
Change pipettes
Aspirate 4500 nL from (P1, C5, R1, S1), well volume 0 µL, manual height: 0.5 mm
Dispense 4500 nL to (P2, C11, R1, S1), well volume 0 µL
Change pipettes
Aspirate 4500 nL from (P1, C5, R1, S1), well volume 0 µL, manual height: 0.5 mm
Dispense 4500 nL to (P2, C12, R1, S1), well volume 0 µL
Change pipettes
Aspirate 4500 nL from (P1, C6, R1, S1), well volume 0 µL, manual height: 0.5 mm
Dispense 4500 nL to (P2, C13, R1, S1), well volume 0 µL
Change pipettes
Aspirate 4500 nL from (P1, C6, R1, S1), well volume 0 µL, manual height: 0.5 mm
Dispense 4500 nL to (P2, C14, R1, S1), well volume 0 µL
Change pipettes
Aspirate 4500 nL from (P1, C6, R1, S1), well volume 0 µL, manual height: 0.5 mm
Dispense 4500 nL to (P2, C15, R1, S1), well volume 0 µL
Change pipettes
Aspirate 4500 nL from (P1, C6, R1, S1), well volume 0 µL, manual height: 0.5 mm
Dispense 4500 nL to (P2, C16, R1, S1), well volume 0 µL

Change pipettes
Aspirate 4500 nL from (P1, C6, R1, S1), well volume 0 µL, manual height: 0.5 mm
Dispense 4500 nL to (P2, C17, R1, S1), well volume 0 µL
Change pipettes
Aspirate 4500 nL from (P1, C6, R1, S1), well volume 0 µL, manual height: 0.5 mm
Dispense 4500 nL to (P2, C18, R1, S1), well volume 0 µL
Change pipettes
Aspirate 4500 nL from (P1, C6, R1, S1), well volume 0 µL, manual height: 0.5 mm
Dispense 4500 nL to (P2, C19, R1, S1), well volume 0 µL
Change pipettes
Aspirate 4500 nL from (P1, C6, R1, S1), well volume 0 µL, manual height: 0.5 mm
Dispense 4500 nL to (P2, C20, R1, S1), well volume 0 µL
Change pipettes
Aspirate 4500 nL from (P1, C6, R1, S1), well volume 0 µL, manual height: 0.5 mm
Dispense 4500 nL to (P2, C21, R1, S1), well volume 0 µL
Change pipettes
Aspirate 4500 nL from (P1, C6, R1, S1), well volume 0 µL, manual height: 0.5 mm
Dispense 4500 nL to (P2, C22, R1, S1), well volume 0 µL
Change pipettes
Aspirate 4500 nL from (P1, C6, R1, S1), well volume 0 µL, manual height: 0.5 mm
Dispense 4500 nL to (P2, C23, R1, S1), well volume 0 µL
Change pipettes
Aspirate 4500 nL from (P1, C6, R1, S1), well volume 0 µL, manual height: 0.5 mm
Dispense 4500 nL to (P2, C24, R1, S1), well volume 0 µL
Pause indefinitely at position 3 and display message "seal the plate, vortex, cfg, run PCR, cfg" - then home deck
