# Supplementary material for: Single-nucleus RNA-seq2 reveals functional crosstalk between liver zonation and ploidy
Source: Nat Commun. 2021 Jul 12;12:4264. doi: 10.1038/s41467-021-24543-5 (PMC8275628; doi:10.1038/s41467-021-24543-5)
Supplement: Supplementary file 16 — Dataset 14 [file 41467_2021_24543_MOESM16_ESM.pdf]

5 position deck
Position:
1: TTP LVSD; Plate Id: reagents
2: Eppendorf twin.tecÆ 384; Plate Id: reaction
3: Magnetic block- Eppendorf twin.tecÆ 384; Plate Id: magnet
4: TTP LVSD; Plate Id: waste
5: Eppendorf twin.tecÆ 384; Plate Id: purified

Home tape
Aspirate 5000 nL from (P1, C8, R1, S1), well volume 0 µL
Dispense 5000 nL to (P2, C1, R1, S1), well volume 0 µL, manual height: 4 mm
Change pipettes
Aspirate 5000 nL from (P1, C8, R1, S1), well volume 0 µL
Dispense 5000 nL to (P2, C2, R1, S1), well volume 0 µL, manual height: 4 mm
Change pipettes
Aspirate 5000 nL from (P1, C8, R1, S1), well volume 0 µL
Dispense 5000 nL to (P2, C3, R1, S1), well volume 0 µL, manual height: 4 mm
Change pipettes
Aspirate 5000 nL from (P1, C8, R1, S1), well volume 0 µL
Dispense 5000 nL to (P2, C4, R1, S1), well volume 0 µL, manual height: 4 mm
Change pipettes
Aspirate 5000 nL from (P1, C8, R1, S1), well volume 0 µL
Dispense 5000 nL to (P2, C5, R1, S1), well volume 0 µL, manual height: 4 mm
Change pipettes
Aspirate 5000 nL from (P1, C8, R1, S1), well volume 0 µL
Dispense 5000 nL to (P2, C6, R1, S1), well volume 0 µL, manual height: 4 mm
Change pipettes
Aspirate 5000 nL from (P1, C8, R1, S1), well volume 0 µL
Dispense 5000 nL to (P2, C7, R1, S1), well volume 0 µL, manual height: 4 mm
Change pipettes
Aspirate 5000 nL from (P1, C8, R1, S1), well volume 0 µL
Dispense 5000 nL to (P2, C8, R1, S1), well volume 0 µL, manual height: 4 mm
Change pipettes
Aspirate 5000 nL from (P1, C9, R1, S1), well volume 0 µL
Dispense 5000 nL to (P2, C9, R1, S1), well volume 0 µL, manual height: 4 mm
Change pipettes
Aspirate 5000 nL from (P1, C9, R1, S1), well volume 0 µL
Dispense 5000 nL to (P2, C10, R1, S1), well volume 0 µL, manual height: 4 mm
Change pipettes
Aspirate 5000 nL from (P1, C9, R1, S1), well volume 0 µL
Dispense 5000 nL to (P2, C11, R1, S1), well volume 0 µL, manual height: 4 mm
Change pipettes
Aspirate 5000 nL from (P1, C9, R1, S1), well volume 0 µL

Dispense 5000 nL to (P2, C12, R1, S1), well volume 0 µL, manual height: 4 mm
Change pipettes
Aspirate 5000 nL from (P1, C9, R1, S1), well volume 0 µL
Dispense 5000 nL to (P2, C13, R1, S1), well volume 0 µL, manual height: 4 mm
Change pipettes
Aspirate 5000 nL from (P1, C9, R1, S1), well volume 0 µL
Dispense 5000 nL to (P2, C14, R1, S1), well volume 0 µL, manual height: 4 mm
Change pipettes
Aspirate 5000 nL from (P1, C9, R1, S1), well volume 0 µL
Dispense 5000 nL to (P2, C15, R1, S1), well volume 0 µL, manual height: 4 mm
Change pipettes
Aspirate 5000 nL from (P1, C9, R1, S1), well volume 0 µL
Dispense 5000 nL to (P2, C16, R1, S1), well volume 0 µL, manual height: 4 mm
Change pipettes
Aspirate 5000 nL from (P1, C10, R1, S1), well volume 0 µL
Dispense 5000 nL to (P2, C17, R1, S1), well volume 0 µL, manual height: 4 mm
Change pipettes
Aspirate 5000 nL from (P1, C10, R1, S1), well volume 0 µL
Dispense 5000 nL to (P2, C18, R1, S1), well volume 0 µL, manual height: 4 mm
Change pipettes
Aspirate 5000 nL from (P1, C10, R1, S1), well volume 0 µL
Dispense 5000 nL to (P2, C19, R1, S1), well volume 0 µL, manual height: 4 mm
Change pipettes
Aspirate 5000 nL from (P1, C10, R1, S1), well volume 0 µL
Dispense 5000 nL to (P2, C20, R1, S1), well volume 0 µL, manual height: 4 mm
Change pipettes
Aspirate 5000 nL from (P1, C10, R1, S1), well volume 0 µL
Dispense 5000 nL to (P2, C21, R1, S1), well volume 0 µL, manual height: 4 mm
Change pipettes
Aspirate 5000 nL from (P1, C10, R1, S1), well volume 0 µL
Dispense 5000 nL to (P2, C22, R1, S1), well volume 0 µL, manual height: 4 mm
Change pipettes
Aspirate 5000 nL from (P1, C10, R1, S1), well volume 0 µL
Dispense 5000 nL to (P2, C23, R1, S1), well volume 0 µL, manual height: 4 mm
Change pipettes
Aspirate 5000 nL from (P1, C10, R1, S1), well volume 0 µL
Dispense 5000 nL to (P2, C24, R1, S1), well volume 0 µL, manual height: 4 mm
Change pipettes
Pause indefinitely at position 4 and display message "seal the plate, vortex, briefly spin down, incubate 8 min at RT"
Pause indefinitely at position 4 and display message "move plate

onto magnet (position 3), incubate 2 min"
Pause for 120 seconds - then home deck
Aspirate 5000 nL from (P3, C1, R1, S1), well volume 0 µL, piston speed: 3 mm/s
Dispense 5000 nL to (P4, C1, R1, S1), well volume 0 µL
Aspirate 5000 nL from (P3, C1, R1, S1), well volume 0 µL, piston speed: 3 mm/s
Dispense 5000 nL to (P4, C1, R1, S1), well volume 0 µL
Change pipettes
Aspirate 5000 nL from (P3, C2, R1, S1), well volume 0 µL, piston speed: 3 mm/s
Dispense 5000 nL to (P4, C2, R1, S1), well volume 0 µL
Aspirate 5000 nL from (P3, C2, R1, S1), well volume 0 µL, piston speed: 3 mm/s
Dispense 5000 nL to (P4, C2, R1, S1), well volume 0 µL
Change pipettes
Aspirate 5000 nL from (P3, C3, R1, S1), well volume 0 µL, piston speed: 3 mm/s
Dispense 5000 nL to (P4, C3, R1, S1), well volume 0 µL
Aspirate 5000 nL from (P3, C3, R1, S1), well volume 0 µL, piston speed: 3 mm/s
Dispense 5000 nL to (P4, C3, R1, S1), well volume 0 µL
Change pipettes
Aspirate 5000 nL from (P3, C4, R1, S1), well volume 0 µL, piston speed: 3 mm/s
Dispense 5000 nL to (P4, C4, R1, S1), well volume 0 µL
Aspirate 5000 nL from (P3, C4, R1, S1), well volume 0 µL, piston speed: 3 mm/s
Dispense 5000 nL to (P4, C4, R1, S1), well volume 0 µL
Change pipettes
Aspirate 5000 nL from (P3, C5, R1, S1), well volume 0 µL, piston speed: 3 mm/s
Dispense 5000 nL to (P4, C5, R1, S1), well volume 0 µL
Aspirate 5000 nL from (P3, C5, R1, S1), well volume 0 µL, piston speed: 3 mm/s
Dispense 5000 nL to (P4, C5, R1, S1), well volume 0 µL
Change pipettes
Aspirate 5000 nL from (P3, C6, R1, S1), well volume 0 µL, piston speed: 3 mm/s
Dispense 5000 nL to (P4, C6, R1, S1), well volume 0 µL
Aspirate 5000 nL from (P3, C6, R1, S1), well volume 0 µL, piston speed: 3 mm/s
Dispense 5000 nL to (P4, C6, R1, S1), well volume 0 µL
Change pipettes
Aspirate 5000 nL from (P3, C7, R1, S1), well volume 0 µL, piston speed: 3 mm/s
Dispense 5000 nL to (P4, C7, R1, S1), well volume 0 µL
Aspirate 5000 nL from (P3, C7, R1, S1), well volume 0 µL, piston speed: 3 mm/s
Dispense 5000 nL to (P4, C7, R1, S1), well volume 0 µL
Change pipettes
Aspirate 5000 nL from (P3, C8, R1, S1), well volume 0 µL, piston speed: 3 mm/s
Dispense 5000 nL to (P4, C8, R1, S1), well volume 0 µL

Aspirate 5000 nL from (P3, C8, R1, S1), well volume 0 µL, piston speed: 3 mm/s
Dispense 5000 nL to (P4, C8, R1, S1), well volume 0 µL
Change pipettes
Aspirate 5000 nL from (P3, C9, R1, S1), well volume 0 µL, piston speed: 3 mm/s
Dispense 5000 nL to (P4, C9, R1, S1), well volume 0 µL
Aspirate 5000 nL from (P3, C9, R1, S1), well volume 0 µL, piston speed: 3 mm/s
Dispense 5000 nL to (P4, C9, R1, S1), well volume 0 µL
Change pipettes
Aspirate 5000 nL from (P3, C10, R1, S1), well volume 0 µL, piston speed: 3 mm/s
Dispense 5000 nL to (P4, C10, R1, S1), well volume 0 µL
Aspirate 5000 nL from (P3, C10, R1, S1), well volume 0 µL, piston speed: 3 mm/s
Dispense 5000 nL to (P4, C10, R1, S1), well volume 0 µL
Change pipettes
Aspirate 5000 nL from (P3, C11, R1, S1), well volume 0 µL, piston speed: 3 mm/s
Dispense 5000 nL to (P4, C11, R1, S1), well volume 0 µL
Aspirate 5000 nL from (P3, C11, R1, S1), well volume 0 µL, piston speed: 3 mm/s
Dispense 5000 nL to (P4, C11, R1, S1), well volume 0 µL
Change pipettes
Aspirate 5000 nL from (P3, C12, R1, S1), well volume 0 µL, piston speed: 3 mm/s
Dispense 5000 nL to (P4, C12, R1, S1), well volume 0 µL
Aspirate 5000 nL from (P3, C12, R1, S1), well volume 0 µL, piston speed: 3 mm/s
Dispense 5000 nL to (P4, C12, R1, S1), well volume 0 µL
Change pipettes
Aspirate 5000 nL from (P3, C13, R1, S1), well volume 0 µL, piston speed: 3 mm/s
Dispense 5000 nL to (P4, C13, R1, S1), well volume 0 µL
Aspirate 5000 nL from (P3, C13, R1, S1), well volume 0 µL, piston speed: 3 mm/s
Dispense 5000 nL to (P4, C13, R1, S1), well volume 0 µL
Change pipettes
Aspirate 5000 nL from (P3, C14, R1, S1), well volume 0 µL, piston speed: 3 mm/s
Dispense 5000 nL to (P4, C14, R1, S1), well volume 0 µL
Aspirate 5000 nL from (P3, C14, R1, S1), well volume 0 µL, piston speed: 3 mm/s
Dispense 5000 nL to (P4, C14, R1, S1), well volume 0 µL
Change pipettes
Aspirate 5000 nL from (P3, C15, R1, S1), well volume 0 µL, piston speed: 3 mm/s
Dispense 5000 nL to (P4, C15, R1, S1), well volume 0 µL
Aspirate 5000 nL from (P3, C15, R1, S1), well volume 0 µL, piston speed: 3 mm/s
Dispense 5000 nL to (P4, C15, R1, S1), well volume 0 µL
Change pipettes
Aspirate 5000 nL from (P3, C16, R1, S1), well volume 0 µL, piston

speed: 3 mm/s
Dispense 5000 nL to (P4, C16, R1, S1), well volume 0 µL
Aspirate 5000 nL from (P3, C16, R1, S1), well volume 0 µL, piston speed: 3 mm/s
Dispense 5000 nL to (P4, C16, R1, S1), well volume 0 µL
Change pipettes
Aspirate 5000 nL from (P3, C17, R1, S1), well volume 0 µL, piston speed: 3 mm/s
Dispense 5000 nL to (P4, C17, R1, S1), well volume 0 µL
Aspirate 5000 nL from (P3, C17, R1, S1), well volume 0 µL, piston speed: 3 mm/s
Dispense 5000 nL to (P4, C17, R1, S1), well volume 0 µL
Change pipettes
Aspirate 5000 nL from (P3, C18, R1, S1), well volume 0 µL, piston speed: 3 mm/s
Dispense 5000 nL to (P4, C18, R1, S1), well volume 0 µL
Aspirate 5000 nL from (P3, C18, R1, S1), well volume 0 µL, piston speed: 3 mm/s
Dispense 5000 nL to (P4, C18, R1, S1), well volume 0 µL
Change pipettes
Aspirate 5000 nL from (P3, C19, R1, S1), well volume 0 µL, piston speed: 3 mm/s
Dispense 5000 nL to (P4, C19, R1, S1), well volume 0 µL
Aspirate 5000 nL from (P3, C19, R1, S1), well volume 0 µL, piston speed: 3 mm/s
Dispense 5000 nL to (P4, C19, R1, S1), well volume 0 µL
Change pipettes
Aspirate 5000 nL from (P3, C20, R1, S1), well volume 0 µL, piston speed: 3 mm/s
Dispense 5000 nL to (P4, C20, R1, S1), well volume 0 µL
Aspirate 5000 nL from (P3, C20, R1, S1), well volume 0 µL, piston speed: 3 mm/s
Dispense 5000 nL to (P4, C20, R1, S1), well volume 0 µL
Change pipettes
Aspirate 5000 nL from (P3, C21, R1, S1), well volume 0 µL, piston speed: 3 mm/s
Dispense 5000 nL to (P4, C21, R1, S1), well volume 0 µL
Aspirate 5000 nL from (P3, C21, R1, S1), well volume 0 µL, piston speed: 3 mm/s
Dispense 5000 nL to (P4, C21, R1, S1), well volume 0 µL
Change pipettes
Aspirate 5000 nL from (P3, C22, R1, S1), well volume 0 µL, piston speed: 3 mm/s
Dispense 5000 nL to (P4, C22, R1, S1), well volume 0 µL
Aspirate 5000 nL from (P3, C22, R1, S1), well volume 0 µL, piston speed: 3 mm/s
Dispense 5000 nL to (P4, C22, R1, S1), well volume 0 µL
Change pipettes
Aspirate 5000 nL from (P3, C23, R1, S1), well volume 0 µL, piston speed: 3 mm/s
Dispense 5000 nL to (P4, C23, R1, S1), well volume 0 µL
Aspirate 5000 nL from (P3, C23, R1, S1), well volume 0 µL, piston speed: 3 mm/s
Dispense 5000 nL to (P4, C23, R1, S1), well volume 0 µL

Change pipettes
Aspirate 5000 nL from (P3, C24, R1, S1), well volume 0 µL, piston speed: 3 mm/s
Dispense 5000 nL to (P4, C24, R1, S1), well volume 0 µL
Aspirate 5000 nL from (P3, C24, R1, S1), well volume 0 µL, piston speed: 3 mm/s
Dispense 5000 nL to (P4, C24, R1, S1), well volume 0 µL
Change pipettes
Aspirate 4500 nL from (P1, C12, R1, S1), well volume 0 µL
Dispense 4500 nL to (P3, C1, R1, S1), well volume 0 µL, piston speed: 10 mm/s
Change pipettes
Aspirate 4500 nL from (P1, C12, R1, S1), well volume 0 µL
Dispense 4500 nL to (P3, C2, R1, S1), well volume 0 µL, piston speed: 10 mm/s
Change pipettes
Aspirate 4500 nL from (P1, C12, R1, S1), well volume 0 µL
Dispense 4500 nL to (P3, C3, R1, S1), well volume 0 µL, piston speed: 10 mm/s
Change pipettes
Aspirate 4500 nL from (P1, C12, R1, S1), well volume 0 µL
Dispense 4500 nL to (P3, C4, R1, S1), well volume 0 µL, piston speed: 10 mm/s
Change pipettes
Aspirate 4500 nL from (P1, C12, R1, S1), well volume 0 µL
Dispense 4500 nL to (P3, C5, R1, S1), well volume 0 µL, piston speed: 10 mm/s
Change pipettes
Aspirate 4500 nL from (P1, C12, R1, S1), well volume 0 µL
Dispense 4500 nL to (P3, C6, R1, S1), well volume 0 µL, piston speed: 10 mm/s
Change pipettes
Aspirate 4500 nL from (P1, C12, R1, S1), well volume 0 µL
Dispense 4500 nL to (P3, C7, R1, S1), well volume 0 µL, piston speed: 10 mm/s
Change pipettes
Aspirate 4500 nL from (P1, C12, R1, S1), well volume 0 µL
Dispense 4500 nL to (P3, C8, R1, S1), well volume 0 µL, piston speed: 10 mm/s
Change pipettes
Aspirate 4500 nL from (P1, C13, R1, S1), well volume 0 µL
Dispense 4500 nL to (P3, C9, R1, S1), well volume 0 µL, piston speed: 10 mm/s
Change pipettes
Aspirate 4500 nL from (P1, C13, R1, S1), well volume 0 µL
Dispense 4500 nL to (P3, C10, R1, S1), well volume 0 µL, piston speed: 10 mm/s
Change pipettes
Aspirate 4500 nL from (P1, C13, R1, S1), well volume 0 µL
Dispense 4500 nL to (P3, C11, R1, S1), well volume 0 µL, piston speed: 10 mm/s
Change pipettes
Aspirate 4500 nL from (P1, C13, R1, S1), well volume 0 µL
Dispense 4500 nL to (P3, C12, R1, S1), well volume 0 µL, piston

speed: 10 mm/s
Change pipettes
Aspirate 4500 nL from (P1, C13, R1, S1), well volume 0 µL
Dispense 4500 nL to (P3, C13, R1, S1), well volume 0 µL, piston speed: 10 mm/s
Change pipettes
Aspirate 4500 nL from (P1, C13, R1, S1), well volume 0 µL
Dispense 4500 nL to (P3, C14, R1, S1), well volume 0 µL, piston speed: 10 mm/s
Change pipettes
Aspirate 4500 nL from (P1, C13, R1, S1), well volume 0 µL
Dispense 4500 nL to (P3, C15, R1, S1), well volume 0 µL, piston speed: 10 mm/s
Change pipettes
Aspirate 4500 nL from (P1, C13, R1, S1), well volume 0 µL
Dispense 4500 nL to (P3, C16, R1, S1), well volume 0 µL, piston speed: 10 mm/s
Change pipettes
Aspirate 4500 nL from (P1, C14, R1, S1), well volume 0 µL
Dispense 4500 nL to (P3, C17, R1, S1), well volume 0 µL, piston speed: 10 mm/s
Change pipettes
Aspirate 4500 nL from (P1, C14, R1, S1), well volume 0 µL
Dispense 4500 nL to (P3, C18, R1, S1), well volume 0 µL, piston speed: 10 mm/s
Change pipettes
Aspirate 4500 nL from (P1, C14, R1, S1), well volume 0 µL
Dispense 4500 nL to (P3, C19, R1, S1), well volume 0 µL, piston speed: 10 mm/s
Change pipettes
Aspirate 4500 nL from (P1, C14, R1, S1), well volume 0 µL
Dispense 4500 nL to (P3, C20, R1, S1), well volume 0 µL, piston speed: 10 mm/s
Change pipettes
Aspirate 4500 nL from (P1, C14, R1, S1), well volume 0 µL
Dispense 4500 nL to (P3, C21, R1, S1), well volume 0 µL, piston speed: 10 mm/s
Change pipettes
Aspirate 4500 nL from (P1, C14, R1, S1), well volume 0 µL
Dispense 4500 nL to (P3, C22, R1, S1), well volume 0 µL, piston speed: 10 mm/s
Change pipettes
Aspirate 4500 nL from (P1, C14, R1, S1), well volume 0 µL
Dispense 4500 nL to (P3, C23, R1, S1), well volume 0 µL, piston speed: 10 mm/s
Change pipettes
Aspirate 4500 nL from (P1, C14, R1, S1), well volume 0 µL
Dispense 4500 nL to (P3, C24, R1, S1), well volume 0 µL, piston speed: 10 mm/s
Change pipettes
Aspirate 5000 nL from (P3, C1, R1, S1), well volume 0 µL, manual height: 0.5 mm, piston speed: 3 mm/s
Dispense 5000 nL to (P4, C1, R1, S1), well volume 0 µL
Change pipettes

Aspirate 5000 nL from (P3, C2, R1, S1), well volume 0 µL, manual height: 0.5 mm, piston speed: 3 mm/s
Dispense 5000 nL to (P4, C2, R1, S1), well volume 0 µL
Change pipettes
Aspirate 5000 nL from (P3, C3, R1, S1), well volume 0 µL, manual height: 0.5 mm, piston speed: 3 mm/s
Dispense 5000 nL to (P4, C3, R1, S1), well volume 0 µL
Change pipettes
Aspirate 5000 nL from (P3, C4, R1, S1), well volume 0 µL, manual height: 0.5 mm, piston speed: 3 mm/s
Dispense 5000 nL to (P4, C4, R1, S1), well volume 0 µL
Change pipettes
Aspirate 5000 nL from (P3, C5, R1, S1), well volume 0 µL, manual height: 0.5 mm, piston speed: 3 mm/s
Dispense 5000 nL to (P4, C5, R1, S1), well volume 0 µL
Change pipettes
Aspirate 5000 nL from (P3, C6, R1, S1), well volume 0 µL, manual height: 0.5 mm, piston speed: 3 mm/s
Dispense 5000 nL to (P4, C6, R1, S1), well volume 0 µL
Change pipettes
Aspirate 5000 nL from (P3, C7, R1, S1), well volume 0 µL, manual height: 0.5 mm, piston speed: 3 mm/s
Dispense 5000 nL to (P4, C7, R1, S1), well volume 0 µL
Change pipettes
Aspirate 5000 nL from (P3, C8, R1, S1), well volume 0 µL, manual height: 0.5 mm, piston speed: 3 mm/s
Dispense 5000 nL to (P4, C8, R1, S1), well volume 0 µL
Change pipettes
Aspirate 5000 nL from (P3, C9, R1, S1), well volume 0 µL, manual height: 0.5 mm, piston speed: 3 mm/s
Dispense 5000 nL to (P4, C9, R1, S1), well volume 0 µL
Change pipettes
Aspirate 5000 nL from (P3, C10, R1, S1), well volume 0 µL, manual height: 0.5 mm, piston speed: 3 mm/s
Dispense 5000 nL to (P4, C10, R1, S1), well volume 0 µL
Change pipettes
Aspirate 5000 nL from (P3, C11, R1, S1), well volume 0 µL, manual height: 0.5 mm, piston speed: 3 mm/s
Dispense 5000 nL to (P4, C11, R1, S1), well volume 0 µL
Change pipettes
Aspirate 5000 nL from (P3, C12, R1, S1), well volume 0 µL, manual height: 0.5 mm, piston speed: 3 mm/s
Dispense 5000 nL to (P4, C12, R1, S1), well volume 0 µL
Change pipettes
Aspirate 5000 nL from (P3, C13, R1, S1), well volume 0 µL, manual height: 0.5 mm, piston speed: 3 mm/s
Dispense 5000 nL to (P4, C13, R1, S1), well volume 0 µL
Change pipettes
Aspirate 5000 nL from (P3, C14, R1, S1), well volume 0 µL, manual height: 0.5 mm, piston speed: 3 mm/s
Dispense 5000 nL to (P4, C14, R1, S1), well volume 0 µL
Change pipettes
Aspirate 5000 nL from (P3, C15, R1, S1), well volume 0 µL, manual height: 0.5 mm, piston speed: 3 mm/s

Dispense 5000 nL to (P4, C15, R1, S1), well volume 0 µL
Change pipettes
Aspirate 5000 nL from (P3, C16, R1, S1), well volume 0 µL, manual height: 0.5 mm, piston speed: 3 mm/s
Dispense 5000 nL to (P4, C16, R1, S1), well volume 0 µL
Change pipettes
Aspirate 5000 nL from (P3, C17, R1, S1), well volume 0 µL, manual height: 0.5 mm, piston speed: 3 mm/s
Dispense 5000 nL to (P4, C17, R1, S1), well volume 0 µL
Change pipettes
Aspirate 5000 nL from (P3, C18, R1, S1), well volume 0 µL, manual height: 0.5 mm, piston speed: 3 mm/s
Dispense 5000 nL to (P4, C18, R1, S1), well volume 0 µL
Change pipettes
Aspirate 5000 nL from (P3, C19, R1, S1), well volume 0 µL, manual height: 0.5 mm, piston speed: 3 mm/s
Dispense 5000 nL to (P4, C19, R1, S1), well volume 0 µL
Change pipettes
Aspirate 5000 nL from (P3, C20, R1, S1), well volume 0 µL, manual height: 0.5 mm, piston speed: 3 mm/s
Dispense 5000 nL to (P4, C20, R1, S1), well volume 0 µL
Change pipettes
Aspirate 5000 nL from (P3, C21, R1, S1), well volume 0 µL, manual height: 0.5 mm, piston speed: 3 mm/s
Dispense 5000 nL to (P4, C21, R1, S1), well volume 0 µL
Change pipettes
Aspirate 5000 nL from (P3, C22, R1, S1), well volume 0 µL, manual height: 0.5 mm, piston speed: 3 mm/s
Dispense 5000 nL to (P4, C22, R1, S1), well volume 0 µL
Change pipettes
Aspirate 5000 nL from (P3, C23, R1, S1), well volume 0 µL, manual height: 0.5 mm, piston speed: 3 mm/s
Dispense 5000 nL to (P4, C23, R1, S1), well volume 0 µL
Change pipettes
Aspirate 5000 nL from (P3, C24, R1, S1), well volume 0 µL, manual height: 0.5 mm, piston speed: 3 mm/s
Dispense 5000 nL to (P4, C24, R1, S1), well volume 0 µL
Change pipettes
Aspirate 4500 nL from (P1, C15, R1, S1), well volume 0 µL
Dispense 4500 nL to (P3, C1, R1, S1), well volume 0 µL, piston speed: 10 mm/s
Change pipettes
Aspirate 4500 nL from (P1, C15, R1, S1), well volume 0 µL
Dispense 4500 nL to (P3, C2, R1, S1), well volume 0 µL, piston speed: 10 mm/s
Change pipettes
Aspirate 4500 nL from (P1, C15, R1, S1), well volume 0 µL
Dispense 4500 nL to (P3, C3, R1, S1), well volume 0 µL, piston speed: 10 mm/s
Change pipettes
Aspirate 4500 nL from (P1, C15, R1, S1), well volume 0 µL
Dispense 4500 nL to (P3, C4, R1, S1), well volume 0 µL, piston speed: 10 mm/s
Change pipettes

Aspirate 4500 nL from (P1, C15, R1, S1), well volume 0 µL
Dispense 4500 nL to (P3, C5, R1, S1), well volume 0 µL, piston speed: 10 mm/s
Change pipettes
Aspirate 4500 nL from (P1, C15, R1, S1), well volume 0 µL
Dispense 4500 nL to (P3, C6, R1, S1), well volume 0 µL, piston speed: 10 mm/s
Change pipettes
Aspirate 4500 nL from (P1, C15, R1, S1), well volume 0 µL
Dispense 4500 nL to (P3, C7, R1, S1), well volume 0 µL, piston speed: 10 mm/s
Change pipettes
Aspirate 4500 nL from (P1, C15, R1, S1), well volume 0 µL
Dispense 4500 nL to (P3, C8, R1, S1), well volume 0 µL, piston speed: 10 mm/s
Change pipettes
Aspirate 4500 nL from (P1, C16, R1, S1), well volume 0 µL
Dispense 4500 nL to (P3, C9, R1, S1), well volume 0 µL, piston speed: 10 mm/s
Change pipettes
Aspirate 4500 nL from (P1, C16, R1, S1), well volume 0 µL
Dispense 4500 nL to (P3, C10, R1, S1), well volume 0 µL, piston speed: 10 mm/s
Change pipettes
Aspirate 4500 nL from (P1, C16, R1, S1), well volume 0 µL
Dispense 4500 nL to (P3, C11, R1, S1), well volume 0 µL, piston speed: 10 mm/s
Change pipettes
Aspirate 4500 nL from (P1, C16, R1, S1), well volume 0 µL
Dispense 4500 nL to (P3, C12, R1, S1), well volume 0 µL, piston speed: 10 mm/s
Change pipettes
Aspirate 4500 nL from (P1, C16, R1, S1), well volume 0 µL
Dispense 4500 nL to (P3, C13, R1, S1), well volume 0 µL, piston speed: 10 mm/s
Change pipettes
Aspirate 4500 nL from (P1, C16, R1, S1), well volume 0 µL
Dispense 4500 nL to (P3, C14, R1, S1), well volume 0 µL, piston speed: 10 mm/s
Change pipettes
Aspirate 4500 nL from (P1, C16, R1, S1), well volume 0 µL
Dispense 4500 nL to (P3, C15, R1, S1), well volume 0 µL, piston speed: 10 mm/s
Change pipettes
Aspirate 4500 nL from (P1, C16, R1, S1), well volume 0 µL
Dispense 4500 nL to (P3, C16, R1, S1), well volume 0 µL, piston speed: 10 mm/s
Change pipettes
Aspirate 4500 nL from (P1, C17, R1, S1), well volume 0 µL
Dispense 4500 nL to (P3, C17, R1, S1), well volume 0 µL, piston speed: 10 mm/s
Change pipettes
Aspirate 4500 nL from (P1, C17, R1, S1), well volume 0 µL
Dispense 4500 nL to (P3, C18, R1, S1), well volume 0 µL, piston

speed: 10 mm/s
Change pipettes
Aspirate 4500 nL from (P1, C17, R1, S1), well volume 0 µL
Dispense 4500 nL to (P3, C19, R1, S1), well volume 0 µL, piston speed: 10 mm/s
Change pipettes
Aspirate 4500 nL from (P1, C17, R1, S1), well volume 0 µL
Dispense 4500 nL to (P3, C20, R1, S1), well volume 0 µL, piston speed: 10 mm/s
Change pipettes
Aspirate 4500 nL from (P1, C17, R1, S1), well volume 0 µL
Dispense 4500 nL to (P3, C21, R1, S1), well volume 0 µL, piston speed: 10 mm/s
Change pipettes
Aspirate 4500 nL from (P1, C17, R1, S1), well volume 0 µL
Dispense 4500 nL to (P3, C22, R1, S1), well volume 0 µL, piston speed: 10 mm/s
Change pipettes
Aspirate 4500 nL from (P1, C17, R1, S1), well volume 0 µL
Dispense 4500 nL to (P3, C23, R1, S1), well volume 0 µL, piston speed: 10 mm/s
Change pipettes
Aspirate 4500 nL from (P1, C17, R1, S1), well volume 0 µL
Dispense 4500 nL to (P3, C24, R1, S1), well volume 0 µL, piston speed: 10 mm/s
Change pipettes
Aspirate 5000 nL from (P3, C1, R1, S1), well volume 0 µL, manual height: 0.3 mm, piston speed: 3 mm/s
Dispense 5000 nL to (P4, C1, R1, S1), well volume 0 µL
Change pipettes
Aspirate 5000 nL from (P3, C2, R1, S1), well volume 0 µL, manual height: 0.3 mm, piston speed: 3 mm/s
Dispense 5000 nL to (P4, C2, R1, S1), well volume 0 µL
Change pipettes
Aspirate 5000 nL from (P3, C3, R1, S1), well volume 0 µL, manual height: 0.3 mm, piston speed: 3 mm/s
Dispense 5000 nL to (P4, C3, R1, S1), well volume 0 µL
Change pipettes
Aspirate 5000 nL from (P3, C4, R1, S1), well volume 0 µL, manual height: 0.3 mm, piston speed: 3 mm/s
Dispense 5000 nL to (P4, C4, R1, S1), well volume 0 µL
Change pipettes
Aspirate 5000 nL from (P3, C5, R1, S1), well volume 0 µL, manual height: 0.3 mm, piston speed: 3 mm/s
Dispense 5000 nL to (P4, C5, R1, S1), well volume 0 µL
Change pipettes
Aspirate 5000 nL from (P3, C6, R1, S1), well volume 0 µL, manual height: 0.3 mm, piston speed: 3 mm/s
Dispense 5000 nL to (P4, C6, R1, S1), well volume 0 µL
Change pipettes
Aspirate 5000 nL from (P3, C7, R1, S1), well volume 0 µL, manual height: 0.3 mm, piston speed: 3 mm/s
Dispense 5000 nL to (P4, C7, R1, S1), well volume 0 µL
Change pipettes

Aspirate 5000 nL from (P3, C8, R1, S1), well volume 0 µL, manual height: 0.3 mm, piston speed: 3 mm/s
Dispense 5000 nL to (P4, C8, R1, S1), well volume 0 µL
Change pipettes
Aspirate 5000 nL from (P3, C9, R1, S1), well volume 0 µL, manual height: 0.3 mm, piston speed: 3 mm/s
Dispense 5000 nL to (P4, C9, R1, S1), well volume 0 µL
Change pipettes
Aspirate 5000 nL from (P3, C10, R1, S1), well volume 0 µL, manual height: 0.3 mm, piston speed: 3 mm/s
Dispense 5000 nL to (P4, C10, R1, S1), well volume 0 µL
Change pipettes
Aspirate 5000 nL from (P3, C11, R1, S1), well volume 0 µL, manual height: 0.3 mm, piston speed: 3 mm/s
Dispense 5000 nL to (P4, C11, R1, S1), well volume 0 µL
Change pipettes
Aspirate 5000 nL from (P3, C12, R1, S1), well volume 0 µL, manual height: 0.3 mm, piston speed: 3 mm/s
Dispense 5000 nL to (P4, C12, R1, S1), well volume 0 µL
Change pipettes
Aspirate 5000 nL from (P3, C13, R1, S1), well volume 0 µL, manual height: 0.3 mm, piston speed: 3 mm/s
Dispense 5000 nL to (P4, C13, R1, S1), well volume 0 µL
Change pipettes
Aspirate 5000 nL from (P3, C14, R1, S1), well volume 0 µL, manual height: 0.3 mm, piston speed: 3 mm/s
Dispense 5000 nL to (P4, C14, R1, S1), well volume 0 µL
Change pipettes
Aspirate 5000 nL from (P3, C15, R1, S1), well volume 0 µL, manual height: 0.3 mm, piston speed: 3 mm/s
Dispense 5000 nL to (P4, C15, R1, S1), well volume 0 µL
Change pipettes
Aspirate 5000 nL from (P3, C16, R1, S1), well volume 0 µL, manual height: 0.3 mm, piston speed: 3 mm/s
Dispense 5000 nL to (P4, C16, R1, S1), well volume 0 µL
Change pipettes
Aspirate 5000 nL from (P3, C17, R1, S1), well volume 0 µL, manual height: 0.3 mm, piston speed: 3 mm/s
Dispense 5000 nL to (P4, C17, R1, S1), well volume 0 µL
Change pipettes
Aspirate 5000 nL from (P3, C18, R1, S1), well volume 0 µL, manual height: 0.3 mm, piston speed: 3 mm/s
Dispense 5000 nL to (P4, C18, R1, S1), well volume 0 µL
Change pipettes
Aspirate 5000 nL from (P3, C19, R1, S1), well volume 0 µL, manual height: 0.3 mm, piston speed: 3 mm/s
Dispense 5000 nL to (P4, C19, R1, S1), well volume 0 µL
Change pipettes
Aspirate 5000 nL from (P3, C20, R1, S1), well volume 0 µL, manual height: 0.3 mm, piston speed: 3 mm/s
Dispense 5000 nL to (P4, C20, R1, S1), well volume 0 µL
Change pipettes
Aspirate 5000 nL from (P3, C21, R1, S1), well volume 0 µL, manual height: 0.3 mm, piston speed: 3 mm/s

Dispense 5000 nL to (P4, C21, R1, S1), well volume 0 µL
Change pipettes
Aspirate 5000 nL from (P3, C22, R1, S1), well volume 0 µL, manual height: 0.3 mm, piston speed: 3 mm/s
Dispense 5000 nL to (P4, C22, R1, S1), well volume 0 µL
Change pipettes
Aspirate 5000 nL from (P3, C23, R1, S1), well volume 0 µL, manual height: 0.3 mm, piston speed: 3 mm/s
Dispense 5000 nL to (P4, C23, R1, S1), well volume 0 µL
Change pipettes
Aspirate 5000 nL from (P3, C24, R1, S1), well volume 0 µL, manual height: 0.3 mm, piston speed: 3 mm/s
Dispense 5000 nL to (P4, C24, R1, S1), well volume 0 µL
Change pipettes
Pause indefinitely at position 4 and display message "air dry, put plate in position 2 for elution" - then home deck
Aspirate 5000 nL from (P1, C19, R1, S1), well volume 0 µL
Dispense 5000 nL to (P2, C1, R1, S1), well volume 0 µL
Change pipettes
Aspirate 5000 nL from (P1, C19, R1, S1), well volume 0 µL
Dispense 5000 nL to (P2, C2, R1, S1), well volume 0 µL
Change pipettes
Aspirate 5000 nL from (P1, C19, R1, S1), well volume 0 µL
Dispense 5000 nL to (P2, C3, R1, S1), well volume 0 µL
Change pipettes
Aspirate 5000 nL from (P1, C19, R1, S1), well volume 0 µL
Dispense 5000 nL to (P2, C4, R1, S1), well volume 0 µL
Change pipettes
Aspirate 5000 nL from (P1, C19, R1, S1), well volume 0 µL
Dispense 5000 nL to (P2, C5, R1, S1), well volume 0 µL
Change pipettes
Aspirate 5000 nL from (P1, C19, R1, S1), well volume 0 µL
Dispense 5000 nL to (P2, C6, R1, S1), well volume 0 µL
Change pipettes
Aspirate 5000 nL from (P1, C19, R1, S1), well volume 0 µL
Dispense 5000 nL to (P2, C7, R1, S1), well volume 0 µL
Change pipettes
Aspirate 5000 nL from (P1, C19, R1, S1), well volume 0 µL
Dispense 5000 nL to (P2, C8, R1, S1), well volume 0 µL
Change pipettes
Aspirate 5000 nL from (P1, C20, R1, S1), well volume 0 µL
Dispense 5000 nL to (P2, C9, R1, S1), well volume 0 µL
Change pipettes
Aspirate 5000 nL from (P1, C20, R1, S1), well volume 0 µL
Dispense 5000 nL to (P2, C10, R1, S1), well volume 0 µL
Change pipettes
Aspirate 5000 nL from (P1, C20, R1, S1), well volume 0 µL
Dispense 5000 nL to (P2, C11, R1, S1), well volume 0 µL
Change pipettes
Aspirate 5000 nL from (P1, C20, R1, S1), well volume 0 µL
Dispense 5000 nL to (P2, C12, R1, S1), well volume 0 µL
Change pipettes
Aspirate 5000 nL from (P1, C20, R1, S1), well volume 0 µL
Dispense 5000 nL to (P2, C13, R1, S1), well volume 0 µL

Change pipettes
Aspirate 5000 nL from (P1, C20, R1, S1), well volume 0 µL
Dispense 5000 nL to (P2, C14, R1, S1), well volume 0 µL
Change pipettes
Aspirate 5000 nL from (P1, C20, R1, S1), well volume 0 µL
Dispense 5000 nL to (P2, C15, R1, S1), well volume 0 µL
Change pipettes
Aspirate 5000 nL from (P1, C20, R1, S1), well volume 0 µL
Dispense 5000 nL to (P2, C16, R1, S1), well volume 0 µL
Change pipettes
Aspirate 5000 nL from (P1, C21, R1, S1), well volume 0 µL
Dispense 5000 nL to (P2, C17, R1, S1), well volume 0 µL
Change pipettes
Aspirate 5000 nL from (P1, C21, R1, S1), well volume 0 µL
Dispense 5000 nL to (P2, C18, R1, S1), well volume 0 µL
Change pipettes
Aspirate 5000 nL from (P1, C21, R1, S1), well volume 0 µL
Dispense 5000 nL to (P2, C19, R1, S1), well volume 0 µL
Change pipettes
Aspirate 5000 nL from (P1, C21, R1, S1), well volume 0 µL
Dispense 5000 nL to (P2, C20, R1, S1), well volume 0 µL
Change pipettes
Aspirate 5000 nL from (P1, C21, R1, S1), well volume 0 µL
Dispense 5000 nL to (P2, C21, R1, S1), well volume 0 µL
Change pipettes
Aspirate 5000 nL from (P1, C21, R1, S1), well volume 0 µL
Dispense 5000 nL to (P2, C22, R1, S1), well volume 0 µL
Change pipettes
Aspirate 5000 nL from (P1, C21, R1, S1), well volume 0 µL
Dispense 5000 nL to (P2, C23, R1, S1), well volume 0 µL
Change pipettes
Aspirate 5000 nL from (P1, C21, R1, S1), well volume 0 µL
Dispense 5000 nL to (P2, C24, R1, S1), well volume 0 µL
Change pipettes
Pause indefinitely at position 4 and display message "seal the plate, vortex, briefly spin down, incubate 2–3 min at RT"
Pause indefinitely at position 4 and display message "move plate onto magnet (position 3), incubate 2 min"
Pause for 120 seconds - then home deck
Aspirate 5000 nL from (P3, C1, R1, S1), well volume 0 µL, manual height: 0.5 mm, piston speed: 3 mm/s
Dispense 5000 nL to (P5, C1, R1, S1), well volume 0 µL
Change pipettes
Aspirate 5000 nL from (P3, C2, R1, S1), well volume 0 µL, manual height: 0.5 mm, piston speed: 3 mm/s
Dispense 5000 nL to (P5, C2, R1, S1), well volume 0 µL
Change pipettes
Aspirate 5000 nL from (P3, C3, R1, S1), well volume 0 µL, manual height: 0.5 mm, piston speed: 3 mm/s
Dispense 5000 nL to (P5, C3, R1, S1), well volume 0 µL
Change pipettes
Aspirate 5000 nL from (P3, C4, R1, S1), well volume 0 µL, manual height: 0.5 mm, piston speed: 3 mm/s
Dispense 5000 nL to (P5, C4, R1, S1), well volume 0 µL

Change pipettes
Aspirate 5000 nL from (P3, C5, R1, S1), well volume 0 µL, manual height: 0.5 mm, piston speed: 3 mm/s
Dispense 5000 nL to (P5, C5, R1, S1), well volume 0 µL
Change pipettes
Aspirate 5000 nL from (P3, C6, R1, S1), well volume 0 µL, manual height: 0.5 mm, piston speed: 3 mm/s
Dispense 5000 nL to (P5, C6, R1, S1), well volume 0 µL
Change pipettes
Aspirate 5000 nL from (P3, C7, R1, S1), well volume 0 µL, manual height: 0.5 mm, piston speed: 3 mm/s
Dispense 5000 nL to (P5, C7, R1, S1), well volume 0 µL
Change pipettes
Aspirate 5000 nL from (P3, C8, R1, S1), well volume 0 µL, manual height: 0.5 mm, piston speed: 3 mm/s
Dispense 5000 nL to (P5, C8, R1, S1), well volume 0 µL
Change pipettes
Aspirate 5000 nL from (P3, C9, R1, S1), well volume 0 µL, manual height: 0.5 mm, piston speed: 3 mm/s
Dispense 5000 nL to (P5, C9, R1, S1), well volume 0 µL
Change pipettes
Aspirate 5000 nL from (P3, C10, R1, S1), well volume 0 µL, manual height: 0.5 mm, piston speed: 3 mm/s
Dispense 5000 nL to (P5, C10, R1, S1), well volume 0 µL
Change pipettes
Aspirate 5000 nL from (P3, C11, R1, S1), well volume 0 µL, manual height: 0.5 mm, piston speed: 3 mm/s
Dispense 5000 nL to (P5, C11, R1, S1), well volume 0 µL
Change pipettes
Aspirate 5000 nL from (P3, C12, R1, S1), well volume 0 µL, manual height: 0.5 mm, piston speed: 3 mm/s
Dispense 5000 nL to (P5, C12, R1, S1), well volume 0 µL
Change pipettes
Aspirate 5000 nL from (P3, C13, R1, S1), well volume 0 µL, manual height: 0.5 mm, piston speed: 3 mm/s
Dispense 5000 nL to (P5, C13, R1, S1), well volume 0 µL
Change pipettes
Aspirate 5000 nL from (P3, C14, R1, S1), well volume 0 µL, manual height: 0.5 mm, piston speed: 3 mm/s
Dispense 5000 nL to (P5, C14, R1, S1), well volume 0 µL
Change pipettes
Aspirate 5000 nL from (P3, C15, R1, S1), well volume 0 µL, manual height: 0.5 mm, piston speed: 3 mm/s
Dispense 5000 nL to (P5, C15, R1, S1), well volume 0 µL
Change pipettes
Aspirate 5000 nL from (P3, C16, R1, S1), well volume 0 µL, manual height: 0.5 mm, piston speed: 3 mm/s
Dispense 5000 nL to (P5, C16, R1, S1), well volume 0 µL
Change pipettes
Aspirate 5000 nL from (P3, C17, R1, S1), well volume 0 µL, manual height: 0.5 mm, piston speed: 3 mm/s
Dispense 5000 nL to (P5, C17, R1, S1), well volume 0 µL
Change pipettes
Aspirate 5000 nL from (P3, C18, R1, S1), well volume 0 µL, manual

height: 0.5 mm, piston speed: 3 mm/s
Dispense 5000 nL to (P5, C18, R1, S1), well volume 0 µL
Change pipettes
Aspirate 5000 nL from (P3, C19, R1, S1), well volume 0 µL, manual height: 0.5 mm, piston speed: 3 mm/s
Dispense 5000 nL to (P5, C19, R1, S1), well volume 0 µL
Change pipettes
Aspirate 5000 nL from (P3, C20, R1, S1), well volume 0 µL, manual height: 0.5 mm, piston speed: 3 mm/s
Dispense 5000 nL to (P5, C20, R1, S1), well volume 0 µL
Change pipettes
Aspirate 5000 nL from (P3, C21, R1, S1), well volume 0 µL, manual height: 0.5 mm, piston speed: 3 mm/s
Dispense 5000 nL to (P5, C21, R1, S1), well volume 0 µL
Change pipettes
Aspirate 5000 nL from (P3, C22, R1, S1), well volume 0 µL, manual height: 0.5 mm, piston speed: 3 mm/s
Dispense 5000 nL to (P5, C22, R1, S1), well volume 0 µL
Change pipettes
Aspirate 5000 nL from (P3, C23, R1, S1), well volume 0 µL, manual height: 0.5 mm, piston speed: 3 mm/s
Dispense 5000 nL to (P5, C23, R1, S1), well volume 0 µL
Change pipettes
Aspirate 5000 nL from (P3, C24, R1, S1), well volume 0 µL, manual height: 0.5 mm, piston speed: 3 mm/s
Dispense 5000 nL to (P5, C24, R1, S1), well volume 0 µL
