# Supplementary material for: Single-nucleus RNA-seq2 reveals functional crosstalk between liver zonation and ploidy
Source: Nat Commun. 2021 Jul 12;12:4264. doi: 10.1038/s41467-021-24543-5 (PMC8275628; doi:10.1038/s41467-021-24543-5)
Supplement: Supplementary file 17 — Reporting Summary [file 41467_2021_24543_MOESM17_ESM.pdf]

nature research

Corresponding author(s): Celia P. Martinez-Jimenez; Maria Colomé-Tatché

Last updated by author(s): 29/April/21

Reporting Summary

Nature Research wishes to improve the reproducibility of the work that we publish. This form provides structure for consistency and transparency in reporting. For further information on Nature Research policies, see our Editorial Policies and the Editorial Policy Checklist.

Statistics

For all statistical analyses, confirm that the following items are present in the figure legend, table legend, main text, or Methods section.

n/a	Confirmed	
☐	☒	The exact sample size (*n*) for each experimental group/condition, given as a discrete number and unit of measurement
☐	☒	A statement on whether measurements were taken from distinct samples or whether the same sample was measured repeatedly
☐	☒	The statistical test(s) used AND whether they are one- or two-sided<br>*Only common tests should be described solely by name; describe more complex techniques in the Methods section.*
☐	☒	A description of all covariates tested
☐	☒	A description of any assumptions or corrections, such as tests of normality and adjustment for multiple comparisons
☐	☒	A full description of the statistical parameters including central tendency (e.g. means) or other basic estimates (e.g. regression coefficient) AND variation (e.g. standard deviation) or associated estimates of uncertainty (e.g. confidence intervals)
☐	☒	For null hypothesis testing, the test statistic (e.g. *F*, *t*, *r*) with confidence intervals, effect sizes, degrees of freedom and *P* value noted<br>*Give P values as exact values whenever suitable.*
☒	☐	For Bayesian analysis, information on the choice of priors and Markov chain Monte Carlo settings
☒	☐	For hierarchical and complex designs, identification of the appropriate level for tests and full reporting of outcomes
☐	☒	Estimates of effect sizes (e.g. Cohen's *d*, Pearson's *r*), indicating how they were calculated

Our web collection on statistics for biologists contains articles on many of the points above.

Software and code

Policy information about availability of computer code

Data collection

- RNAscope images were scanned using the Zeiss AxioScan Z1 system at 40x magnification.
- Immunofluorescence images were acquired from Zeiss 800 upright confocal microscope.
- FlowJo 10.7.1 for initial QC and data visualization.
- IDEAS v6 for data visualization in Imagestream X MK II.

Data analysis

- For the pre-processing of single cell data obtained from plates, raw reads were aligned to mm10 and ERCC92 reference genome using STARa2.7.1a. PCR duplicates were removed using picard tools v2.20.2 before building the count matrix using htseq-count v0.11.3
- For any data produced using the 10X technology, we used cellranger v4.0.0 to align reads and build the count matrix
- For the downstream analysis we used: python (v3.7.6) , scanpy (v1.6.0 & v1.4.5.2.dev6+gfa408dc7), AnnData (v0.7.4 & v0.7.1), pandas (v1.0.3 & v1.1.0), numpy (v1.18.1 & v1.18.5), gprofiler-official (v1.0.0), matplotlib (v3.2.1), venn (v0.1.2), seaborn (v0.10.0), scipy (v1.4.1)

The code used for downstream analyses is deposited at http://github.com/celiamtnez/snRNA-seq2_young. The version of the code at the time of publication is stored in Zenodo: http://doi.org/10.5281/zenodo.4694749

- RNAscope images were analysed using the Indica Labs Halo
- Immunofluorescence images were analysed using the Fiji Image J Software version 1.0

For manuscripts utilizing custom algorithms or software that are central to the research but not yet described in published literature, software must be made available to editors and reviewers. We strongly encourage code deposition in a community repository (e.g. GitHub). See the Nature Research guidelines for submitting code & software for further information.

Data

Policy information about availability of data

All manuscripts must include a data availability statement. This statement should provide the following information, where applicable:

- Accession codes, unique identifiers, or web links for publicly available datasets
- A list of figures that have associated raw data
- A description of any restrictions on data availability

Raw data is publicly available under: http://www.ebi.ac.uk/arrayexpress/experiments/E-MTAB-9333
Additional publicly available datasets used for comparison can be found under: GSE84498, GSE124395, GSE148339, https://doi.org/10.6084/m9.figshare.5829687.v7 and https://doi.org/10.6084/m9.figshare.5968960.v2 (These comparisons are included in Figure 1C, and Supp. Fig. 1F).

Field-specific reporting

Please select the one below that is the best fit for your research. If you are not sure, read the appropriate sections before making your selection.

- [x] Life sciences
- [ ] Behavioural & social sciences
- [ ] Ecological, evolutionary & environmental sciences

For a reference copy of the document with all sections, see nature.com/documents/nr-reporting-summary-flat.pdf

Life sciences study design

All studies must disclose on these points even when the disclosure is negative.

Sample size	Mice livers for sequencing:<br>- 4 biological replicates (mice) were used in this study, from which a total of 11 plates were obtained (of which 2 were technical replicates). We have sequenced on average 624 single nuclei per mouse and focus our statistics on the single nucleus level. With our data, we have a power of 0.98 to detect at least 5 nuclei of cell types that have a frequency of 2%.<br>- To demonstrate our novel snRNAseq2 approach in a diseased context, 1 each of vehicle- or CCl4-treated mouse were sequenced to study livers with fibrosis. A total of 2 plates were obtained, from each plate, half contains nuclei from vehicle-treated and the other half contains nuclei from CCl4-treated replicate; within each half contains 2n and 4n ploidy nuclei.<br><br>Image analyses:<br>- At least 200 nuclei per condition were analysed to measure Cyp2e1-2 and Cyp2f2 expression in RNAscope experiments.<br>- Percentage of Lgr5 positive nuclei were determined from a total of 100 nuclei.<br>- Number of Lgr5 copies per 2n or 4n nucleus were scored from a total of ~1000 nuclei each using Halo software.<br>- Endothelial cell size was determined from ~200 Lyve1-positive nuclei using Fiji Image J software.
Data exclusions	As deriving statistics from technical replicates is discouraged, we excluded the 2 technical replicates from downstream analysis after normalization. This was done to avoid any bias driven by assessment of the same nuclei, i.e. only the data from the first experiment performed on these nuclei was kept.
Replication	Sequencing:<br>- Clustering of cells was done, showing no noticable difference between the 4 healthy young biological replicates. Results from the transcriptomic data analysis were validated using RNAscope with probes for representative mRNA molecules.<br>- One replicate of Vehicle- or CCl4-treated replicate were used to further demonstrate the application of snRNAseq2 in diseased liver model.<br><br>Imaging:<br>- Since liver zonation has been well characterized by previous independent studies, we analysed liver zonation with RNAscope probes (Cyp2e1-2, Cyp2f2 and Lgr5) in 3 mice livers.<br>- Staining for LSECs was performed from one liver section.<br><br>Imagestream:<br>- 2 young healthy mice livers were used for Imagestream analysis.
Randomization	All young healthy mice used in this study were treated the same, so no randomization was needed.
Blinding	All mice used in this study were treated the same, so no blinding was needed.

Reporting for specific materials, systems and methods

We require information from authors about some types of materials, experimental systems and methods used in many studies. Here, indicate whether each material, system or method listed is relevant to your study. If you are not sure if a list item applies to your research, read the appropriate section before selecting a response.

Materials & experimental systems

n/a	Involved in the study	
☐	☒	Antibodies
☒	☐	Eukaryotic cell lines
☒	☐	Palaeontology and archaeology
☐	☒	Animals and other organisms
☒	☐	Human research participants
☒	☐	Clinical data
☒	☐	Dual use research of concern

Methods

n/a	Involved in the study	
☒	☐	ChIP-seq
☐	☒	Flow cytometry
☒	☐	MRI-based neuroimaging

Antibodies

Antibodies used

DAPI (ThermoFisher, #62248, Lot. UE2771402), used at 1:1000
Hoechst 33342 (Thermo Fisher Scientific, Cat No. 62249), 10µg/mL
Anti-Lyve1 (Abcam, EPR21771, Lot. GR3357898-4), used at 1:1000
Anti-β-catenin (Novus Biologicals, cat.AF1329-SP, Lot. H2TU919091), used at 1:50
Donkey anti-Goat Alexa555 IgG (H+L) (ThermoFisher, A-21432), used at 1:1000
Donkey anti-Rabbit Alexa647 IgG (H+L) (Thermofisher, A-31573), used at 1:1000
a-SMA-Cy3 conjugated (Sigma, clone 1a4, Lot. 037m4783v). See citation for usage.
RNAscope® 2.5 LS Probe Mm-Cyp2f2 (Cat No. 451858)
RNAscope® 2.5 LS Probe Mm-Cyp2e1-C2 (Cat No. 402788 -C2)
RNAscope® 2.5 LS Probe Mm-Lgr5 (Cat No. 312178)
Pan-centromeric probe (Cambio, Cat 1697-MF-01)
TSA plus-Cy5 (Akoya Biosciences Cat No. NEL745001KT), detection at 1:500 dilution of β-catenin protein
TSA plus-Cy3 (Akoya Biosciences Cat No. NEL744001KT), detection at 1:500 dilution of Cyp2e1-2 or Lgr5
TSA plus -Fluorescein (Akoya Biosciences Cat No. NEL741001KT), detection at 1:500 dilution of Cyp2f2

Validation

All antibodies were validated by respective manufacturers (see websites).

Animals and other organisms

Policy information about studies involving animals; ARRIVE guidelines recommended for reporting animal research

Laboratory animals

Young (3 months old) male mice (C57Bl6/J) were used; furthermore, CCl4-treated C57Bl6/J mice were used to model fibrosis. All mice were maintained in a specific pathogen-free environment in Individually Ventilated Caging units. Mice were kept in a positive pressure system, maintaining a temperature between 19 and 23 degrees, 55% humidity (± 10%), 20 total air changes per hour, and under a 12 hours light/dark cycle. Mice had free access to standard laboratory diet (PicoLab Mouse Diet 20, 5R58) and water.

Wild animals

The study did not involve wild animals

Field-collected samples

The study did not involve samples collected from the field

Ethics oversight

This investigation was approved by the Animal Welfare and Ethics Review Board and followed the Cambridge Institute guidelines for the use of animals in experimental studies under Home Office licences PPL 70/7535 until February 2018 and PPL P9855D13B from March 2018. All animal experimentation was carried out in accordance with the Animals (Scientific Procedures) Act 1986 (United Kingdom) and conformed to the Animal Research: Reporting of In Vivo Experiments (ARRIVE) guidelines developed by the National Centre for the Replacement, Refinement and Reduction of Animals in research (NC3Rs)

Note that full information on the approval of the study protocol must also be provided in the manuscript.

Flow Cytometry

Plots

Confirm that:

- ☒ The axis labels state the marker and fluorochrome used (e.g. CD4-FITC).
- ☒ The axis scales are clearly visible. Include numbers along axes only for bottom left plot of group (a 'group' is an analysis of identical markers).
- ☒ All plots are contour plots with outliers or pseudocolor plots.
- ☒ A numerical value for number of cells or percentage (with statistics) is provided.

Methodology

Sample preparation

Sample preparation is described in detail in the methods and supplementary information sections of the manuscript.

Instrument	- Isolation of nuclei and data acquisition was performed using BD FACSAria III (BD Bioscience).<br>- ImageStream®X Mk II was used for acquire Imagestream datasets.
Software	- Flow cytometry data were analyzed using BD FACSDiva 6.1 and FlowJo 10.7.1 software.<br>- Imagestream data were analyzed using IDEAS v6.
Cell population abundance	All sortings are >98% purity, determined by the sorting cytometer.<br>Individual nuclei were sorted into each well of well plates for sequencing.<br><br>Quantification of nuclear ploidy using Imagestream:<br>- 15,000 - 30,000 2n nuclei<br>- 15,000 - 30,000 4n nuclei<br>- 1,000 - 2,500 8n nuclei
Gating strategy	Preliminary gating strategy for intact single nuclei were made using FSC-A/SSC-A, followed by FSC-A/FSC-H, then by FSC-A/FSC-W. Hoechst staining was used to distinguish intact single nuclei showing different ploidy status. Events abobve 10^4 are considered Hoechst positive. For sorting strategy and quantification on Imagestream, see Supplementary Figure 9 and 13.

☒ Tick this box to confirm that a figure exemplifying the gating strategy is provided in the Supplementary Information.
